# Disaster preparedness and service continuity in primary healthcare centers: a systematic review and structured contextual evidence synthesis

**DOI:** 10.3389/fpubh.2026.1961009

**Published:** 2026-10-28

**Authors:** Siska Nia Irasanti, Deni Kurniadi Sunjaya, Guswan Wiwaha, Titik Respati, Roy Rillera Marzo

**Affiliations:** 1 Doctoral Study Program in Medical Science, Faculty of Medicine, Universitas Padjadjaran, Sumedang, Indonesia; 2 Department of Public Health, Faculty of Medicine, Universitas Islam Bandung, Bandung, Indonesia; 3 Department of Public Health, Faculty of Medicine, Universitas Padjadjaran, Sumedang, Indonesia; 4 Faculty of Health Sciences, Curtin University Malaysia, Miri, Sarawak, Malaysia

**Keywords:** health emergency and disaster risk management, primary healthcare center disaster preparedness, primary healthcare resilience, service continuity, structured contextual evidence synthesis, systematic review

## Abstract

**Background:**

Primary healthcare centers (PHCs) are a key health-system contact for disaster-affected populations, yet direct PHC evidence remains thin and is frequently conflated with hospital, workforce, policy, and secondary-review literature.

**Objective:**

To identify factors, barriers, facilitators, capacities, and operational gaps shaping PHC disaster preparedness; contrast component profiles against a structured contextual synthesis; characterize what is operationally distinctive about PHCs; and propose a PHC-derived layered model.

**Methods:**

A systematic review, supplemented by a structured contextual evidence synthesis, was conducted following the Preferred Reporting Items for Systematic Reviews and Meta-Analyses (PRISMA) 2020 statement and Synthesis Without Meta-analysis (SWiM) principles. The primary Scopus search plus preserved supplementary retrieval from PubMed, ProQuest, and OpenAlex, followed by a pre-submission full-text update, duplicate reconciliation, and independent confirmation by two reviewers, yielded 74 unique publications: 15 primary empirical PHC studies and 59 contextual publications. Evidence was mapped descriptively to the 10 World Health Organization Health Emergency and Disaster Risk Management (Health EDRM) components; all 15 primary PHC studies were independently appraised by two reviewers using design-specific Joanna Briggs Institute (JBI) tools or the Mixed Methods Appraisal Tool (MMAT) 2018.

**Results:**

All 10 Health EDRM components were assessed across the 15 primary PHC studies. The most frequently identified were human resources (14/15, 93.3%), planning and coordination (13/15, 86.7%), information and knowledge management (11/15, 73.3%), followed by health infrastructure and logistics and monitoring and evaluation (each 10/15, 66.7%). Distinctive PHC characteristics included small generalist teams, staff and family-readiness barriers, executable plans, referral dependence, continuity of basic utilities and water, sanitation, and hygiene (WASH) services, limited local stocks, community outreach, and regular facility-level monitoring.

**Conclusion:**

PHC preparedness emerges as an interdependent capacity, with several distinctive PHC characteristics arising from specific Health EDRM components. Community capacities for Health EDRM were reported in only 40% of primary PHC studies, highlighting an under-reported area warranting further exploration. A layered model is proposed for center-level assessment and future instrument development, organizing system support, core preparedness capacity, service and community response, and cross-cutting information, communication, and monitoring as interacting domains linked to continuity of essential services.

**Systematic review registration:**

Open Science Framework, https://doi.org/10.17605/OSF.IO/H8JU7, Identifier: 10.17605/OSF.IO/H8JU7.

## 1 Introduction

Disasters, epidemics, climate-related extreme events, conflict, and other public-health emergencies disrupt access to care exactly when population health needs peak. Primary healthcare centers (PHCs) are multidisciplinary first-contact organizations located close to communities and local authorities. They commonly provide preventive care, routine treatment, maternal and child health services, surveillance, vaccination, chronic-disease care, referral, outreach, and basic emergency response. Their ability to remain operational therefore influences both immediate response and continuity of essential services. The World Health Organization (WHO) Health Emergency and Disaster Risk Management (Health EDRM) framework organizes this capacity through 10 interrelated components: policies, strategies and legislation; planning and coordination; human resources; financial resources; information and knowledge management; risk communications; health infrastructure and logistics; health and related services; community capacities; and monitoring and evaluation (1). These functions are consistent with risk-informed governance and preparedness principles in the Sendai Framework (2).

Preparedness of PHCs cannot be inferred directly from hospital preparedness. Hospitals usually operate with specialized teams, formal command structures, inpatient beds, advanced diagnostic and therapeutic capacity, and greater infrastructure redundancy. PHCs, including Puskesmas, Indonesian public primary healthcare centers that provide first-contact clinical and population-health services within defined catchment areas, as well as community health centers, public health centers, rural health centers, and comparable multidisciplinary centers, more often depend on smaller generalist teams, basic utilities, limited storage, referral networks, district-level support, and close interaction with a defined catchment population.

Previous reviews have described broad primary-care preparedness domains, climate-related preparedness among PHC nurses, provider roles for vulnerable populations, emergency water supply, healthcare-worker preparedness, and preparedness education (3–8). However, three issues remain unresolved: center-level empirical PHC evidence has not been consistently separated from workforce, hospital, policy, and secondary-review evidence; preparedness functions have not been consistently linked to continuity of essential services; and the evidence-to-domain pathway for PHC assessment development remains insufficiently explicit. The present review addresses these gaps by isolating empirical PHC studies, using broader evidence only for structured contextual interpretation, and applying a transparent evidence-to-domain process to develop a PHC-oriented functional model linked to continuity outcomes.

A focused synthesis of empirical PHC studies is needed to characterize center-level preparedness, while broader health-system evidence can help interpret functions that may be transferable to PHC practice. We therefore separated the primary PHC systematic synthesis from a structured contextual synthesis used only for interpretive triangulation and model development; the detailed evidence architecture is described in the Methods.

This review aimed to synthesize center-level empirical evidence on PHC disaster preparedness and service continuity and to develop an evidence-informed functional model. It addressed four questions: (1) What preparedness factors and operational gaps are reported in empirical PHC studies? (2) How do Health EDRM reporting patterns in PHC studies compare descriptively with the contextual evidence set? (3) Which operational characteristics are particularly relevant to PHCs? and (4) How can the findings be organized into a PHC-oriented model linked to continuity of essential services?

## 2 Materials and methods

### 2.1 Review design and reporting framework

This study comprised a systematic review of primary empirical PHC studies and a structured contextual evidence synthesis used for interpretive triangulation and model development. The contextual set included broader health-system, policy, workforce, hospital, community, and secondary-review publications only when their reported functions were potentially transferable to PHC or district practice. It was not treated as a second systematic review, pooled analysis, or source of direct PHC effect estimates. The focused review used a population-exposure-comparison-outcome orientation and was reported according to PRISMA 2020 and SWiM principles (9, 10). Generative artificial intelligence was used only for final language editing, consistency checking, formatting, and table presentation; no eligibility, extraction, appraisal, coding, or interpretation decision was delegated to the tool.

### 2.2 Protocol and registration

The review was conducted under a predefined protocol and was registered retrospectively on the OSF after screening, extraction, and initial synthesis. The final protocol, search documentation, amendment log, extraction matrices, critical-appraisal records, archived original Watase output, and audited PRISMA flow were deposited in a time-stamped retrospective Open Science Framework (OSF) registration. The record is explicitly identified as retrospective rather than preregistration. To mitigate *post-hoc* selection and reporting bias, all final eligibility, retrieval, appraisal, coding, and synthesis decisions were rechecked against the prespecified criteria, independently confirmed by two reviewers, and documented in the time-stamped amendment and consensus records. Retrospective OSF registration: https://doi.org/10.17605/OSF.IO/H8JU7. Associated OSF project: https://osf.io/7atku/

### 2.3 Review question and population-exposure-comparison-outcome (PECO) orientation

The review question was: What factors are reported in relation to disaster preparedness and service continuity in primary healthcare centers? The population or setting comprised PHCs and personnel working in PHCs. Exposures included knowledge, training, experience, leadership, plans, standard operating procedures, coordination, logistics, infrastructure, financing, information, risk communication, and contextual barriers or facilitators. Comparisons were retained when reported but were not mandatory. Eligible outcomes included disaster preparedness, emergency readiness, response capacity, resilience, center safety, and continuity of essential services. In this review, factors related to preparedness were operationalized as reported barriers, facilitators, capacities, and operational gaps; the term does not imply causality or a statistically tested association.

### 2.4 Information sources and search strategy

The primary bibliographic search was conducted in Scopus, and supplementary searches were conducted in PubMed, ProQuest, and OpenAlex. The PubMed search was conducted on 27 June 2026, and the Scopus, ProQuest, and OpenAlex searches were conducted on 30 June 2026. Reference lists of eligible and highly relevant publications were also checked during screening, but a separate citation-search yield was not preserved and no number was reconstructed. Search concepts combined disaster-preparedness terms and international labels for PHCs, including primary health care, primary healthcare, primary care center/centre, community health center/centre, public health center/centre, rural health center/centre, and Puskesmas. A targeted pre-submission full-text retrieval and bibliographic update was completed in July 2026. The review therefore covered eligible publications from 2016 through the final July 2026 update.

The core search combined disaster preparedness, emergency preparedness, emergency planning, health emergency, disaster readiness, facility preparedness, resilience, and continuity of essential services with PHC-setting terms. Syntax was adapted to each database. Scopus used title, abstract, and keyword fields; PubMed used title/abstract terms and relevant Medical Subject Headings; ProQuest used abstract and subject fields where available; and OpenAlex used keyword- and concept-based retrieval. Complete database-specific strings are reported in Supplementary Table S1.

Targeted supplementary searches were conducted in ProQuest, PubMed, and OpenAlex to identify potentially relevant additional publications. OpenAlex was included as an open, multidisciplinary scholarly index to broaden discovery beyond the coverage and indexing structures of the other sources and to identify potentially relevant records not retrieved through Scopus, PubMed, or ProQuest (11). It served only as a supplementary discovery source and did not replace the primary Scopus search. The available contemporaneous records documented that the ProQuest search initially returned 1,158 records, which was reduced to 129 after application of the 10-year publication-period limit. Subsequent source-type and subject-area limits produced 30 records for final relevance and eligibility assessment, from which 10 publications were retained. For PubMed and OpenAlex, the available records confirmed that 10 and 6 publications, respectively, were retained, although the intermediate screening stages were documented less comprehensively than for ProQuest. The 26 supplementary publications were assessed for relevance and evidence role and reconciled by title and DOI with the Scopus-derived evidence base. Accordingly, this pathway was treated as a targeted evidence supplement rather than a set of parallel systematic searches equivalent to the primary Scopus search.

### 2.5 Eligibility criteria and evidence architecture

Primary studies were eligible when they reported empirical quantitative, qualitative, or mixed-methods findings from an eligible PHC or PHC workforce and examined disaster preparedness, emergency readiness, safety, response capacity, resilience, or service continuity. Team-based primary care practices were eligible only when they functioned as multidisciplinary, first-contact centers with defined population-health or community responsibilities. Solo general-practitioner offices and hospital outpatient departments were excluded because they did not meet these center-level criteria. Eligible publications were English-language, peer-reviewed full-text articles published from 2016 through the final July 2026 update. Hospital-only studies, emergency departments, specialist units, and studies without a relevant preparedness or continuity outcome were excluded. Journal quartile was not an eligibility criterion; an original Q1–Q4 journal-tier setting that temporarily removed 24 records was corrected through the audit described in Section 2.6.

Fifteen primary empirical PHC studies were retained within the audited 74-publication evidence base. The remaining 59 publications formed the contextual set and were explicitly classified as non-primary PHC evidence. Additional contextual publications included in the structured synthesis are cited here (50–82); their publication-level evidence roles and component coding are documented in Supplementary Tables S8–S11. Transferability was assessed before component-frequency tabulation and independently of how frequently a Health EDRM component occurred. A contextual publication was retained only when the source-reported preparedness function could reasonably be implemented through, coordinated with, or supported at PHC or district level and did not depend exclusively on tertiary-care infrastructure, specialist-only services, or hospital-specific surge capacity. Examples of transferable functions included health-system financing that enables local preparedness, community-engagement mechanisms linked to risk communication and service access, and community-health-worker roles in outreach and continuity during crises. Transferability decisions were independently reviewed by two reviewers and resolved by consensus. Contextual publications were used for descriptive comparison, identification of transferable practices, interpretation of under-reported components, and development of the proposed model, but not to alter the primary PHC denominator, PHC component frequencies, methodological-confidence profile, or causal interpretation.

### 2.6 Study selection, deduplication, and bibliographic reconciliation

Search records were managed using Watase, a web-based literature-screening and systematic-review management platform (12) and supporting spreadsheets. In the original screening workflow, 24 records were temporarily omitted by a Q1–Q4 journal-tier setting that was not part of the prespecified eligibility criteria. Throughout this manuscript, original refers to the workflow conducted before the final audit, archived refers to records preserved from that workflow, and audited or revised refers to the evidence base after reconstruction, reassessment, retrieval updating, and bibliographic reconciliation. During the final audit, all 24 records were reconstructed and independently reassessed by SNI and TR: one primary PHC study was included, and 23 records were excluded for record-specific reasons (Supplementary Table S3). The original title/abstract and full-text eligibility decisions were subsequently independently audited by SNI and TR against the prespecified criteria; therefore, this process is reported as a two-reviewer audit rather than prospective duplicate screening. Disagreements were resolved through source rechecking, discussion, and consensus. The retrieval update and filter audit followed the same independent review and consensus procedure.

The original evidence matrix contained 67 retained records: 41 from the primary Scopus workflow and 26 from supplementary database searches. A bibliographic audit consolidated one duplicate entry and two preprint/final-publication pairs, yielding 64 unique publications. A subsequent pre-submission retrieval update secured and locally verified full-text copies for 11 of the 32 reports originally recorded as not retrieved. One file was an erratum, and one substantive report did not examine healthcare, health-service, or PHC preparedness context. Nine additional unique publications met the evidence-architecture criteria after independent reassessment by two reviewers and digital object identifier (DOI) and title duplicate checking, producing 73 unique publications. The final Q1–Q4 filter audit then added one eligible primary PHC study from the 24 reconstructed records, with no duplicate identity detected, yielding a final evidence base of 74 unique publications (15 primary empirical PHC studies and 59 contextual publications). The complete retrieval ledger, tier-filter audit, and evidence-base reconciliation are provided in Supplementary Tables S2–S4.

### 2.7 Data extraction and health EDRM coding

A standardized extraction matrix captured author, year, country, study design, PHC type, sample, hazard context, instrument, reported factor or operational gap, principal finding, limitation, and Health EDRM mapping. The 10 Health EDRM components formed the deductive framework, while specific subcomponents and indicators remained article derived. A component was coded as present when a publication reported at least one relevant factor, measured domain, operational gap, or preparedness indicator; each publication was counted once per component. The primary PHC and contextual sets were coded separately. Initial extraction and Health EDRM coding were performed by SNI and subsequently audited against the full texts by TR. Disagreements were resolved through discussion, re-examination of the relevant passages, and consensus, with final decisions and source locations recorded in the extraction and coding matrices.

Comparative occurrence was reported for primary PHC studies (*n* = 15), contextual publications (*n* = 59), and all mapped publications (*n* = 74). Percentage-point differences described pattern divergence only. Statistical significance tests were not performed because the evidence sets differed in eligibility, design, and unit of evidence and were not conceptualized as independent samples.

### 2.8 Methodological quality appraisal

Methodological appraisal was centered on the 15 primary empirical PHC studies and was design specific. Qualitative, analytical cross-sectional, and prevalence/descriptive facility-assessment studies were assessed using the relevant Joanna Briggs Institute (JBI) critical-appraisal checklist (13). The Mixed Methods Appraisal Tool (MMAT) 2018 (14) was used for mixed-methods and quantitative descriptive studies when appropriate. For studies with internally inconsistent design reporting, we appraised using the design self-identified in the article’s methods section and disclosed the inconsistency in the appraisal log (Supplementary Table S6). All 15 primary empirical PHC studies were independently appraised from the complete full text by SNI and TR. Each appraiser completed item-level judgments independently using Yes, No, Unclear, or Not applicable; differences were resolved through full-text rechecking and consensus. No numerical cut-off was used to create official JBI grades, and no overall MMAT score was calculated. Narrative methodological judgments reflect the pattern and importance of design limitations rather than official JBI or MMAT quality categories. The heterogeneous contextual set was classified by evidence type and used for descriptive triangulation and model interpretation; it was not pooled into a directly comparable quality distribution. Appraisal procedures and the design-specific summary are provided in Supplementary Table S6, and item-level consensus judgments are provided in Supplementary Table S7.

### 2.9 Pre-submission full-text retrieval update

All 32 reports initially recorded as not retrieved in Watase were rechecked using title and DOI information. Retrieval was attempted through publisher platforms, the institutional subscriptions available to the review team, and other legitimate access routes. Full-text copies were obtained and locally verified for 11 reports. The files comprised one erratum and 10 substantive publications. The 10 substantive reports were assessed against the existing eligibility criteria, appraised using JBI or MMAT according to design, and mapped to the 10 Health EDRM components. One community-preparedness article was excluded because it did not examine a healthcare facility, PHC workforce, health-system preparedness function, or continuity of health services. Nine reports were added after duplicate-identity verification. For the remaining 21 report records, bibliographic and publisher records were identified, but full-text copies could not be secured for local verification through the legitimate access options available to the review team at the time of the update, including where access was restricted by subscription or institutional availability. The complete 32-report ledger, reasons for the two new exclusions, and the item-level audit of the retrieved reports are provided in Supplementary Tables S2, S7.

### 2.10 Development of the proposed layered model

The 10 Health EDRM components were predefined as the deductive coding framework, whereas the four higher-order functional domains were not predefined and were not adopted from an existing model. After completion of both syntheses, the authors reviewed the source-derived indicators within each component and inductively proposed candidate groupings according to their functional role in PHC preparedness and service continuity. Two reviewers independently assigned the components to these candidate domains using four explicit criteria: consistency with the WHO component definition, relevance to center-level PHC practice, proximity to minimum operational continuity, and coherence with the source-derived indicators. Component frequencies were calculated to describe reporting prominence, assess the density of supporting evidence, and identify under-reported areas. Frequencies informed the interpretation of the model but did not determine domain formation or component placement. For example, information and knowledge management was among the most frequently reported components but was classified as a cross-cutting function because it supports all domains. Assignments and supporting evidence were compared, disagreements were resolved through source rechecking and consensus, and the placement rationale was documented in Supplementary Table S14. The resulting layered model is therefore an author-derived conceptual organization of the reviewed evidence, not an externally validated framework or tested causal model.

## 3 Results

### 3.1 Study selection, retrieval update, and final evidence-base reconciliation

The Scopus search identified 201 records. After deduplication, application of the publication period, and the original screening pathway, 74 reports were sought for retrieval. The July 2026 update obtained 11 of 32 reports previously recorded as not retrieved; nine eligible unique publications were added. The subsequent audit of 24 records affected by the original Q1–Q4 journal-tier setting identified one additional eligible primary PHC study. After reconciliation of duplicate and alternative-version records, the final evidence base comprised 74 unique publications: 15 primary empirical PHC studies and 59 contextual publications (Figure 1, Table 1, Supplementary Tables S2–S4).

**Figure 1:**
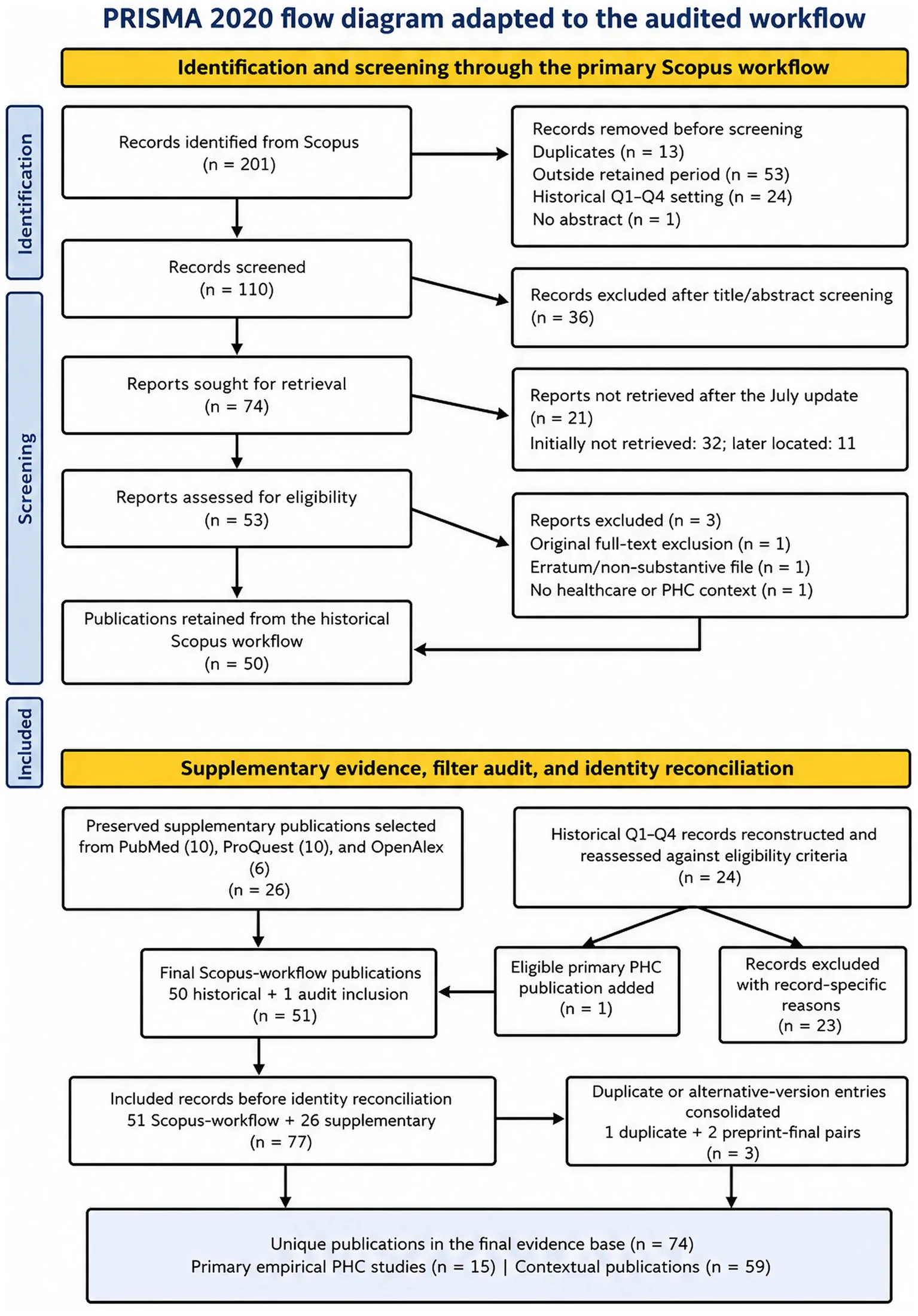
Preferred Reporting Items for Systematic Reviews and Meta-analyses (PRISMA) 2020 flow diagram adapted from the official template for the audited study-selection workflow. The diagram reports the original Scopus identification and screening pathway, the July 2026 retrieval update, the reassessment of all 24 records affected by the original Q1–Q4 journal-tier setting, the preserved set of 26 publications selected from supplementary database searches, and final identity reconciliation. The supplementary counts are selected publications rather than unfiltered database yields because complete source-specific screening flows were not preserved. Final eligibility was determined by the prespecified study-level criteria. Adapted from the PRISMA 2020 flow diagram (Page et al. (9)), under CC BY 4.0 (https://creativecommons.org/licenses/by/4.0/). Source: https://www.prisma-statement.org/prisma-2020-flow-diagram. The layout and counts were adapted to the audited review workflow.

**Table 1:** PRISMA 2020 study-selection, retrieval-update, and evidence-base reconciliation summary.

Selection stage	*n*
Records identified through primary Scopus search	201
Duplicates removed before screening	13
Records removed outside the 2016–2026 period	53
Records temporarily not retained under original Watase Q1–Q4 journal-tier setting	24
Record without abstract	1
Records screened by title and abstract in original workflow	110
Records excluded after title/abstract screening	36
Full-text reports sought	74
Initially assessed in full text	42
Initially not retrieved	32
Full-text reports obtained and locally verified during retrieval update	11
Full-text reports not retrieved after update	21
Final full-text reports assessed in original workflow	53
Full-text reports excluded across both stages (1 initial assessment + 2 retrieval update)	3
Studies retained after retrieval update	50
Tier-filtered records reconstructed and reassessed	24
Primary PHC study included after tier-filter audit	1
Tier-filtered records excluded against eligibility criteria	23
Final studies retained from Scopus workflow	51
Preserved supplementary publications selected and reconciled	26
Combined included records before identity reconciliation	77
Duplicate/alternative-version entries consolidated	3
Final unique publications	74
Primary empirical PHC studies	15
Contextual publications	59

PHC, primary healthcare center; Q1–Q4, journal quartiles 1–4. During the original Watase pre-screening process, 24 records were temporarily not retained within the selected Scopus Q1–Q4 journal-tier setting before eligibility screening. The final audit reconstructed all 24 record identities and reassessed them against the prespecified eligibility criteria. One mixed-methods study of cholera outbreak preparedness in all 101 public health centers in Addis Ababa was included as a primary PHC study, and 23 records were excluded with record-specific reasons. The July 2026 retrieval update separately obtained and locally verified 11 of the 32 reports initially recorded as not retrieved. For the remaining 21 report records, bibliographic and publisher records were identified, but full-text copies could not be secured for local verification through the legitimate access options available to the review team at that stage, including subscription- or institution-restricted access. One report identity was already represented by a separately obtained supplementary-source copy; full-text eligibility assessment could not be completed for the other 20 report identities. Three reports were excluded in the retrieval pathway: one during the original full-text assessment, one erratum, and one report outside the healthcare/PHC context. Detailed retrieval, filter-audit, and reconciliation data are provided in Supplementary Tables S2–S4.

The retained supplementary pathway contributed 26 publications: PubMed (*n* = 10), ProQuest (*n* = 10), and OpenAlex (*n* = 6). These were reconciled with the 51 studies retained through the corrected Scopus workflow. Three duplicate or alternative-version records were consolidated before the final total of 74 unique publications was established.

The 15 primary studies represented varied PHC settings, hazards, designs, and sample sizes. Detailed study characteristics, methodological appraisal, the functional arrangement of the proposed layered model, and comparative component frequencies are summarized in Tables 2–5, respectively. Design-specific appraisal details are also provided in Section 3.2 and Supplementary Tables S6, S7.

**Table 2:** Characteristics and principal findings of the 15 primary empirical studies of primary healthcare centers.

Study/country	Design/sample	Hazard/context	Main factor focus and Health EDRM mapping	Principal limitation
Alrowili et al. (25); Saudi Arabia	Mixed methods; *n* = 400	All hazards	Technical knowledge, simulation, teamwork, emotional intelligence, and stress management. Mapping: HR.	Cross-sectional self-report; exploratory predictive modelling.
Dg Sirua et al. (40); Indonesia	Analytical cross-sectional; 125 nurses from 14 public health centers	Disaster-prone PHC settings	Policy/planning competence, knowledge and training, structural resilience, continuity of PHC services and multisector/community collaboration. Mapping: PSL; PC; HR; IKM; HIL; HRS; CC.	Cross-sectional design; important demographic and organizational confounders were not adjusted.
Derese et al. (18); Ethiopia	Institution-based cross-sectional mixed methods; all 101 public health centers, 5 key-informant interviews, and 2 focus group discussions (FGDs)	Cholera outbreak preparedness	Protocols and guideline implementation, preparedness plans, rapid response teams and trained public health emergency management (PHEM) officers, surveillance/reporting, financing, water, sanitation, and hygiene (WASH), treatment supplies, personal protective equipment (PPE), and multisectoral coordination. Mapping: PSL; PC; HR; FR; IKM; HIL; HRS; ME.	Cross-sectional design; limited qualitative analytic detail and measurement-validation evidence; equally weighted binary threshold; incomplete confounder framework; community risk communication and engagement not assessed.
Zarei et al. (24); Iran	Qualitative focus group discussion (FGD)/case evaluation; 7 experts	Household/PHC readiness education	Guideline usability, cultural adaptation, educational needs, early warning, continuous needs assessment. Mapping: PC; HR; IKM; RC; CC.	Small purposive expert group; evaluation of one national guideline.
Lamberti-Castronuovo et al. (19); Italy	Checklist development and field test; 10 centers	All-hazards PHC preparedness	Multidisciplinary teams, surge staffing, training, PPE, stocks, utilities, incident plan, service integration, community engagement, health information system (HIS), communication. Mapping: PC; HR; IKM; RC; HIL; HRS; CC; ME.	Small purposive sample; checklist not yet psychometrically validated.
Prakash et al. (22); India	Analytical cross-sectional knowledge, attitude, and practice (KAP) survey; *n* = 324	Disaster mental health	Disaster mental-health knowledge, attitudes, and practices; knowledge of psychological first aid and psychosocial-triage concepts; and disaster mental-health planning. Mapping: PC; HR; HRS.	Convenience/snowball sampling; self-report; response rate unavailable.
Fathoni et al. (31); Indonesia	Correlative analytical observational cross-sectional study; quota sample of 120 nurses from 16 PHCs; internally inconsistent intervention/control and pre-post reporting	coronavirus disease 2019 (COVID-19) pandemic	Health protocols, protocol knowledge, staff self-control, and disaster-preparedness questionnaire assessment. Mapping: PC; HR; IKM; ME.	Non-probability quota sampling; internal inconsistency between the stated cross-sectional design and additional intervention/control and pre-post reporting; limited confounder control.
Lestari et al. (26); Indonesia	Quantitative descriptive Hospital Safety Index (HSI) facility assessment using multiple evidence sources; 11 centers	Multi-hazard and pandemic preparedness	Structural/non-structural safety, water, fuel, committee, plan, storage, equipment, training, budgeting. Mapping: PSL; PC; HR; FR; HIL; ME.	Purposive facility selection; limited national representativeness; no separate qualitative analytic strand.
Litke et al. (15); Germany	Qualitative observational; 40 experts; 2 FGDs and 26 interviews	Health-system crises including COVID-19	Communication, employee resilience, organizational change, resource gaps, backup storage and supplies. Mapping: PSL; HR; IKM; HIL.	Perceived strategies rather than observed disaster performance.
Firdausi et al. (32); Indonesia	Multi-source quantitative descriptive HSI assessment; 2 Puskesmas	All-hazards facility preparedness	Governance, plans, staffing, information, warning/communication, utilities, equipment and medicines, essential services, community coordination and monitoring. Mapping: PSL; PC; HR; IKM; RC; HIL; HRS; CC; ME.	Two purposively selected centers; no separately analyzed qualitative strand.
van Hoang et al. (33); Vietnam	Cross-sectional mixed methods; 80 facilities (76 commune/ward health stations and 4 district facilities) plus interviews/FGDs	COVID-19	Planning, staffing, financing, surveillance, communication, infrastructure, clinical/public-health services, community partnerships and evaluation. Mapping: PSL; PC; HR; FR; IKM; RC; HIL; HRS; CC; ME.	Brief qualitative analysis and mixing rationale; representativeness and nonresponse incompletely reported.
Yari et al. (16); Iran	Cross-sectional PHC safety assessment; 805 centers	All-hazards disaster safety	Structural, non-structural and functional safety; training, response team, finance, equipment, water and food. Mapping: PSL; PC; HR; FR; HIL; HRS; CC; ME.	Descriptive assessment; limited causal inference.
Ndumbi et al. (17); Democratic Republic of the Congo	Analytical cross-sectional; 180/374 centers	Cholera outbreak	Protocols, transfer, isolation, WASH, chlorine, sterilization, waste, oral rehydration solution (ORS), Ringer lactate. Mapping: PC; IKM; HIL; HRS; ME.	Hazard-specific indicators; limited generalizability.
Lapcevic et al. (20); Serbia	PHC HSI assessment; 1 center	2014 floods/all hazards	Structural integrity, utilities, telecommunications, plans, staffing, finance, laboratory equipment, 72-h operation. Mapping: PC; HR; FR; IKM; HIL; ME.	Single-center descriptive assessment.
Hashikawa and Gold (23); United States	Qualitative-descriptive survey/interviews; 12 leaders	All hazards	Leadership attitudes, personal plans, threat appraisal, exercises, fear of infection, childcare and pet-care barriers. Mapping: PC; HR; IKM; ME.	Small convenience sample of academic clinics.

CC, community capacities for Health EDRM; COVID-19, coronavirus disease 2019; FGD/FGDs, focus group discussion(s); FR, financial resources; Health EDRM, Health Emergency and Disaster Risk Management; HIL, health infrastructure and logistics; HIS, health information system; HRS, health and related services; HR, human resources; HSI, Hospital Safety Index; IKM, information and knowledge management; KAP, knowledge, attitude, and practice; ME, monitoring and evaluation; ORS, oral rehydration solution; PC, planning and coordination; PHC/PHCs, primary healthcare center(s); PHEM, public health emergency management; PPE, personal protective equipment; PSL, policies, strategies and legislation; RC, risk communications; WASH, water, sanitation, and hygiene.

**Table 3:** Methodological appraisal overview of the 15 primary empirical PHC studies.

Study	Design	Tool	Narrative judgment	Principal concern
Alrowili et al. (25)	Mixed methods	MMAT 2018	Moderate-to-major concerns	Incomplete qualitative audit trail, uncertain representativeness/nonresponse, limited model-validation and integration reporting
Dg Sirua et al. (40)	Analytical cross-sectional survey	JBI analytical cross-sectional	Moderate concerns	Incomplete eligibility criteria; limited validity evidence; mean-based dichotomization; insufficient confounder control
Derese et al. (18)	Cross-sectional mixed methods	MMAT 2018	Moderate concerns	Limited qualitative analytic detail; restricted measurement-validation evidence; equally weighted binary preparedness threshold; incomplete confounder specification; no explicit divergence analysis
Zarei et al. (24)	Qualitative FGD	JBI qualitative	Moderate concerns	Limited reflexivity and participant-voice traceability
Lamberti-Castronuovo et al. (19)	Checklist development/facility assessment	JBI prevalence	Major concerns	Purposive 10-facility sample and unvalidated checklist
Prakash et al. (22)	Analytical cross-sectional KAP	JBI analytical cross-sectional	Moderate-to-major concerns	Convenience snowball sampling, unknown response rate, limited validation, no confounder adjustment
Fathoni et al. (31)	Analytical cross-sectional observational study with internally inconsistent additional reporting	JBI analytical cross-sectional	Major concerns	Quota sampling; limited validation detail; no explicit confounder framework or adjusted analysis; intervention/control and pre-post reporting cannot be reconciled with the stated cross-sectional design
Lestari et al. (26)	Quantitative descriptive multi-site HSI assessment	MMAT quantitative descriptive	Moderate concerns	Purposive 11-facility selection; limited representativeness; facility recruitment denominator not reported
Litke et al. (15)	Qualitative interviews/FGDs	JBI qualitative	Low concerns	Strong sampling, reflexivity, analysis, and quotation-supported findings
Firdausi et al. (32)	Quantitative descriptive two-facility HSI assessment	MMAT quantitative descriptive	Major concerns	Accessibility-based selection of two facilities; incomplete technical expertise among informants; limited generalizability
van Hoang et al. (33)	Cross-sectional mixed methods	MMAT 2018	Moderate concerns	Uncertain representativeness; under-described qualitative analysis; subjective ratings; incomplete inferential reporting; no divergence analysis
Yari et al. (16)	Province-wide facility assessment	JBI prevalence	Low-to-moderate concerns	Broad coverage and standardized tool; assessor reliability incompletely reported
Ndumbi et al. (17)	Analytical cross-sectional facility survey	JBI analytical cross-sectional	Low-to-moderate concerns	Structured sampling and analysis; self-reported case history and residual confounding
Lapcevic et al. (20)	Single-facility HSI audit	JBI Prevalence/facility audit	Major external-validity concerns	Detailed case audit but no population-level generalizability
Hashikawa and Gold (23)	Descriptive mixed methods	MMAT 2018	Moderate concerns	Very small purposive sample and unvalidated survey

FGD/FGDs, focus group discussion(s); HSI, Hospital Safety Index; JBI, Joanna Briggs Institute; KAP, knowledge, attitude, and practice; MMAT, Mixed Methods Appraisal Tool. Narrative judgments are review-defined interpretations and are not official Joanna Briggs Institute (JBI) or Mixed Methods Appraisal Tool (MMAT) grades. No numerical JBI cut-off or global MMAT score was used. Detailed item-level judgments are provided in Supplementary Table S7.

**Table 4:** Functional arrangement of the proposed layered model.

Functional domain or performance element	Health EDRM component(s)	Illustrative indicators	Functional rationale
System support	Policies, strategies and legislation; Financial resources	Mandates, regulation, governance, preparedness budgets, procurement authority, financing flexibility	Creates authorization and resources for center and district preparedness.
Core preparedness capacity	Planning and coordination; Human resources; Health infrastructure and logistics	Executable plans and SOPs, roles, training, staffing, team safety, utilities, WASH, essential stocks, transport and referral support	Organizes and sustains minimum center operations.
Service and community response	Health and related services; Community capacities for Health EDRM	Triage and basic emergency care, referral, surveillance, psychosocial care, outreach, vulnerable-population follow-up, community partnerships	Translates readiness into continued care, referral, outreach, access, and community partnership; community participation also informs planning, communication, implementation, monitoring, and evaluation.
Cross-cutting functions	Information and knowledge management; Risk communications; Monitoring and evaluation	Early warning, interoperable information, incident updates, communication with staff and communities, audits, exercises, response-time and service-continuity monitoring	Connects all domains and enables detection, communication, adaptation, accountability, and learning.
Overall objective	Continuity of Operations and Essential Services	Facility operational status, service downtime, workforce availability, essential-medicine availability or stock-outs, referral completion, restoration time, and uninterrupted access for priority populations	Defines measurable performance outcomes for assessment and interpretation.

Health EDRM, Health Emergency and Disaster Risk Management; SOPs, standard operating procedures; WASH, water, sanitation, and hygiene. The domains are functionally interdependent and should not be interpreted as sequential stages, levels of maturity, or a tested causal hierarchy. Community participation extends across the full preparedness cycle.

**Table 5:** Comparative occurrence of Health EDRM components, ordered by frequency in the 15 primary PHC studies.

Health EDRM component	Primary PHC *n*/15 (%)	Contextual n/59 (%)	All mapped *n*/74 (%)	Difference, percentage points	Descriptive interpretation
Human resources	14/15 (93.3%)	36/59 (61.0%)	50/74 (67.6%)	+32.3	Reported more frequently in PHC evidence
Planning and coordination	13/15 (86.7%)	30/59 (50.8%)	43/74 (58.1%)	+35.9	Reported more frequently in PHC evidence
Information and knowledge management	11/15 (73.3%)	23/59 (39.0%)	34/74 (45.9%)	+34.3	Reported more frequently in PHC evidence
Health infrastructure and logistics	10/15 (66.7%)	33/59 (55.9%)	43/74 (58.1%)	+10.8	Comparable reporting frequency across evidence sets
Monitoring and evaluation	10/15 (66.7%)	18/59 (30.5%)	28/74 (37.8%)	+36.2	Reported more frequently in PHC evidence
Health and related services	8/15 (53.3%)	23/59 (39.0%)	31/74 (41.9%)	+14.3	Reported with moderate frequency difference in PHC evidence
Policies, strategies and legislation	7/15 (46.7%)	17/59 (28.8%)	24/74 (32.4%)	+17.9	Reported with moderate frequency difference in PHC evidence
Community capacities for Health EDRM	6/15 (40.0%)	19/59 (32.2%)	25/74 (33.8%)	+7.8	Comparable reporting frequency across evidence sets
Financial resources	5/15 (33.3%)	9/59 (15.3%)	14/74 (18.9%)	+18.0	Reported with moderate frequency difference in PHC evidence
Risk communications	4/15 (26.7%)	13/59 (22.0%)	17/74 (23.0%)	+4.7	Comparable reporting frequency across evidence sets

Health EDRM, Health Emergency and Disaster Risk Management; PHC, primary healthcare center. Frequencies represent reporting occurrence, not causal importance. A publication could contribute to more than one component and was counted once within each component. The two evidence sets were assembled through different identification procedures; therefore, differences should be interpreted as descriptive signals for assessment design, not as evidence of inherent differences between PHC and non-PHC preparedness. The complete study-reference crosswalk, source-derived coding basis, and text-filled component matrix are provided in Supplementary Tables S8–S11.

### 3.2 Characteristics and quality of primary empirical PHC evidence

Across the primary PHC studies, methodological confidence varied by design and reporting quality. Litke et al. (15) provided the strongest qualitative audit trail, including transparent sampling, researcher positioning, reflexive safeguards, systematic analysis, and extensive participant quotations. Yari et al. (16) and Ndumbi et al. (17) used broad or structured facility samples and standardized preparedness criteria, although assessor reliability or residual confounding remained concerns. Derese et al. (18) included all 101 functioning public health centers in Addis Ababa and combined a standardized cholera-readiness checklist with key-informant interviews and focus groups; confidence was reduced by limited reporting of the qualitative analytic process, restricted measurement-validation evidence, an equally weighted binary preparedness threshold, and incomplete confounder specification. Lamberti-Castronuovo et al. (19) and Lapcevic et al. (20) provided operationally useful facility-assessment evidence but had substantial limitations for population-level generalization; the latter applied the Hospital Safety Index framework (21). Prakash et al. (22) was constrained by convenience snowball sampling, an unknown response rate, limited questionnaire validation, and unadjusted associations. Hashikawa and Gold (23) and Zarei et al. (24) contributed useful mixed or qualitative evidence but had small samples or incomplete reporting of reflexivity and source-to-theme traceability. These limitations informed interpretation but were not used as exclusion thresholds (see Table 3).

### 3.3 Comparative occurrence of health EDRM components

Human resources, planning and coordination, information and knowledge management, and monitoring and evaluation were reported more frequently in the primary PHC studies than in the contextual set, with percentage-point differences of 32.3, 35.9, 34.3, and 36.2, respectively (Table 5). Across PHC studies, these components converged around staff training and deployability, executable plans, surveillance and information flow, and repeated facility assessment. Infrastructure and logistics showed a smaller difference between sets (+10.8 percentage points), reflecting attention to utilities, WASH, equipment, transport, and supply continuity across facility and health-system settings.

Community capacities and risk communications were comparatively under-reported in both sets, particularly in the primary PHC studies (40.0 and 26.7%, respectively). Contextual publications added transferable detail concerning community engagement, vulnerable-population access, community-health-worker deployment, language access, and continuity of outreach, but they were not counted as direct PHC evidence. Financial resources were also infrequently reported in the primary PHC set. Publication-level coding and source-derived indicator phrases are provided in Supplementary Tables S8–S13. Collectively, these findings address RQ1 by identifying the most frequently reported preparedness capacities and operational gaps in the primary PHC evidence, particularly those concerning workforce readiness, executable planning and coordination, information flow, basic infrastructure and logistics, monitoring, community engagement, risk communication, and financing. They address RQ2 by showing that human resources, planning and coordination, information and knowledge management, and monitoring and evaluation were reported more frequently in the primary PHC studies than in the contextual set, whereas infrastructure and logistics, community capacities, and risk communications showed smaller descriptive differences. These comparisons represent reporting patterns rather than differences in causal importance or effectiveness.

### 3.4 Distinctive operational characteristics of PHCs

To address RQ3, the primary PHC findings were examined for operational characteristics that were particularly relevant to first-contact, community-based facilities. First, workforce readiness extended beyond training completion. Primary PHC evidence reported technical knowledge, simulation, teamwork, emotional intelligence, stress management, multidisciplinary staffing, employee resilience, organizational adaptation, leadership attitudes, and practical barriers to work attendance (15, 19, 23, 25). Across these studies, workforce readiness encompassed technical competence, teamwork, psychological readiness, prior experience, and barriers to deployment rather than training completion alone.

Second, plan usability was more prominent than plan existence alone. Primary PHC evidence identified inconsistent application of cholera protocols, written preparedness plans and coordination mechanisms, guideline usability and cultural adaptation, incident plans, referral and transport arrangements, patient transfer, and isolation procedures (17–19, 24). Across these studies, plan-related findings concerned usability, role clarity, activation, referral, transport, and external coordination.

Third, continuity depends on basic utilities, local stocks, water, sanitation, and hygiene (WASH) capacity. Primary PHC studies reported oral rehydration solution, intravenous fluids, antibiotics, safe water, latrines, waste disposal, handwashing facilities, and personal protective equipment (PPE) (18); PPE, stocks, and utilities (19); water, fuel, medicines, and equipment (26); WASH, chlorine, sterilization, waste, oral rehydration solution, and emergency fluids (17); and utilities, laboratory equipment, and 72-h operational capacity (20). Contextual evidence addressed emergency-water policy and infrastructure (6), WASH, transport, and hazard-exposed infrastructure (27), and backup power and flood-resilient healthcare infrastructure (28). Hurricane-response evidence documented disruptions involving power, water, communications, and critical supplies (29). Infection-prevention evidence addressed PPE, equipment, and facility readiness (30).

Fourth, information and monitoring formed an adaptive-intelligence function. Primary PHC studies reported early warning and continuous needs assessment (24), health information systems and repeated operational assessment (19), and disaster-preparedness questionnaire assessment (31). Facility-safety assessments included monitoring functions in Indonesia (26), Iran (16), and Serbia (20). Other PHC studies reported surveillance, warning, and evaluation processes (32, 33) and threat appraisal and exercises (23). Contextual evidence addressed multisectoral human-animal health data integration at primary level (34), machine-learning-supported airborne infection-risk modelling (35), geographic information systems and accessibility modelling (36), preparedness performance metrics (37), auditable threat assessment (38), and integrated information-technology architecture, data repositories, web-based coordination, and tracking functions (39). Together, these functions support repeated detection, assessment, action, and learning.

Fifth, community-facing continuity differentiated PHC preparedness from hospital readiness. Primary PHC evidence included collaboration with communities and other organizations (40) and service integration and community engagement (19). A study of PHC physicians assessed disaster mental-health knowledge, attitudes, and practices, including preparedness for psychological first aid and psychosocial needs, rather than observed delivery of mental-health services (22). Other PHC studies addressed essential services and community coordination (32, 33) and community-related facility-safety capacity (16). Contextual sources addressed provider roles for vulnerable populations (5), rural access and community needs (41), and public-private partnership-supported primary-care delivery (42). Continuity of noncommunicable-disease services was assessed in PHC networks in Malawi and Zambia (43). Rapid needs and community-resource assessment was examined during COVID-19 in the United States (44). Additional sources addressed community engagement for epidemic prevention and control (45), community health workers during pandemics (46), climate-related service continuity and community participation in African PHC (47), and service adaptation, telehealth, and community-resilience groups in European PHC (48).

### 3.5 Proposed layered model

To address RQ4, the findings from the primary PHC and contextual syntheses were organized into a PHC-oriented layered model linked to continuity of operations and essential services (Table 4; Figure 2). The synthesis supported a layered model with three nested functional domains and three cross-cutting functions. System Support includes policies, strategies and legislation and financial resources. Core Preparedness Capacity includes planning and coordination, human resources, and health infrastructure and logistics. Service and Community Response includes health and related services and community capacities. Information and knowledge management, risk communications, and monitoring and evaluation form Cross-Cutting Functions. The domains are functionally interdependent and should not be interpreted as sequential stages, levels of maturity, or a tested causal hierarchy. Although community capacities are grouped within Service and Community Response, community participation extends across risk assessment, planning, communication, implementation, monitoring, and evaluation. Continuity of Operations and Essential Services is the intended performance domain and can be assessed through operational status, service downtime, workforce and essential-medicine availability, referral completion, restoration time, and access for priority populations.

**Figure 2:**
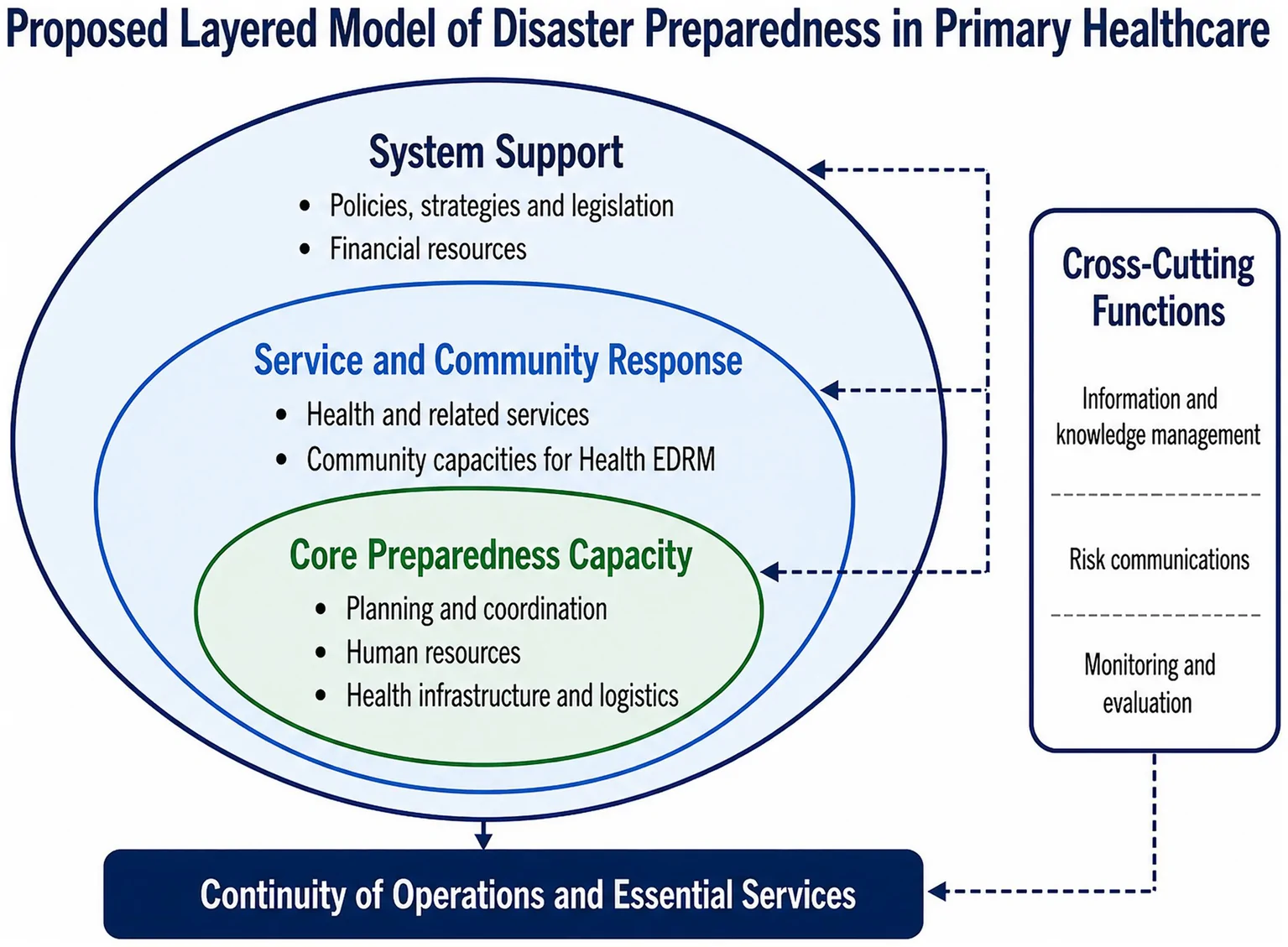
Proposed layered model of disaster preparedness in primary healthcare. System Support surrounds Service and Community Response and Core Preparedness Capacity. Information and knowledge management, risk communications, and monitoring and evaluation operate across all layers. Continuity of Operations and Essential Services is the overall objective. Dashed links are interpretive and do not represent tested causal effects. The domains are functionally interdependent and should not be interpreted as sequential stages, levels of maturity, or a tested causal hierarchy. In the figure, Health EDRM denotes Health Emergency and Disaster Risk Management.

## 4 Discussion

### 4.1 Principal findings

This review separated a focused systematic synthesis of 15 empirical PHC studies from a structured contextual evidence synthesis of 59 heterogeneous publications. Separation prevents secondary reviews, hospital evidence, policy analyses, and modelling studies from inflating the PHC denominator while still allowing transferable operational knowledge to be examined. The primary evidence consistently emphasized human resources, planning and coordination, information management, basic infrastructure and logistics, and monitoring. The nine publications added through the retrieval update and the one primary PHC study recovered through the Q1–Q4 filter audit increased the breadth of geographic and methodological coverage but did not change the central pattern: preparedness was not represented by a single plan, training program, or stockpile; it emerged as the ability of a small center to combine staff readiness, usable procedures, basic functional resources, information, referral, outreach, and continuous assessment.

The bibliographic, retrieval, and Q1–Q4 filter audits improved transparency and corrected identifiable errors, but they did not restore complete search reproducibility or eliminate the possibility of selection and retrieval bias. The recovery of one eligible PHC study from the 24 records affected by the journal-tier setting demonstrates that the pre-screening filter had a material effect on study inclusion. Reassessment of all 24 records corrected that specific omission, yet the original contemporaneous screening pathway cannot be recreated retrospectively. The retrieval update reduced the number of report records classified as not retrieved from 32 to 21. For those 21 records, bibliographic and publisher records were identifiable, but full-text copies could not be secured and locally verified through the legitimate access options available to the review team at that stage, including subscription- or institution-restricted access. The original Watase output is retained as an archived record of the original process, whereas the reconstructed PRISMA figure reports the corrected audited evidence base. Both should be read together with the limitations of retrospective reconstruction (see Figure 1).

### 4.2 Why PHC preparedness differs from hospital preparedness

The reviewed PHC evidence suggests that the operational emphasis of preparedness in PHC settings may differ from that commonly emphasized in hospital-oriented frameworks. This comparison is interpretive because the review did not systematically compare PHC and hospital preparedness. PHC evidence emphasized small generalist teams, minimum functional services, basic utilities, referral dependence, public-health functions, and close interaction with defined communities, whereas hospital-oriented sources more often addressed specialized surge and departmental capacity. Hospital-based assessments have examined disaster preparedness planning in hub hospitals in Nepal (83) and emergency-department preparedness in Italian hospitals (84). Hospital-derived functions may be relevant at referral level but are not sufficient proxies for PHC readiness.

A further operational feature was PHC dependence on interorganizational coordination. Primary PHC evidence identified coordination mechanisms and referral and transport arrangements (18, 19), while contextual evidence described external governance and service-delivery arrangements (42). These findings support attention to clear roles, activation procedures, communication responsibilities, and functional referral and transport pathways. The appropriate mechanism for formalizing such arrangements should be determined locally and evaluated in future implementation research.

### 4.3 Interpretation and contribution of the proposed layered model

The model does not introduce new preparedness components; it reorganizes the 10 Health EDRM components into PHC-oriented functional domains linked to continuity outcomes. This organization complements the Sendai, WHO Health EDRM, and Emergency Ready Primary Health Care frameworks by making explicit how system support, center-level capacity, service and community functions, and cross-cutting information, communication, and evaluation interact in PHC settings (1, 2, 49). The model is neither a staged maturity framework nor a tested causal hierarchy. Its contribution is an explicit evidence-to-domain pathway focused on minimum operational continuity, referral dependence, and community-facing functions.

The relationships among domains are reciprocal: governance and financing may constrain facility operations, while facility experience, community knowledge, monitoring results, and service interruptions may inform planning, allocation, and policy revision. Community participation therefore extends across assessment, planning, communication, implementation, monitoring, and evaluation rather than being confined to response delivery.

The use of the Hospital Safety Index in primary-care facility studies further underscores a field-level gap. Italian and Indonesian studies applied hospital-derived safety assessment approaches to PHC settings (19, 26). Similar applications were reported in Iran and Serbia (16, 20). A hospital-derived instrument is therefore among the most frequently used available approaches for PHC preparedness assessment. The proposed layered model may serve as scaffolding for developing a PHC-specific assessment instrument that captures the community, service-continuity, and cross-cutting information functions that hospital-derived tools underweight.

### 4.4 Implications and contextual adaptation

For assessment development, plan availability and training counts should be supplemented by measures of plan usability, staff deployability and personal readiness, utilities and WASH, local stocks, referral functionality, incident information, risk communication, community outreach, and service interruption. This approach can identify which interacting function constrains continuity rather than merely enumerate resource deficiencies.

Implementation is likely to vary across national and subnational health systems according to governance arrangements, the degree of decentralization, PHC financing and fiscal space, workforce density and distribution, referral infrastructure, rurality and remoteness, conflict or displacement exposure, digital connectivity, supply-chain reliability, and the predominant local hazard profile. The layered model should therefore be operationalized as a context-adaptable assessment framework rather than a uniform package. Potential continuity indicators include facility opening and operational status, duration of service interruption, workforce availability, essential-medicine availability and stock-outs, referral completion, restoration time, and uninterrupted access for priority populations.

Operationalization should remain context adaptable because governance, financing, workforce, referral infrastructure, remoteness, conflict exposure, digital connectivity, supply-chain reliability, and hazard profiles differ across health systems. Resource-constrained settings may prioritize executable plans, minimum staffing, staff safety, essential medicines, PPE, WASH, basic communications, referral pathways, and outreach arrangements, while better-resourced systems may add interoperable information, infrastructure redundancy, predictive analytics, and telehealth.

These differences represent feasible sequencing of investment rather than a ranking of preparedness value or system maturity. Potential continuity indicators include facility operational status, duration of service interruption, workforce and essential-medicine availability, referral completion, restoration time, and uninterrupted access for priority populations.

Because PHC readiness partly depends on organizations outside the facility, assessment should examine whether roles, activation procedures, communication responsibilities, referral pathways, and transport arrangements are clear and functional. The appropriate mechanism should be determined and evaluated locally.

### 4.5 Strengths and limitations

The primary Scopus search was supported by a preserved strategy and record-level audit trail. The available contemporaneous records supported the principal search and selection counts for the supplementary pathway; however, the level of documentation varied across the supplementary databases. The intermediate screening stages for PubMed and OpenAlex were documented less comprehensively than those for ProQuest. Accordingly, the supplementary pathway should be interpreted as a targeted evidence supplement rather than as a set of fully reproducible parallel systematic searches, and selection bias cannot be excluded. The primary evidence base was geographically uneven and dominated by cross-sectional, descriptive, and single-facility studies, limiting generalizability and precluding causal inference. Component occurrence reflects reporting prominence rather than demonstrated influence or effectiveness. English-language eligibility, reliance on Scopus as the primary database, and the absence of dedicated regional databases may also have underrepresented other-language literature. The contextual set was used only for descriptive triangulation and model interpretation and did not receive the same item-level appraisal as the primary PHC studies.

The review was conducted under a predefined protocol and was registered retrospectively on the OSF after screening, extraction, and initial synthesis. To mitigate *post-hoc* selection and reporting bias, all final eligibility, retrieval, appraisal, coding, and synthesis decisions were rechecked against the prespecified criteria, independently confirmed by two reviewers, and documented in the time-stamped OSF amendment and consensus records. The proposed layered model was developed through synthesis and interpretation of the reviewed evidence and was internally reviewed through the two-reviewer consensus process described in Section 2.10 but has not been externally or empirically validated. Formal validation should include multidisciplinary Delphi consultation, content validity assessment, and prospective testing across diverse PHC systems.

### 4.6 Research priorities

The 15-study primary empirical set also warrants expansion through multicenter longitudinal studies. Future reviews should preserve report-level retrieval reasons prospectively, maintain dual-reviewer documentation for all late retrieval and filter-audit decisions, and distinguish center-level PHC evidence from workforce-only, hospital, and health-system evidence. Beyond the validation steps described in Section 4.5, future work should test the layered model against real-world PHC preparedness data collected from diverse hazard and health-system contexts.

## 5 Conclusion

This review addressed four related questions concerning PHC disaster preparedness and service continuity. First, the primary PHC evidence identified workforce readiness, executable planning and coordination, information flow, basic infrastructure and logistics, monitoring, referral functionality, and community-facing services as central preparedness capacities, while community capacities, risk communication, and financing remained comparatively under-reported operational areas. Second, comparison with the contextual evidence set showed that human resources, planning and coordination, information and knowledge management, and monitoring and evaluation were reported more frequently in primary PHC studies, although these differences indicate reporting patterns rather than causal importance. Third, operationally distinctive PHC characteristics included small generalist teams, staff and family-readiness barriers, dependence on basic utilities and WASH, limited local stocks, referral dependence, community outreach, and repeated facility-level monitoring. Fourth, these findings were organized into a layered model comprising System Support, Core Preparedness Capacity, Service and Community Response, and Cross-Cutting Functions, linked to continuity of operations and essential services. The domains are functionally interdependent and should not be interpreted as sequential stages, levels of maturity, or a tested causal hierarchy. Implementation and future validation should account for differences in governance, financing, workforce, referral infrastructure, digital capacity, humanitarian conditions, and local hazard profiles.

## Data Availability

The original contributions presented in the study are included in the article/Supplementary material, further inquiries can be directed to the corresponding author.
