# Supplementary material for: Disaster preparedness and service continuity in primary healthcare centers: a systematic review and structured contextual evidence synthesis
Source: Front Public Health. 2026 Oct 28;14:1961009. doi: 10.3389/fpubh.2026.1961009 (PMC13752140; doi:10.3389/fpubh.2026.1961009)
Supplement: Supplementary file 1 [file Table_1.docx]

**PRISMA 2020 Checklist – Completed**

Manuscript: Disaster preparedness and service continuity in primary healthcare centers: a systematic review and structured contextual evidence synthesis

*Page references are to the main manuscript unless identified as Supplementary. “Not applicable” indicates that no meta-analysis or outcome-level statistical synthesis was undertaken.*

| **Section and Topic** | **Item #** | **Checklist item** | **Location where item is reported** |
| --- | --- | --- | --- |
| **TITLE** | | |  |
| **Title** | **1** | Identify the report as a systematic review. | p. 1, title. |
| **ABSTRACT** | | |  |
| **Abstract** | **2** | See the PRISMA 2020 for Abstracts checklist. | p. 1, Abstract. |
| **INTRODUCTION** | | |  |
| **Rationale** | **3** | Describe the rationale for the review in the context of existing knowledge. | pp. 1-2, Introduction. |
| **Objectives** | **4** | Provide an explicit statement of the objective(s) or question(s) the review addresses. | p. 2, final paragraph of Introduction (objective and four review questions). |
| **METHODS** | | |  |
| **Eligibility criteria** | **5** | Specify the inclusion and exclusion criteria for the review and how studies were grouped for the syntheses. | p. 4, Sections 2.5–2.6; Supplementary Table S3. |
| **Information sources** | **6** | Specify all databases, registers, websites, organisations, reference lists and other sources searched or consulted to identify studies. Specify the date when each source was last searched or consulted. | p. 3, Section 2.4; Supplementary Table S1, p. 1. PubMed was searched on 27 June 2026; Scopus, ProQuest, and OpenAlex were searched on 30 June 2026. |
| **Search strategy** | **7** | Present the full search strategies for all databases, registers and websites, including any filters and limits used. | p. 3, Section 2.4; complete database-specific strategies, filters, and limits in Supplementary Table S1, p. 1. |
| **Selection process** | **8** | Specify the methods used to decide whether a study met the inclusion criteria of the review, including how many reviewers screened each record and each report retrieved, whether they worked independently, and if applicable, details of automation tools used in the process. | p. 4, Section 2.6; p. 5, Section 2.9; Supplementary Tables S2–S3. |
| **Data collection process** | **9** | Specify the methods used to collect data from reports, including how many reviewers collected data from each report, whether they worked independently, any processes for obtaining or confirming data from study investigators, and if applicable, details of automation tools used in the process. | pp. 4–5, Sections 2.6–2.7; standardized extraction matrix and independent two-reviewer verification; Supplementary Table S5. |
| **Data items** | **10a** | List and define all outcomes for which data were sought. Specify whether all results that were compatible with each outcome domain in each study were sought (e.g. for all measures, time points, analyses), and if not, the methods used to decide which results to collect. | p. 3, Section 2.3; p. 5, Section 2.7. |
|  | **10b** | List and define all other variables for which data were sought (e.g. participant and intervention characteristics, funding sources). Describe any assumptions made about any missing or unclear information. | p. 5, Section 2.7; Supplementary Table S5. |
| **Study risk of bias assessment** | **11** | Specify the methods used to assess risk of bias in the included studies, including details of the tool(s) used, how many reviewers assessed each study and whether they worked independently, and if applicable, details of automation tools used in the process. | p. 5, Section 2.8; Supplementary Tables S6–S7. |
| **Effect measures** | **12** | Specify for each outcome the effect measure(s) (e.g. risk ratio, mean difference) used in the synthesis or presentation of results. | p. 5, Section 2.7; descriptive measures were counts, percentages, and percentage-point differences. No pooled effect measure was used. |
| **Synthesis methods** | **13a** | Describe the processes used to decide which studies were eligible for each synthesis (e.g. tabulating the study intervention characteristics and comparing against the planned groups for each synthesis (item #5)). | pp. 4–6, Sections 2.5, 2.7, and 2.10. |
|  | **13b** | Describe any methods required to prepare the data for presentation or synthesis, such as handling of missing summary statistics, or data conversions. | pp. 4–5, Sections 2.6–2.7; Supplementary Tables S4–S5. No conversion of summary statistics was required. |
|  | **13c** | Describe any methods used to tabulate or visually display results of individual studies and syntheses. | pp. 5–6, Sections 2.7 and 2.10; Figures 1–2, pp. 11–12; Tables 1–5, pp. 23–29; Supplementary Tables S5–S14. |
|  | **13d** | Describe any methods used to synthesize results and provide a rationale for the choice(s). If meta-analysis was performed, describe the model(s), method(s) to identify the presence and extent of statistical heterogeneity, and software package(s) used. | p. 2, Section 2.1; pp. 5–6, Sections 2.7 and 2.10. SWiM-informed descriptive synthesis; meta-analysis was not performed because of substantial heterogeneity. |
|  | **13e** | Describe any methods used to explore possible causes of heterogeneity among study results (e.g. subgroup analysis, meta-regression). | Not applicable - no subgroup analysis, meta-regression, or other statistical investigation of heterogeneity was conducted. |
|  | **13f** | Describe any sensitivity analyses conducted to assess robustness of the synthesized results. | Not applicable - no review-level sensitivity analysis was conducted. |
| **Reporting bias assessment** | **14** | Describe any methods used to assess risk of bias due to missing results in a synthesis (arising from reporting biases). | Not applicable – no effect-estimate synthesis was performed. Potential retrieval and selection biases are discussed on p. 15, Section 4.7. |
| **Certainty assessment** | **15** | Describe any methods used to assess certainty (or confidence) in the body of evidence for an outcome. | Not applicable – no outcome-level certainty framework (e.g., GRADE) was applied. Study-level methodological appraisal is reported on p. 5, Section 2.8, and Supplementary Tables S6–S7. |
| **RESULTS** | | |  |
| **Study selection** | **16a** | Describe the results of the search and selection process, from the number of records identified in the search to the number of studies included in the review, ideally using a flow diagram. | p. 6, Section 3.1; Figure 1, p. 11; Table 1, p. 23; Supplementary Tables S2–S4. |
|  | **16b** | Cite studies that might appear to meet the inclusion criteria, but which were excluded, and explain why they were excluded. | p. 6, Section 3.1; record-specific exclusions in Supplementary Tables S2–S3. |
| **Study characteristics** | **17** | Cite each included study and present its characteristics. | p. 6, Section 3.1; Table 2, pp. 24–25; Supplementary Table S5. |
| **Risk of bias in studies** | **18** | Present assessments of risk of bias for each included study. | p. 7, Section 3.2; Table 5, pp. 28–29; Supplementary Tables S6–S7. |
| **Results of individual studies** | **19** | For all outcomes, present, for each study: (a) summary statistics for each group (where appropriate) and (b) an effect estimate and its precision (e.g. confidence/credible interval), ideally using structured tables or plots. | Adapted to the descriptive synthesis: study-level findings are presented in Table 2, pp. 24–25, and Supplementary Table S5. Pooled effect estimates were not applicable. |
| **Results of syntheses** | **20a** | For each synthesis, briefly summarise the characteristics and risk of bias among contributing studies. | pp. 7–10, Sections 3.2–3.5; Tables 2, 3, and 5, pp. 24–29; Supplementary Tables S5–S14. |
|  | **20b** | Present results of all statistical syntheses conducted. If meta-analysis was done, present for each the summary estimate and its precision (e.g. confidence/credible interval) and measures of statistical heterogeneity. If comparing groups, describe the direction of the effect. | p. 7, Section 3.3; Table 3, p. 26. Descriptive counts, percentages, and percentage-point differences are reported; no meta-analysis was conducted. |
|  | **20c** | Present results of all investigations of possible causes of heterogeneity among study results. | Not applicable - no statistical investigation of heterogeneity was conducted. |
|  | **20d** | Present results of all sensitivity analyses conducted to assess the robustness of the synthesized results. | Not applicable - no review-level sensitivity analysis was conducted. |
| **Reporting biases** | **21** | Present assessments of risk of bias due to missing results (arising from reporting biases) for each synthesis assessed. | Not applicable – formal reporting-bias assessment was not undertaken because no effect-estimate synthesis was performed; related limitations are discussed on p. 15, Section 4.7. |
| **Certainty of evidence** | **22** | Present assessments of certainty (or confidence) in the body of evidence for each outcome assessed. | Not applicable – no outcome-level certainty assessment was undertaken. Narrative methodological confidence is reported on p. 7, Section 3.2, Table 5, and Supplementary Tables S6–S7. |
| **DISCUSSION** | | |  |
| **Discussion** | **23a** | Provide a general interpretation of the results in the context of other evidence. | pp. 12–14, Sections 4.1–4.4. |
|  | **23b** | Discuss any limitations of the evidence included in the review. | p. 7, Section 3.2; pp. 15–16, Section 4.7. |
|  | **23c** | Discuss any limitations of the review processes used. | pp. 15–16, Section 4.7. |
|  | **23d** | Discuss implications of the results for practice, policy, and future research. | pp. 14–16, Sections 4.5–4.8. |
| **OTHER INFORMATION** | | |  |
| **Registration and protocol** | **24a** | Provide registration information for the review, including register name and registration number, or state that the review was not registered. | p. 3, Section 2.2. Retrospective OSF registration: https://doi.org/10.17605/OSF.IO/H8JU7. Associated project: https://osf.io/7atku/ |
|  | **24b** | Indicate where the review protocol can be accessed, or state that a protocol was not prepared. | p. 3, Section 2.2; p. 16, Data availability statement; retrospective OSF registration at https://doi.org/10.17605/OSF.IO/H8JU7. |
|  | **24c** | Describe and explain any amendments to information provided at registration or in the protocol. | p. 3, Section 2.2; p. 4, Section 2.6; p. 5, Section 2.9; amendment log deposited in OSF. |
| **Support** | **25** | Describe sources of financial or non-financial support for the review, and the role of the funders or sponsors in the review. | p. 17, Funding and Acknowledgments. |
| **Competing interests** | **26** | Declare any competing interests of review authors. | p. 17, Conflict of interest. |
| **Availability of data, code and other materials** | **27** | Report which of the following are publicly available and where they can be found: template data collection forms; data extracted from included studies; data used for all analyses; analytic code; any other materials used in the review. | p. 16, Data availability statement; Supplementary Tables S1–S14; retrospective OSF registration at https://doi.org/10.17605/OSF.IO/H8JU7. |

*From:* Page MJ, McKenzie JE, Bossuyt PM, Boutron I, Hoffmann TC, Mulrow CD, et al. The PRISMA 2020 statement: an updated guideline for reporting systematic reviews. BMJ 2021;372:n71. doi: 10.1136/bmj.n71. This work is licensed under CC BY 4.0. To view a copy of this license, visit <https://creativecommons.org/licenses/by/4.0/>

*Checklist source: Official PRISMA 2020 checklist (Page et al., 2021), completed for this manuscript. The flow diagram is supplied as a separate official-template-adapted figure. The checklist is distributed under CC BY 4.0.*
