# Supplementary material for: Disaster preparedness and service continuity in primary healthcare centers: a systematic review and structured contextual evidence synthesis
Source: Front Public Health. 2026 Oct 28;14:1961009. doi: 10.3389/fpubh.2026.1961009 (PMC13752140; doi:10.3389/fpubh.2026.1961009)
Supplement: Supplementary file 2 [file Table_2.docx]

Supplementary Material

# Audit scope and coding conventions

This file consolidates Supplementary Tables S1–S14 using the verified search, retrieval, Q1–Q4 filter-audit, evidence-role, appraisal, Health EDRM coding, frequency, and citation-traceability material supporting the synchronized manuscript. The final evidence base contains 74 unique publications: 15 primary empirical PHC studies and 59 contextual publications. Y indicates that a source-derived indicator was identified; NR indicates that no qualifying indicator was identified in the retained text and must not be interpreted as evidence that the component was absent in the study setting. All primary-study eligibility, appraisal, and coding decisions were independently confirmed by two reviewers. This synchronized version incorporates verified reference substitutions and claim-level corrections reflected in the audited main manuscript; it does not create a new study finding, evidence group, or exclusion decision.

# Supplementary Table S1. Database-specific search strategies and source provenance

| **Database/source** | **Field or coverage** | **Complete search string or procedure** | **Limits** | **Audit note** |
| --- | --- | --- | --- | --- |
| Scopus | TITLE-ABS-KEY | TITLE-ABS-KEY(("disaster preparedness" OR "emergency preparedness" OR "emergency planning" OR "health emergency" OR "disaster readiness" OR "facility preparedness" OR "facility readiness" OR resilience OR "continuity of essential services") AND ("primary healthcare" OR "primary health care" OR "primary health center" OR "primary health centre" OR "primary healthcare center" OR "primary healthcare centre" OR "community health center" OR "community health centre" OR "community clinic" OR "family health center" OR "public health center" OR puskesmas OR "health facility" OR "healthcare facility")) | Publication years 2016–2026; Watase Q1–Q4 journal-tier setting applied before eligibility screening; searched 30 June 2026 | Primary export contained 201 records. Twenty-four records were removed by the historical Q1–Q4 tier setting before eligibility screening. The final audit reconstructed all 24 identities and reassessed them against the prespecified criteria: one primary empirical PHC study was included and 23 records were excluded. No final eligibility decision was based solely on journal quartile. |
| PubMed | Title/Abstract and MeSH-supported search | (("disaster preparedness"[Title/Abstract] OR "emergency preparedness"[Title/Abstract] OR "emergency planning"[Title/Abstract] OR "health emergency"[Title/Abstract] OR "disaster readiness"[Title/Abstract] OR "facility preparedness"[Title/Abstract] OR "facility readiness"[Title/Abstract] OR resilience[Title/Abstract] OR "continuity of essential services"[Title/Abstract] OR "Disaster Planning"[MeSH Terms] OR "Emergencies"[MeSH Terms]) AND ("primary healthcare"[Title/Abstract] OR "primary health care"[Title/Abstract] OR "primary health center"[Title/Abstract] OR "primary health centre"[Title/Abstract] OR "primary healthcare center"[Title/Abstract] OR "primary healthcare centre"[Title/Abstract] OR "community health center"[Title/Abstract] OR "community health centre"[Title/Abstract] OR "community clinic"[Title/Abstract] OR "family health center"[Title/Abstract] OR "public health center"[Title/Abstract] OR puskesmas[Title/Abstract] OR "health facility"[Title/Abstract] OR "healthcare facility"[Title/Abstract] OR "Primary Health Care"[MeSH Terms])) | English; full-text peer-reviewed articles; 2016 to June 2026; searched 27 June 2026 | Preserved supplementary set: 10 selected publications; retained source-specific screening yield, not reconstructable as an unfiltered database-hit count. |
| ProQuest | Title, abstract, and subject fields | ab("disaster preparedness" OR "emergency preparedness" OR "emergency planning" OR "health emergency" OR "disaster readiness" OR "facility preparedness" OR "facility readiness" OR resilience OR "continuity of essential services") AND ab("primary healthcare" OR "primary health care" OR "primary health center" OR "primary health centre" OR "primary healthcare center" OR "primary healthcare centre" OR "community health center" OR "community health centre" OR "community clinic" OR "family health center" OR "public health center" OR puskesmas OR "health facility" OR "healthcare facility") | English; scholarly/peer-reviewed journals; full text; 2016 to June 2026; searched 30 June 2026 | Preserved supplementary set: 10 selected publications; retained source-specific screening yield, not reconstructable as an unfiltered database-hit count. |
| OpenAlex | Keyword- and concept-based bibliographic search | ("disaster preparedness" OR "emergency preparedness" OR "emergency planning" OR "health emergency" OR "disaster readiness" OR "facility preparedness" OR "facility readiness" OR resilience OR "continuity of essential services") AND ("primary healthcare" OR "primary health care" OR "primary health center" OR "primary health centre" OR "primary healthcare center" OR "primary healthcare centre" OR "community health center" OR "community health centre" OR "community clinic" OR "family health center" OR "public health center" OR puskesmas OR "health facility" OR "healthcare facility") | 2016 to June 2026; English-language peer-reviewed records prioritized; searched 30 June 2026 | Preserved supplementary set: 6 selected publications; retained source-specific screening yield, not reconstructable as an unfiltered database-hit count. |
| Additional sources | Backward, forward, and manual screening | Reference lists of eligible and highly relevant studies were screened for PHC-relevant or transferable preparedness evidence. | Peer-reviewed full-text publications relevant to preparedness determinants | Used to reduce cross-disciplinary indexing omissions. |

Search-date boundary: the PubMed search was conducted on 27 June 2026, and the Scopus, ProQuest, and OpenAlex searches were conducted on 30 June 2026. A targeted pre-submission full-text retrieval and bibliographic update was completed in July 2026. Database-specific execution dates were therefore available for reporting. In the historical Scopus workflow, the Watase record limitation was configured for publication years 2016–2026 and journal tiers Q1–Q4; all 24 affected records were subsequently reconstructed and reassessed against the prespecified eligibility criteria.

# Supplementary Table S2. Retrieval audit of the 32 reports initially recorded as not retrieved

The original Watase retrieval screen identified report records 43–74 as not retrieved. During the pre-submission update, full-text copies were obtained and locally verified for 11 reports. For the other 21 report records, bibliographic and publisher records were identified, but full-text copies could not be secured for local verification through the legitimate access options available to the review team at that stage, including subscription- or institution-restricted access. One of these report identities (Pawar et al.) was already represented in the final evidence base through a separately obtained supplementary-source copy and did not add a publication to the final count. Full-text eligibility assessment could not be completed for the other 20 report identities. The designation “reports not retrieved” therefore describes the historical Scopus full-text retrieval pathway and should not be interpreted as indicating that the publications did not exist or could not be identified bibliographically. Report-specific reasons for the original non-retrieval were not recorded contemporaneously and were not assigned retrospectively.

| **Retrieval item** | **n** |
| --- | --- |
| Initially not retrieved | 32 |
| Accessible full texts subsequently obtained | 11 |
| Reports not retrieved in the Scopus pathway | 21 |
| Erratum/non-substantive file | 1 |
| Substantive reports reassessed | 10 |
| Excluded after reassessment | 1 |
| Additional eligible publications | 9 |

| **Watase no.** | **Author/year** | **Short title** | **DOI/identifier as recorded** | **Current retrieval status** | **Review decision** |
| --- | --- | --- | --- | --- | --- |
| 43 | Andjar et al. (2026) | A weighted-index assessment of community disaster preparedness in Yogyakarta, Indonesia | 10.1080/17477891.2026.2617615 | Full-text copy not retrieved for local verification | Not assessed – full text not retrieved |
| 44 | Deelstra and Bristow (2023) | Methods for representing regional disaster recovery estimates: modelling approaches and assessment tools for improving emergency planning and preparedness | 10.1007/s11069-023-05883-5 | Full-text copy not retrieved for local verification | Not assessed – full text not retrieved |
| 45 | Van Hoang Minh et al. (2021) | COVID-19 preparedness and response capability: a case study of the Hanoi primary healthcare system | 10.1177/11786329211019224 | Retrieved in pre-submission update | Included - primary PHC |
| 46 | Wang and Sun (2025) | Optimizing the layout of primary healthcare facilities in Harbin's main urban area, China: a resilience perspective | 10.3390/su17198706 | Full-text copy not retrieved for local verification | Not assessed – full text not retrieved |
| 47 | Mussa Kwikima (2026) | Community and hospital preparedness in Dodoma Municipality, Tanzania: a dual assessment of household preparedness and health-facility emergency readiness | Not visible in Watase capture | Full-text copy not retrieved for local verification | Not assessed – full text not retrieved |
| 48 | Shojaei et al. (2024) | Comparative analysis of two disaster risk assessment tools for primary health care centers: the case of Iran | 10.1016/j.ijdrr.2024.104466 | Full-text copy not retrieved for local verification | Not assessed – full text not retrieved |
| 49 | Niazi et al. (2021) | Seismic resilience index evaluation for healthcare facilities: a case study of a hospital in Tehran | 10.1016/j.ijdrr.2021.102639 | Full-text copy not retrieved for local verification | Not assessed – full text not retrieved |
| 50 | Gundran et al. (2023) | Simulation training needs assessment for disaster preparedness and disaster response among selected agencies in the National Capital Region, Philippines | 10.1016/j.ijdrr.2023.103824 | Full-text copy not retrieved for local verification | Not assessed – full text not retrieved |
| 51 | Alyaseen et al. (2024) | Assessment of disaster preparedness for mass casualty incidents: a perspective from Saudi healthcare workers | 10.1016/j.ijdrr.2024.104300 | Full-text copy not retrieved for local verification | Not assessed – full text not retrieved |
| 52 | Patel et al. (2025) | Assessment of internal disaster and emergency preparedness in hospitals using the Hospital Safety Index tool | 10.1016/j.mjafi.2024.08.011 | Full-text copy not retrieved for local verification | Not assessed – full text not retrieved |
| 53 | Pawar et al. (2024) | Domain-specific need assessment for hospital disaster preparedness: a systematic review and critical interpretative synthesis | 10.1016/j.ijdrr.2024.104437 | Full-text copy not retrieved through this Scopus pathway; separately obtained copy represented in final evidence base | Included – contextual; duplicate source identity, no additional publication count |
| 54 | Wilkins et al. (2025) | The regional impact of COVID-19 on U.S. academic medical centers: lessons for public health emergency preparedness and response | 10.1017/dmp.2025.10217 | Full-text copy not retrieved for local verification | Not assessed – full text not retrieved |
| 55 | Alrayyes et al. (2023) | The emergency preparedness of level-four primary health care centers in the Gaza Strip, the occupied Palestinian territory | 10.1017/S1049023X22002357 | Full-text copy not retrieved for local verification | Not assessed – full text not retrieved |
| 56 | Kuppanda et al. (2023) | An assessment of pediatric resident disaster preparedness for the neonatal intensive care unit | 10.1017/dmp.2021.322 | Full-text copy not retrieved for local verification | Not assessed – full text not retrieved |
| 57 | Martinez et al. (2020) | The 2017 Hurricane public health responses - Erratum | 10.1017/dmp.2020.181 | Retrieved | Excluded - erratum/non-substantive |
| 58 | Sharifian et al. (2017) | Assessment of disaster mitigation and preparedness | 10.5812/traumamon.41082 | Retrieved | Included - contextual |
| 59 | Rahmati-Najarkolaei et al. (2017) | Assessment of a military hospital's disaster preparedness using a Health Incident Command System | 10.5812/traumamon.31448 | Retrieved | Included - contextual |
| 60 | Khairina et al. (2023) | Disaster preparedness assessment in emergency department: a cross-sectional study | 10.5603/DEMJ.a2023.0005 | Full-text copy not retrieved for local verification | Not assessed – full text not retrieved |
| 61 | Khademipour and Sheikhbardsiri (2022) | Disaster risk assessment of primary healthcare facilities in South East of Iran | 10.5603/DEMJ.a2022.0002 | Full-text copy not retrieved for local verification | Not assessed – full text not retrieved |
| 62 | Vick et al. (2018) | Assessment of community hospital disaster preparedness in New York State | 10.5055/jem.2018.0371 | Full-text copy not retrieved for local verification | Not assessed – full text not retrieved |
| 63 | Peck et al. (2016) | Disaster behavioral health capacity: findings from a multistate preparedness assessment | 10.5055/jem.2016.0293 | Full-text copy not retrieved for local verification | Not assessed – full text not retrieved |
| 64 | Theron and Smith (2026) | Emergency preparedness and disaster management for primary health care in Africa from the perspective of climate hazards and extreme weather events | 10.4102/phcfm.v18i1.5449 | Retrieved | Included - contextual |
| 65 | Mohapatra and Mandal (2023) | Resilience measuring tools for risk assessment and disaster preparedness in India: the current state | 10.61275/ISVSej-2023-10-10-02 | Full-text copy not retrieved for local verification | Not assessed – full text not retrieved |
| 66 | Adhikari et al. (2025) | Assessment of disaster preparedness planning in 25 hub hospitals of Nepal | 10.33314/jnhrc.v23i02.4703 | Retrieved | Included - contextual |
| 67 | Dg Sirua et al. (2025) | The impact of nurses' disaster preparedness competencies on public health center resilience in disaster-prone areas | 10.24083/apjhm.v20i1.3973 | Retrieved | Included - primary PHC |
| 68 | Isangula et al. (2023) | Nurses' preparedness for disaster response in rural and urban primary healthcare settings in Tanzania | 10.22605/RRH7547 | Full-text copy not retrieved for local verification | Not assessed – full text not retrieved |
| 69 | Kurniawan et al. (2021) | How far disaster management implemented toward flood preparedness: a lesson from youth participation assessment in Indonesia | 10.18280/ijsse.110206 | Retrieved | Excluded - no healthcare/PHC context |
| 70 | Firdausi et al. (2021) | Disaster preparedness analysis of public health centers in DKI Jakarta Province in 2020 | 10.18280/ijsse.110110 | Retrieved | Included - primary PHC |
| 71 | Petrazzuoli et al. (2025) | Resilience of primary healthcare facilities: experiences from 16 European countries during the COVID-19 pandemic | 10.17219/acem/194212 | Retrieved | Included - contextual |
| 72 | Sutrisno et al. (2025) | Community preparedness for earthquake disasters: a preliminary assessment of awareness and disaster infrastructure response in Cianjur, West Java, Indonesia | 10.12688/f1000research.143577.3 | Full-text copy not retrieved for local verification | Not assessed – full text not retrieved |
| 73 | Paganini et al. (2016) | Assessment of disaster preparedness among emergency departments in Italian hospitals | 10.1186/s13049-016-0292-6 | Retrieved | Included - contextual |
| 74 | Quinn et al. (2024) | Assessment of a virtual disaster preparedness and response simulation compared with previous in-person iterations | 10.1080/13561820.2023.2249941 | Full-text copy not retrieved for local verification | Not assessed – full text not retrieved |

# Supplementary Table S3. Reassessment audit of the 24 records affected by the historical Q1–Q4 journal-tier setting

All 24 record identities were reconstructed from the complete Scopus/Watase export and reassessed against the prespecified population, setting, outcome, and design criteria. One record was included in the primary PHC synthesis and 23 were excluded. The included study was independently appraised and mapped to the Health EDRM framework.

| **Watase no.** | **Title** | **Year** | **Final decision** | **Eligibility reason** | **Audit note** |
| --- | --- | --- | --- | --- | --- |
| 6 | From rooms to intelligence: reclaiming preparedness in Africa's public health emergency operations centres | 2026 | Exclude | Ineligible setting | Public health emergency operations centres, not PHC facilities or PHC personnel. |
| 7 | Infrastructure to Culture: qualitative case study of Hospital Disaster Preparedness using Hospital Safety Index | 2026 | Exclude | Hospital-only setting | Type B teaching hospital; not an eligible PHC setting. |
| 10 | National resilience: validation of a four-dimensional disaster preparedness assessment model | 2026 | Exclude | Ineligible setting/unit of analysis | National resilience model development; no eligible PHC setting or PHC workforce. |
| 13 | The Davos Alzheimer's Collaborative Healthcare System Preparedness in primary care: Methodology | 2026 | Exclude | Ineligible outcome | Preparedness for cognitive screening implementation, not disaster or emergency preparedness. |
| 17 | Assessment of students' belief in disaster preparedness | 2025 | Exclude | Ineligible population | University students; no PHC setting or PHC workforce. |
| 19 | Breaking the Dust Barrier: preparedness of PHCs and staff knowledge on silicosis management | 2025 | Exclude | Ineligible outcome | Silicosis management is a chronic occupational-health topic rather than disaster/emergency preparedness under the review criteria. |
| 22 | Community-based disaster preparedness: a training program based on needs assessment | 2025 | Exclude | Ineligible population/setting | Community training participants; no eligible PHC facility or PHC workforce unit of analysis. |
| 25 | Disaster response system dynamic assessment model for nuclear accident preparedness education | 2025 | Exclude | Ineligible setting/design | Preparedness education/modeling; no eligible PHC facility or PHC workforce. |
| 28 | Exploring barriers and associated factors in the preparedness for cholera outbreak response in Public Health Centres of Addis Ababa, Ethiopia | 2025 | Include-primary | Eligible primary PHC study | Institution-based cross-sectional mixed-methods study covering all 101 public health centers; cholera outbreak preparedness and operational barriers. |
| 31 | Integrated assessment of hospital and community disaster preparedness in Dodoma municipality, Tanzania | 2025 | Exclude | Hospital/community setting | Facilities assessed were hospitals, combined with community participants; no eligible PHC facility analysis. |
| 34 | Preparedness of health facilities and their midwives for emergency obstetric and neonatal care, Accra, Ghana | 2025 | Exclude | Ineligible outcome | Emergency obstetric and neonatal care preparedness, not disaster/emergency-management preparedness. |
| 35 | PHC providers' perceived preparedness to respond to intimate partner violence | 2025 | Exclude | Ineligible outcome | Primary-care setting but outcome was intimate-partner-violence response, not disaster preparedness. |
| 38 | Smart greening initiatives for healthcare facilities: a Caribbean case study | 2025 | Exclude | Ineligible outcome | Greening and sustainability initiative rather than disaster preparedness or service continuity during emergencies. |
| 43 | Study protocol on disaster medicine preparedness among medical students, military medical school, Malaysia | 2024 | Exclude | Protocol and ineligible population | Study protocol without results; medical students rather than PHC personnel. |
| 56 | Hygiene and occupational safety of medical personnel and resilience of healthcare facilities in emergencies | 2024 | Exclude | Ineligible outcome/setting | Occupational hygiene and safety focus without an eligible PHC preparedness analysis. |
| 57 | Standardization of disaster preparedness of earthquake emergency drug in primary health institutions | 2024 | Exclude | Ineligible evidence type | Normative emergency-drug standardization report; no eligible empirical PHC preparedness assessment was identified. |
| 59 | Household flood disaster preparedness in rural and urban communities, Kaduna State, Nigeria | 2023 | Exclude | Ineligible population | Households and communities; no PHC setting or PHC workforce. |
| 62 | Evaluation index system of public health emergency preparedness capacity in county-level CDCs | 2023 | Exclude | Ineligible setting | County-level centers for disease control, not PHC facilities. |
| 65 | Liquefied Petroleum Gas Stations Disaster Risk Preparedness Assessment, Port Harcourt, Nigeria | 2023 | Exclude | Ineligible setting | Liquefied petroleum gas stations; no health-service or PHC setting. |
| 85 | School teachers' disaster preparedness, Angeles City, Philippines | 2021 | Exclude | Ineligible population | School teachers; no PHC facility or PHC workforce. |
| 100 | Assessment of Disaster Preparedness of Hospital in Mamasani, Iran | 2020 | Exclude | Hospital-only setting | Hospital disaster preparedness; not PHC. |
| 106 | Strengthen preparedness for public health emergency and response of Public Academic Medical Centers | 2020 | Exclude | Academic medical center/hospital setting | Public academic medical centers; not eligible PHC facilities. |
| 109 | Awareness and assessment of level of disaster preparedness among nurses of hospitals | 2019 | Exclude | Hospital workforce | Hospital nurses; not PHC personnel. |
| 129 | Remote Sensing in Multirisk Assessment: improving disaster preparedness | 2017 | Exclude | Ineligible setting/design | Remote-sensing and multirisk assessment; no PHC facility or PHC workforce. |

# Supplementary Table S4. Evidence-base evolution and bibliographic reconciliation after the retrieval and Q1–Q4 filter audits

| **Audit stage** | **Input** | **Action** | **Output** | **Interpretation** |
| --- | --- | --- | --- | --- |
| Original Watase evidence matrix | 41 Scopus-workflow studies + 26 supplementary records = 67 | Initial screening output | 67 retained records before bibliographic reconciliation | Historical process status |
| Bibliographic identity audit | 67 retained records before bibliographic reconciliation | Consolidated one duplicate entry and two preprint/final-publication pairs | 64 unique publications | Original audited evidence base |
| Pre-submission retrieval update | 32 reports initially recorded as not retrieved | Secured and locally verified 11 full-text copies; excluded one erratum and one report outside the healthcare/PHC context; documented 21 reports not retrieved in the Scopus pathway, including one identity already represented through a supplementary-source copy | 9 additional eligible unique publications | Two-reviewer-confirmed audit; 20 unreconciled report identities lacked full-text eligibility assessment |
| Updated combined matrix after retrieval audit | 64 previously unique + 9 newly eligible | DOI/title duplicate check found no overlap with the 64-publication matrix | 73 unique publications | 14 primary PHC; 59 contextual |
| Q1–Q4 filter audit | 24 records originally removed before eligibility screening | Reconstructed all record identities and reassessed against the prespecified criteria; one eligible record had no duplicate identity | 1 additional eligible primary PHC study; 23 exclusions | Final evidence base: 74 unique publications; 15 primary PHC; 59 contextual |

# Supplementary Table S5. Complete extraction, evidence-role, and Health EDRM mapping matrix of 74 included publications

| **No.** | **Study/source** | **Manuscript ref.** | **Evidence group** | **Study design** | **Source-derived coding basis** | **Mapped Health EDRM components** |
| --- | --- | --- | --- | --- | --- | --- |
| 1 | Lamberti-Castronuovo et al. (2022) | 3 | Contextual evidence synthesis | Systematic literature review | PSL: integrated PHC–H-EDRM strategy; PC: coordination and continuity planning; HR: multidisciplinary team; IKM: health information systems and telemedicine; HRS: continuity of care | PSL; PC; HR; IKM; HRS |
| 2 | Ward et al. (2024) | 4 | Contextual evidence synthesis | Final citation: empirical nursing study | PC: pre-disaster network coordination; HR: clinical leadership, teamwork, and staff support; FR: preparedness resource availability; HIL: medical products and technology | PC; HR; FR; HIL |
| 3 | Simsek et al. (2024) | 5 | Contextual evidence synthesis | Scoping review | PC: evacuation planning and provider roles; IKM: geographic information and preparedness data; HIL: access and evacuation infrastructure; CC: vulnerable-population and family education; ME: preparedness assessment | PC; IKM; HIL; CC; ME |
| 4 | van der Heijden et al. (2022) | 6 | Contextual evidence synthesis | Systematic review | PSL: water-emergency policy and standards; PC: stakeholder planning with water authorities and suppliers; HIL: basic WASH and emergency water infrastructure; CC: water-authority and supplier engagement | PSL; PC; HIL; CC |
| 5 | Almukhlifi et al. (2022) | 7 | Contextual evidence synthesis | Integrative review | HR: education and training, prior disaster experience, and employment status | HR |
| 6 | Su et al. (2023) | 8 | Contextual evidence synthesis | Systematic review | HR: declarative and procedural knowledge from structured education; HRS: systematic educational and mental-health support | HR; HRS |
| 7 | Alrowili et al. (2025) | 13 | Primary PHC synthesis | Explicit mixed methods | HR: technical knowledge, simulation, teamwork, emotional intelligence, and stress management | HR |
| 8 | Dg Sirua et al. (2025) | 14 | Primary PHC synthesis | Analytical cross-sectional study | Policy/planning competence, disaster knowledge and training, structural resilience, continuity of PHC services and collaboration with communities and other organizations. | PSL; PC; HR; IKM; HIL; HRS; CC |
| 9 | Derese et al. (2025) | 15 | Primary PHC synthesis | Institution-based cross-sectional mixed methods | PSL: implementation barriers and inconsistent application of cholera protocols/guidelines; PC: written preparedness plans, rapid response teams, and coordination mechanisms; HR: trained PHEM officers, rapid response teams, and staff motivation; FR: dedicated budget and financing barriers; IKM: posted case definitions, surveillance-data analysis, and timely disease reporting; HIL: ORS, intravenous fluids, antibiotics, safe water, latrines, waste disposal, handwashing facilities, and PPE; HRS: cholera case-management readiness and treatment capacity; ME: standardized 14-item facility preparedness assessment and sensitivity analysis across alternative thresholds. | PSL; PC; HR; FR; IKM; HIL; HRS; ME |
| 10 | Zarei et al. (2025) | 16 | Primary PHC synthesis | Qualitative FGD | PC: DART guideline usability and planning; HR: educational needs and organizational leadership; IKM: continuous needs assessment and health information; RC: early warning and risk communication; CC: cultural adaptation and community relevance | PC; HR; IKM; RC; CC |
| 11 | Lamberti-Castronuovo et al. (2024) | 17 | Primary PHC synthesis | Checklist development and cross-sectional field test | PC: incident plans, referral agreements, and transport arrangements; HR: multidisciplinary teams, surge staffing, and training; IKM: health information systems; RC: staff and community communication; HIL: PPE, stocks, and utilities; HRS: service integration and continuity; CC: community engagement; ME: operational preparedness checklist and repeated assessment | PC; HR; IKM; RC; HIL; HRS; CC; ME |
| 12 | Prakash et al. (2024) | 18 | Primary PHC synthesis | Cross-sectional KAP | PC: disaster mental-health preparedness planning; HR: disaster mental-health knowledge, attitudes, practices, psychological first-aid concepts, and clinical experience; HRS: preparedness knowledge concerning psychosocial triage and disaster mental-health response. | PC; HR; HRS |
| 13 | Fathoni et al. (2022) | 19 | Primary PHC synthesis | Correlative analytical observational cross-sectional study; internally inconsistent additional intervention/control and pre-post reporting | PC: health protocols; HR: protocol knowledge and staff self-control; IKM: disaster-preparedness questionnaire information; ME: DPQ-based preparedness assessment | PC; HR; IKM; ME |
| 14 | Lestari et al. (2022) | 20 | Primary PHC synthesis | Quantitative descriptive HSI facility assessment; multiple evidence sources | PSL: facility-safety governance and standards; PC: disaster committee and response plan; HR: training and committee capacity; FR: preparedness budgeting; HIL: structural and non-structural safety, water, fuel, medicines, and equipment; ME: Hospital Safety Index assessment | PSL; PC; HR; FR; HIL; ME |
| 15 | Litke et al. (2022) | 21 | Primary PHC synthesis | Qualitative FGD/interviews | PSL: primary-care resilience governance; HR: employee resilience and organizational adaptation; IKM: communication and organizational information; HIL: backup storage, masks, medicines, and disinfectants | PSL; HR; IKM; HIL |
| 16 | Firdausi et al. (2021) | 22 | Primary PHC synthesis | Multi-source quantitative descriptive HSI facility assessment | Governance, plans, staffing, information, warning and communication, utilities, stocks, essential services, community coordination and monitoring. | PSL; PC; HR; IKM; RC; HIL; HRS; CC; ME |
| 17 | Van Hoang et al. (2021) | 23 | Primary PHC synthesis | Cross-sectional mixed-methods study | Facility preparedness checklist, interviews and focus groups identified preparedness planning, staffing, financing, surveillance, communication, infrastructure, service delivery, community partnerships and evaluation. | PSL; PC; HR; FR; IKM; RC; HIL; HRS; CC; ME |
| 18 | Yari et al. (2021) | 24 | Primary PHC synthesis | Cross-sectional HSI | PSL: PHC safety standards; PC: response team and disaster plan; HR: continuous training; FR: preparedness financing; HIL: structural, non-structural, and functional safety; equipment, water, and food; HRS: service functionality; CC: community-related safety capacity; ME: Hospital Safety Index assessment | PSL; PC; HR; FR; HIL; HRS; CC; ME |
| 19 | Ndumbi et al. (2020) | 25 | Primary PHC synthesis | Analytical cross-sectional | PC: cholera protocols, patient transfer, and isolation; IKM: outbreak information and reporting; HIL: WASH, chlorine, sterilization, waste, ORS, and emergency fluids; HRS: cholera response services; ME: facility preparedness assessment | PC; IKM; HIL; HRS; ME |
| 20 | Lapcevic et al. (2019) | 26 | Primary PHC synthesis | PHC facility audit/analytical cross-sectional | PC: disaster plans; HR: staffing; FR: preparedness finance; IKM: telecommunications; HIL: structural integrity, utilities, and laboratory equipment; ME: Hospital Safety Index and 72-hour operational assessment | PC; HR; FR; IKM; HIL; ME |
| 21 | Hashikawa and Gold (2018) | 27 | Primary PHC synthesis | Qualitative/descriptive | PC: personal and clinic disaster plans; HR: leadership attitudes and attendance barriers; IKM: threat appraisal; ME: exercises and preparedness assessment | PC; HR; IKM; ME |
| 22 | Singh et al. (2026) | 29 | Contextual evidence synthesis | Scoping review | PSL: preparedness standards and safety policy; IKM: preparedness assessment instruments and data; HIL: structural, non-structural, and functional safety; ME: comparative preparedness assessment | PSL; IKM; HIL; ME |
| 23 | Ndembi et al. (2024) | 30 | Contextual evidence synthesis | Comment; rapid literature and desk review with expert consultation | PSL: governance and harmonization of regional and global pandemic prevention, preparedness and response (PPPR) financing mechanisms; FR: sustainable and equitable financing for resilient health systems and access to medical countermeasures. | PSL; FR |
| 24 | Awoyale et al. (2026) | 31 | Contextual evidence synthesis | Cross-sectional | HR: risk literacy and response capacity; RC: risk communication; HIL: WASH, transport, and hazard-exposed infrastructure; CC: interagency and community coordination | HR; RC; HIL; CC |
| 25 | Alfaifi and Hamaideh (2026) | 32 | Contextual evidence synthesis | Analytical cross-sectional | HR: nurses’ disaster knowledge, training, and experience | HR |
| 26 | Yadav et al. (2026) | 33 | Contextual evidence synthesis | Cross-sectional | PSL: legal and accreditation frameworks; PC: institutional preparedness procedures; HR: workforce education and preparedness culture; FR: preparedness resources and financing; HIL: facility infrastructure; HRS: emergency service readiness; ME: accreditation and compliance assessment | PSL; PC; HR; FR; HIL; HRS; ME |
| 27 | Natali et al. (2025) | 34 | Contextual evidence synthesis | Qualitative policy study | PSL: multilevel governance and policy design; PC: policy coordination and implementation; FR: recovery and resilience financing | PSL; PC; FR |
| 28 | Rao et al. (2025) | 35 | Contextual evidence synthesis | Expert opinion | PC: operational planning and coordination; HR: ethical decision-making, triage, and training; HIL: resources for austere response; CC: coordination with government and humanitarian actors | PC; HR; HIL; CC |
| 29 | Alrabie et al. (2025) | 36 | Contextual evidence synthesis | Systematic review | PC: standard disaster protocols; HR: education, workshops, and disaster-field experience; HIL: medical resources and logistics | PC; HR; HIL |
| 30 | Hauta et al. (2025) | 37 | Contextual evidence synthesis | Cross-sectional survey | PC: institutional disaster plans and collaboration; HR: knowledge, attitudes, and emergency-management experience; ME: preparedness assessment | PC; HR; ME |
| 31 | Abebe et al. (2025) | 38 | Contextual evidence synthesis | Systematic review | PC: flood evacuation and preparedness plans; FR: resource allocation for flood resilience; HIL: backup generators and flood-resilient infrastructure | PC; FR; HIL |
| 32 | Hong et al. (2025) | 39 | Contextual evidence synthesis | Secondary analysis | RC: disaster risk perception and communication; HIL: material preparedness resources and supplies | RC; HIL |
| 33 | Tran et al. (2025) | 40 | Contextual evidence synthesis | Conceptual framework | HR: health-worker digital and data literacy; IKM: interoperable human–animal health data and early warning | HR; IKM |
| 34 | Majambo et al. (2025) | 41 | Contextual evidence synthesis | Pilot cross-sectional | HIL: emergency medicines and equipment in dental practices; HRS: dental-practice readiness for medical emergencies. | HIL; HRS |
| 35 | Cvetkovic et al. (2024) | 42 | Contextual evidence synthesis | Analytical cross-sectional | PSL: standards and policy compliance; PC: EMS organization and procedures; HR: staffing and continuing education; HIL: equipment and vehicles; HRS: emergency response services; ME: response-time and performance assessment | PSL; PC; HR; HIL; HRS; ME |
| 36 | Lee and Lee (2024) | 43 | Contextual evidence synthesis | Spatial analysis | PSL: public-policy effectiveness; HIL: spatial distribution and robustness of health infrastructure | PSL; HIL |
| 37 | Aggarwal et al. (2024) | 44 | Contextual evidence synthesis | Qualitative study | HRS: stepped-care and service-delivery response; CC: rural access and community needs | HRS; CC |
| 38 | Tang (2024) | 45 | Contextual evidence synthesis | Agent-based modelling | IKM: machine-learning-supported modelling of airborne infection risk in healthcare facilities. | IKM |
| 39 | Guha et al. (2025) | 46 | Contextual evidence synthesis | Mixed methods | PSL: national policy and governance; HR: workforce adaptation; HRS: continuity of essential health services; CC: community- and patient-level continuity measures | PSL; HR; HRS; CC |
| 40 | Elaidy et al. (2023) | 47 | Contextual evidence synthesis | Analytical cross-sectional | HR: staff mental health, coping, and employment resilience; HIL: availability of PPE | HR; HIL |
| 41 | Noboa-Ramos et al. (2023) | 48 | Contextual evidence synthesis | Qualitative study | HIL: hurricane-related disruptions to power, water, communications, and critical supplies; HRS: effects on healthcare delivery and organizational recovery; CC: coordination between healthcare and social organizations and attention to patient social needs. | HIL; HRS; CC |
| 42 | Mukherji et al. (2022) | 49 | Contextual evidence synthesis | Descriptive cross-sectional | PC: governance, coordination, and risk-sharing arrangements in public-private partnership-supported primary care; CC: community-oriented primary-care service delivery through partnership arrangements. | PC; CC |
| 43 | Maghfiroh and Hanaoka (2022) | 50 | Contextual evidence synthesis | Stochastic modelling | HIL: mobile-clinic logistics and strategic location; HRS: mobile medical-service delivery | HIL; HRS |
| 44 | Pawar et al. (2024) | 51 | Contextual evidence synthesis | Systematic review | PC: domain-specific preparedness and coordination; HR: human resources and personnel protection; FR: financial preparedness; RC: communication; HIL: logistics and evacuation resources | PC; HR; FR; RC; HIL |
| 45 | Baker (2021) | 52 | Contextual evidence synthesis | Cross-sectional | PC: Incident Command System; HR: disaster terminology, triage ethics, and decontamination; IKM: surveillance; HIL: access to critical resources; HRS: nursing disaster-response readiness | PC; HR; IKM; HIL; HRS |
| 46 | Xiang et al. (2021) | 53 | Contextual evidence synthesis | Document analysis | PSL: language-access policy; HR: limited-English-proficiency and language capacity; CC: community language access | PSL; HR; CC |
| 47 | George and Anilkumar (2021) | 54 | Contextual evidence synthesis | Index development | PC: operational standard operating procedures; RC: coordination and communication indicators | PC; RC |
| 48 | Shiroya et al. (2021) | 55 | Contextual evidence synthesis | Mixed methods | IKM: medical records and HMIS; HIL: oxygen and facility readiness; HRS: continuity and integration of NCD services | IKM; HIL; HRS |
| 49 | Palinkas et al. (2021) | 56 | Contextual evidence synthesis | Qualitative rapid assessment | PC: search-and-rescue and blended pandemic–storm coordination; HRS: rapid preparedness-needs and resource response; CC: community resource assessment | PC; HRS; CC |
| 50 | Pourmohammadi et al. (2021) | 57 | Contextual evidence synthesis | Cross-sectional | PC: functional preparedness plan; HR: rapid-response team; FR: facility insurance and financial protection; IKM: hazard mapping; ME: facility preparedness assessment | PC; HR; FR; IKM; ME |
| 51 | Li et al. (2021) | 58 | Contextual evidence synthesis | Scientometric review | PC: governance and emergency-team organization; HR: emergency-team formation; IKM: scientometric knowledge mapping; HIL: adequacy of physical resources; ME: resilience assessment | PC; HR; IKM; HIL; ME |
| 52 | Wuthisuthimethawee et al. (2021) | 59 | Contextual evidence synthesis | Tool development | PSL: national standards embedded in the assessment tool; PC: mass-casualty and disaster plans; HR: health workforce and leadership; FR: emergency financing; IKM: information systems; HIL: medical products and technology; CC: community and pediatric considerations | PSL; PC; HR; FR; IKM; HIL; CC |
| 53 | Ibrahim et al. (2021) | 60 | Contextual evidence synthesis | Qualitative study | HR: health-system workforce resilience and familiarity | HR |
| 54 | Gilmore et al. (2020) | 61 | Contextual evidence synthesis | Rapid evidence review | RC: community communication and social-mobilization approaches for infectious-disease prevention and control; CC: community actors, participatory engagement, local ownership, and linkages with health-system stakeholders. | RC; CC |
| 55 | Oppong et al. (2020) | 62 | Contextual evidence synthesis | Cross-sectional | HR: infection-prevention workforce preparedness; HIL: PPE and sterile equipment; HRS: infection-prevention and control service readiness | HR; HIL; HRS |
| 56 | Hierink et al. (2020) | 63 | Contextual evidence synthesis | Geospatial modelling | IKM: GIS, travel-time, and accessibility modelling | IKM |
| 57 | Brewer et al. (2020) | 64 | Contextual evidence synthesis | Cross-sectional survey | HR: trauma, triage, leadership, and psychological preparedness; RC: regional and rural emergency communication; HRS: emergency-department response readiness | HR; RC; HRS |
| 58 | Sharma and Sharma (2020) | 65 | Contextual evidence synthesis | Descriptive cross-sectional | PC: critical-stabilization and isolation procedures; HR: staff retraining; HIL: isolation rooms and medication stock; HRS: critical-stabilization services; CC: community health-centre linkage | PC; HR; HIL; HRS; CC |
| 59 | Sultan et al. (2020) | 66 | Contextual evidence synthesis | Analytical cross-sectional; identity reconciled to Sultan et al. | HR: preparedness knowledge, confidence, willingness to work, and own/family safety considerations; IKM: self-rated knowledge and perceptions of risk and danger. | HR; IKM |
| 60 | Wagner et al. (2021) | 67 | Contextual evidence synthesis | Multicentre cross-sectional | HR: simulation-based pandemic and disaster readiness | HR |
| 61 | Sheikhbardsiri et al. (2019) | 68 | Contextual evidence synthesis | Descriptive cross-sectional | PC: operational exercise design and coordination; IKM: scenario information and operational uncertainty; RC: communication during the exercise; HIL: disaster-response resources; ME: exercise-based preparedness evaluation. | PC; IKM; RC; HIL; ME |
| 62 | Ballard et al. (2022) | 69 | Contextual evidence synthesis | Commentary | HR: trained, supervised, remunerated, and integrated community health worker workforce; RC: household risk communication and trusted community education; HIL: personal protective equipment, essential commodities, and supply-chain support; HRS: maintenance of community-based essential health services, surveillance, testing referral, and home-based care; CC: community engagement and links between communities and health systems; ME: community health worker data systems, performance monitoring, and evaluation. | HR; RC; HIL; HRS; CC; ME |
| 63 | Qari et al. (2019) | 70 | Contextual evidence synthesis | Systematic review/evaluation framework | PC: public-health preparedness-system coordination; HR: workforce capability; IKM: evaluation information; CC: system and community partners; ME: performance metrics | PC; HR; IKM; CC; ME |
| 64 | Thwala et al. (2018) | 71 | Contextual evidence synthesis | Facility assessment | PC: emergency obstetric and newborn-care coordination; HR: staffing; HIL: facility equipment and supplies; HRS: emergency obstetric services | PC; HR; HIL; HRS |
| 65 | Sabegh et al. (2017) | 72 | Contextual evidence synthesis | Neural-network modelling | HR: operational capacity in the healthcare supply chain; HIL: green disaster-response supply chain; ME: multi-objective performance optimization | HR; HIL; ME |
| 66 | Lucchini et al. (2017) | 73 | Contextual evidence synthesis | Comparative review | IKM: exposure assessment; HRS: lifetime health care; CC: affected populations; ME: systematic preparedness and long-term monitoring | IKM; HRS; CC; ME |
| 67 | Powell et al. (2016) | 74 | Contextual evidence synthesis | Methodological paper | IKM: dynamic threat and risk information; ME: auditable risk identification and assessment | IKM; ME |
| 68 | Bala et al. (2016) | 75 | Contextual evidence synthesis | Qualitative case study | PC: integrated information-technology architecture for disaster-response coordination; IKM: health-data integration and patient/information tracking; RC: information-technology-enabled emergency communication. | PC; IKM; RC |
| 69 | Sharifian et al. (2017) | 76 | Contextual evidence synthesis | Qualitative content analysis of national documents | Policy, planning, workforce development, financing, information, early warning, infrastructure, community education and evaluation. | PSL; PC; HR; FR; IKM; RC; HIL; CC; ME |
| 70 | Rahmati-Najarkolaei et al. (2017) | 77 | Contextual evidence synthesis | Analytical cross-sectional facility/staff assessment with maneuver observation | Incident command, roles, staff training, internal communication, logistics, triage, patient tracking and exercise evaluation. | PSL; PC; HR; IKM; HIL; HRS; ME |
| 71 | Theron and Smith (2026) | 78 | Contextual evidence synthesis | Textual expert article with case example | Risk governance, multisectoral planning, workforce capacity, hazard assessment, early warning, utilities, service continuity, community participation and evaluation. | PSL; PC; HR; IKM; RC; HIL; HRS; CC; ME |
| 72 | Adhikari et al. (2025) | 79 | Contextual evidence synthesis | Observational descriptive secondary analysis | Plans, committees, staffing, reporting systems, communication contacts, infrastructure, utilities, supplies, triage, essential services and monitoring. | PSL; PC; HR; IKM; RC; HIL; HRS; ME |
| 73 | Petrazzuoli et al. (2025) | 80 | Contextual evidence synthesis | Cross-sectional mixed-methods survey | Guidance, coordination, workforce resilience, information, communication, PPE and telehealth, service adaptation and community resilience groups. | PSL; PC; HR; IKM; RC; HIL; HRS; CC |
| 74 | Paganini et al. (2016) | 81 | Contextual evidence synthesis | Analytical cross-sectional telephone survey | Disaster plans, activation, command authority, staff roles and recall, information, surge arrangements, service response and periodic testing. | PSL; PC; HR; IKM; HIL; HRS; ME |

# Supplementary Table S6. Critical-appraisal procedures and design-specific summary of the 15 primary empirical PHC studies

All 15 primary empirical PHC studies were independently appraised from the complete full text by SNI and TR. Design-specific JBI critical-appraisal tools or MMAT 2018 were applied according to the study design and the evidence used in this review. Fathoni et al. was appraised with the JBI Analytical Cross-Sectional checklist because the article states a correlative analytical observational cross-sectional design and this review synthesized its cross-sectional associations. Its additional intervention/control and pre-post reporting was retained as an internal reporting inconsistency and was not reclassified as a separate intervention study. The appraisers completed their judgments independently, rechecked the source reports when judgments differed, and resolved all differences by consensus. The final consensus judgments are reported below. No pending appraisal labels remain.

## S6A. Appraisal procedure, tool allocation, and decision rules

| **Methodological item** | **Final specification** |
| --- | --- |
| **Primary appraisal scope** | Formal methodological appraisal was centered on the 15 primary empirical PHC studies. The 59 contextual publications were classified by evidence type and were not pooled into a directly comparable quality distribution. |
| **Appraisers** | SNI and TR. |
| **Independent process** | Each appraiser reviewed the complete full text and completed item-level judgments independently before consensus discussion. |
| **Tool allocation** | JBI Qualitative, JBI Analytical Cross-Sectional, and JBI Prevalence/Descriptive Facility Assessment checklists were used according to design. MMAT 2018 was used for mixed-methods and quantitative descriptive studies when appropriate. Fathoni et al. was allocated to JBI Analytical Cross-Sectional because the paper states a correlative observational cross-sectional design and the review used the cross-sectional associations; the contradictory intervention/control and pre-post reporting was treated as a reporting limitation. |
| **Judgment categories** | Yes, No, Unclear, or Not applicable (N/A). Unclear was used when the published report did not provide sufficient evidence; missing information was not inferred. |
| **Consensus process** | Differences were resolved through full-text rechecking and discussion. All final item-level decisions were agreed by SNI and TR. |
| **Overall interpretation** | No numerical JBI cut-off, percentage score, or global MMAT score was used. Narrative methodological judgments are review-defined interpretations of the pattern and importance of limitations and are not official JBI or MMAT grades. |
| **Audit trail** | The final item-level tables, consensus record, and appraisal decision rules are preserved in the supplementary material and retrospective OSF audit record. |

## S6B. Design-specific appraisal summary of the 15 primary empirical PHC studies

| **Study** | **Design** | **Tool** | **Final narrative judgment** | **Principal concerns** | **Two-reviewer status** |
| --- | --- | --- | --- | --- | --- |
| Alrowili et al. 2025 | Mixed methods | MMAT 2018 | Moderate-to-major concerns | Incomplete qualitative audit trail; uncertain representativeness and nonresponse; limited model-validation and integration reporting. | Independently completed and confirmed by SNI and TR |
| Dg Sirua et al. 2025 | Analytical cross-sectional survey | JBI Analytical Cross-Sectional | Moderate concerns | Incomplete eligibility criteria; limited validity evidence; mean-based dichotomization; insufficient confounder identification and control. | Independently completed and confirmed by SNI and TR |
| Derese et al. 2025 | Cross-sectional mixed methods | MMAT 2018 | Moderate concerns | Limited qualitative analytic detail; restricted measurement-validation evidence; equally weighted binary preparedness threshold; incomplete confounder specification; no explicit divergence analysis. | Independently completed and confirmed by SNI and TR |
| Zarei et al. 2025 | Qualitative FGD/guideline evaluation | JBI Qualitative | Moderate concerns | Limited reflexivity and weak participant-voice traceability. | Independently completed and confirmed by SNI and TR |
| Lamberti-Castronuovo et al. 2024 | Checklist development and descriptive facility assessment | JBI Prevalence | Major concerns | Purposive 10-facility sample; insufficient measurement validation and reliability evidence. | Independently completed and confirmed by SNI and TR |
| Prakash et al. 2024 | Analytical cross-sectional KAP survey | JBI Analytical Cross-Sectional | Moderate-to-major concerns | Convenience snowball sampling; unknown response rate; limited outcome validation; no confounder adjustment. | Independently completed and confirmed by SNI and TR |
| Fathoni et al. 2022 | Correlative analytical observational cross-sectional study with internally inconsistent additional reporting | JBI Analytical Cross-Sectional | Major concerns | Non-probability quota sampling; limited construct-specific validation detail; no explicit confounder framework or adjusted analysis; intervention/control and pre-post reporting cannot be reconciled with the stated cross-sectional design. | Independently completed and confirmed by SNI and TR |
| Lestari et al. 2022 | Quantitative descriptive multi-site HSI assessment | MMAT quantitative descriptive | Moderate concerns | Purposive 11-facility selection; limited representativeness; facility recruitment denominator not reported. | Independently completed and confirmed by SNI and TR |
| Litke et al. 2022 | Qualitative interviews and FGDs | JBI Qualitative | Low concerns | Explicit philosophical positioning was absent; otherwise methods and reporting were strong. | Independently completed and confirmed by SNI and TR |
| Firdausi et al. 2021 | Quantitative descriptive two-facility HSI assessment | MMAT quantitative descriptive | Major concerns | Accessibility-based selection of two facilities; incomplete technical expertise among informants; limited generalizability. | Independently completed and confirmed by SNI and TR |
| Van Hoang et al. 2021 | Cross-sectional mixed methods | MMAT 2018 | Moderate concerns | Uncertain city-wide representativeness; under-described qualitative analysis; subjective implementation ratings; no explicit divergence analysis. | Independently completed and confirmed by SNI and TR |
| Yari et al. 2021 | Province-wide descriptive facility assessment | JBI Prevalence | Low-to-moderate concerns | Broad coverage and standardized tool; assessor calibration and inter-rater reliability incompletely reported. | Independently completed and confirmed by SNI and TR |
| Ndumbi et al. 2020 | Analytical cross-sectional facility survey | JBI Analytical Cross-Sectional | Low-to-moderate concerns | Self-reported previous case history; incomplete conceptual confounder framework; restriction to reference facilities. | Independently completed and confirmed by SNI and TR |
| Lapcevic et al. 2019 | Single-facility HSI audit | JBI Prevalence/facility audit | Major external-validity concerns | Detailed operational case assessment, but no population-level generalizability. | Independently completed and confirmed by SNI and TR |
| Hashikawa and Gold 2018 | Descriptive mixed methods | MMAT 2018 | Moderate concerns | Very small purposive sample and unvalidated survey. | Independently completed and confirmed by SNI and TR |

## Supplementary Table S7. Item-level design-specific JBI and MMAT consensus appraisals S7A. MMAT 2018 mixed-methods appraisals

### Alrowili et al. (2025)

**Design assessed:** Mixed-methods study combining a structured survey, semi-structured interviews, drill observations, machine-learning prediction, and NLP-based qualitative analysis. **Appraisal tool:** MMAT 2018.

| **No.** | **Appraisal criterion** | **Final consensus judgment** | **Full-text evidence and location** | **Consensus rationale** |
| --- | --- | --- | --- | --- |
| S1 | Are there clear research questions? | Yes | The objectives specify assessment of technical competency, operational readiness, psychosocial resilience, predictive effects, and qualitative themes (pp. 4-5). | The quantitative, qualitative, and predictive objectives are explicitly stated. |
| S2 | Do the collected data allow the research questions to be addressed? | Yes | Survey data, semi-structured interviews, drill observations, machine-learning models, sentiment analysis, and topic modelling were used (pp. 5-9). | The planned data sources address the stated multidimensional objectives. |
| 1.1 | Is the qualitative approach appropriate to answer the qualitative research question? | Yes | Semi-structured interviews and narrative responses explored experiences, psychological challenges, communication barriers, and institutional gaps (pp. 7-9). | An exploratory qualitative approach was appropriate for perceived barriers and experiences. |
| 1.2 | Are qualitative data-collection methods adequate? | Unclear | The paper states that interviews were conducted in person or by videoconference, but does not clearly report the qualitative sample size, sampling strategy, interview guide development, duration, saturation, recording, or transcription procedures (pp. 7-9). | The data source is relevant, but procedural detail is insufficient for reproducibility. |
| 1.3 | Are qualitative findings adequately derived from the data? | Unclear | Sentiment analysis and LDA topic modelling were performed using NLTK and Gensim, but coding decisions, preprocessing choices, number of topics, analyst review, and qualitative validation procedures are incompletely reported (pp. 8-9). | The computational approach is described broadly, but the derivation of themes is not sufficiently auditable. |
| 1.4 | Is the interpretation of qualitative results sufficiently substantiated by data? | Unclear | Themes such as communication barriers, psychological safety, and training needs are reported, but participant quotations or detailed source excerpts are limited or absent in the retained report. | The reader cannot readily trace interpretations to participants' words. |
| 1.5 | Is there coherence between qualitative data sources, collection, analysis, and interpretation? | Unclear | The study links interview and narrative data to NLP analyses, but the relationship between semi-structured interviews, open-ended survey responses, and drill observations is not consistently distinguished (pp. 7-9). | The qualitative strand is relevant but incompletely delineated. |
| 4.1 | Is the sampling strategy relevant to address the quantitative research question? | Yes | A target sample of 400 was selected using stratified random sampling across occupational groups (p. 6). | The stated approach was appropriate for representing doctors, nurses, and administrative personnel. |
| 4.2 | Is the sample representative of the target population? | Unclear | The paper reports proportional stratification, but does not provide the total eligible workforce, sampling-frame construction, numbers approached, response rate, or comparison of respondents with nonrespondents (pp. 6-7). | Representativeness cannot be verified from the report. |
| 4.3 | Are the measurements appropriate? | Yes | A 45-item instrument was adapted from prior tools; psychosocial constructs were adapted from WLEIS, PSS, and TeamSTEPPS; pilot testing and reliability statistics were reported (pp. 7-9). | Measurement selection and internal consistency were reasonably supported. |
| 4.4 | Is the risk of nonresponse bias low? | Unclear | No number approached, response rate, refusal count, or nonresponse analysis was reported. | Nonresponse bias cannot be assessed. |
| 4.5 | Is the statistical analysis appropriate to answer the research question? | Unclear | Descriptive statistics, reliability tests, VIF, Random Forest, ANN, 80/20 train-test splitting, and reported performance metrics were used (pp. 8-15). However, external validation, cross-validation strategy, handling of class imbalance, missing-data detail, model tuning, and optimism correction are incompletely reported. | The analyses are broadly relevant, but reproducibility and model-validation reporting are incomplete. |
| 5.1 | Is there an adequate rationale for using a mixed-methods design? | Yes | The authors state that quantitative patterns were combined with interviews and observations to capture objective and subjective preparedness dimensions and to triangulate findings (pp. 5-8). | The rationale for combining methods is explicit. |
| 5.2 | Are the different components effectively integrated to answer the research question? | Unclear | Survey, interviews, drills, ML, and NLP are presented within one framework, but the precise point and method of integration are not consistently described (pp. 5-9). | Parallel use of methods is clear; formal mixed-methods integration is less clear. |
| 5.3 | Are the outputs of integration adequately interpreted? | Unclear | The discussion links psychosocial themes with preparedness prediction, but does not provide a joint display or explicit meta-inferences showing how qualitative and quantitative results changed or confirmed one another. | Integrated conclusions are plausible but not fully transparent. |
| 5.4 | Are divergences and inconsistencies between quantitative and qualitative results adequately addressed? | No | No explicit assessment of convergence, complementarity, or disagreement between survey, interview, drill-observation, and model outputs was reported. | Potential discordance between strands was not examined. |
| 5.5 | Do the different components adhere to the quality criteria of each methodological tradition? | Unclear | Quantitative reliability is reported, but sampling and model validation remain incompletely documented; the qualitative strand lacks sufficient procedural and evidentiary detail. | Quality limitations in both strands constrain confidence in the integrated findings. |

**Final narrative methodological judgment:** Moderate-to-major methodological concerns. Confidence is reduced by incomplete reporting of the qualitative sample and analytic audit trail, uncertain representativeness and nonresponse, incomplete machine-learning validation, and limited demonstration of formal mixed-methods integration.

### Hashikawa and Gold (2018)

**Design assessed:** Descriptive mixed-methods study using a written survey followed by semi-structured interviews. **Appraisal tool:** MMAT 2018.

| **No.** | **Appraisal criterion** | **Final consensus judgment** | **Full-text evidence and location** | **Consensus rationale** |
| --- | --- | --- | --- | --- |
| S1 | Are there clear research questions? | Yes | Purpose and hypothesis were clearly stated (pp. 1-2). | The study question was explicit. |
| S2 | Do the collected data allow the research questions to be addressed? | Yes | Survey and interview data directly addressed threat appraisal, personal plans, absenteeism, and attendance barriers (pp. 1-2). | Data were relevant to the objectives. |
| 1.1 | Is the qualitative approach appropriate? | Yes | Qualitative description and content analysis were used for perceptions and barriers (p. 2). | Approach matched the descriptive question. |
| 1.2 | Are qualitative data-collection methods adequate? | Yes | Semi-structured interviews were recorded and transcribed verbatim (p. 2). | Data collection was appropriate. |
| 1.3 | Are findings adequately derived from the data? | Yes | Two authors revised codes to consensus; themes were derived through content analysis (p. 2). | An explicit analytic process was reported. |
| 1.4 | Is interpretation sufficiently substantiated by data? | Yes | Results include participant quotations and quantitative context (pp. 2-3). | Interpretation is supported by data examples. |
| 1.5 | Is there coherence among sources, collection, analysis, and interpretation? | Yes | Survey responses were followed by probing interviews and integrated descriptively (pp. 1-3). | The qualitative strand was coherent. |
| 4.1 | Is the sampling strategy relevant to the quantitative question? | Yes | Purposeful sampling targeted clinical managers and medical directors from five clinics (p. 2). | The strategy captured decision-makers relevant to the question. |
| 4.2 | Is the sample representative of the target population? | No | Twelve leaders from five university-affiliated clinics were included (pp. 1-2). | The sample was very small and institutionally narrow. |
| 4.3 | Are measurements appropriate? | Unclear | The seven-question survey was newly developed from literature because no validated instrument existed (p. 2). | Content relevance was plausible, but validity and reliability were not established. |
| 4.4 | Is risk of non-response bias low? | Yes | All 12 invited leaders participated; one interview recording was lost (p. 2). | Survey response was complete, with limited qualitative data loss. |
| 4.5 | Is statistical analysis appropriate? | Yes | Descriptive statistics were appropriate for the small descriptive sample (p. 2). | No unsupported inferential analysis was used. |
| 5.1 | Is there an adequate rationale for mixed methods? | Yes | Interviews directly probed and explained written survey responses (pp. 1-2). | The qualitative strand added explanatory depth. |
| 5.2 | Are components effectively integrated? | Yes | Survey and interview findings were jointly evaluated and interpreted (p. 2). | Integration occurred at analysis and interpretation. |
| 5.3 | Are outputs of integration adequately interpreted? | Yes | Conclusions combine quantitative frequencies and qualitative barriers (pp. 2-4). | Integrated interpretations addressed the research question. |
| 5.4 | Are divergences between quantitative and qualitative findings addressed? | Unclear | The paper discusses mixed perceptions but does not provide a formal discordance analysis. | Handling of divergent findings was not explicit. |
| 5.5 | Do components adhere to quality criteria of each tradition? | Unclear | The qualitative component was credible, but the survey was unvalidated and the sample was very small. | Overall mixed-methods confidence is constrained by the quantitative strand. |

**Final narrative methodological judgment:** Moderate methodological concerns, driven primarily by the very small purposive sample and the unvalidated survey. The integration of survey and interview data was conceptually appropriate and transparently described.

### Van Hoang et al. (2021)

**Design assessed:** Cross-sectional mixed-methods study of 80 health facilities in four urban/peri-urban districts, complemented by in-depth interviews and two focus groups. **Appraisal tool:** MMAT 2018 - qualitative, quantitative descriptive, and mixed-methods domains.

| **No.** | **Appraisal criterion** | **Final consensus judgment** | **Full-text evidence and location** | **Consensus rationale** |
| --- | --- | --- | --- | --- |
| S1 | Are there clear research questions? | Yes | The study examined COVID-19 preparedness and response of Hanoi's primary healthcare system and identified enabling factors and barriers (pp. 1–2). | The quantitative and qualitative objectives are clear. |
| S2 | Do the collected data allow the research questions to be addressed? | Yes | The study assessed 80 health facilities using a CDC-based framework and collected interviews, focus groups, and secondary reports (pp. 2–3). | The complementary data sources address capability, facilitators, and barriers. |
| 1.1 | Is the qualitative approach appropriate to answer the qualitative research question? | Yes | In-depth interviews and focus groups were used to investigate barriers and enabling factors across governance, financing, workforce, supplies, and information systems (pp. 2–3). | Qualitative inquiry was appropriate for implementation experiences and contextual barriers. |
| 1.2 | Are the qualitative data-collection methods adequate? | Yes | The paper specifies interviewee categories at district and commune levels, representatives of local organizations and authorities, and two focus groups involving isolated and non-isolated communities. Interviews were audio-recorded and transcribed (pp. 2–3). | Participant groups and collection modes were relevant and adequately described, although interview duration and saturation were not reported. |
| 1.3 | Are the qualitative findings adequately derived from the data? | Unclear | Transcripts were analyzed 'by research topics,' but coding procedures, number of analysts, code development, and strategies for analytic rigor were not reported (p. 3). | The connection between raw transcripts and final themes is not sufficiently auditable. |
| 1.4 | Is the interpretation of qualitative results sufficiently substantiated by data? | Yes | The results include multiple participant quotations supporting themes on service continuity, PPE, workforce, governance, private-sector involvement, finance, and reporting burden (pp. 6–7). | Interpretations are supported by illustrative quotations. |
| 1.5 | Is there coherence between qualitative data sources, collection, analysis, and interpretation? | Yes | The study defined qualitative domains, interviewed relevant stakeholders, analyzed transcripts by those topics, and reported matching barrier/enabler themes (pp. 2–8). | The strand is coherent, although the analytic method is under-described. |
| 4.1 | Is the sampling strategy relevant to address the quantitative research question? | Yes | All 80 facilities in the four selected districts were included: four district health centers and 76 commune/ward health stations, spanning urban and peri-urban districts with and without positive cases (pp. 2–3). | The facility coverage was relevant to the selected-district comparison. |
| 4.2 | Is the sample representative of the target population? | No | Only four Hanoi districts were selected, and the paper does not describe probability selection of districts or demonstrate representativeness of the entire Hanoi primary healthcare system (pp. 2–3). | Complete facility coverage within selected districts does not establish city-wide representativeness. |
| 4.3 | Are the measurements appropriate? | Unclear | The US CDC Public Health Emergency Preparedness and Response Capabilities framework was adapted, but the authors acknowledge that completeness judgments were subjective and lacked clear quantitative criteria (pp. 2–3 and 9). | The conceptual framework was appropriate, but operational measurement validity and objectivity are uncertain. |
| 4.4 | Is the risk of nonresponse bias low? | Yes | The report presents completed questionnaires for all 80 selected facilities and does not report missing facility responses (pp. 2–3). | Within the selected districts, facility-level nonresponse appears minimal. |
| 4.5 | Is the statistical analysis appropriate to answer the research question? | Unclear | Descriptive statistics and comparisons between positive-case and non-positive-case areas were reported, but the specific inferential test used for p-values was not clearly identified and ordinal implementation ratings were summarized using means (pp. 3–5). | The descriptive analysis is appropriate, but the inferential procedure and treatment of ordinal ratings are insufficiently specified. |
| 5.1 | Is there an adequate rationale for using a mixed-methods design? | Yes | Quantitative facility capability assessment was paired with qualitative investigation of facilitators and barriers to explain implementation conditions (pp. 1–3). | The strands address complementary dimensions of the research objective. |
| 5.2 | Are the different components effectively integrated to answer the research question? | Yes | Capability scores and qualitative barrier/enabler themes are interpreted together in the results and discussion, particularly for workforce, governance, supplies, service delivery, and information systems (pp. 5–9). | Integration occurred at interpretation, although no formal joint display was provided. |
| 5.3 | Are the outputs of the integration adequately interpreted? | Yes | The conclusions synthesize quantitative gaps with qualitative explanations and identify coordinated priorities for evaluation, communication, volunteer management, workforce, and governance (pp. 8–9). | The combined interpretation is coherent and policy relevant. |
| 5.4 | Are divergences and inconsistencies between quantitative and qualitative results adequately addressed? | No | The paper does not explicitly examine disagreement or discordance between facility scores, stakeholder narratives, and secondary reports. | Mixed-methods divergence was not systematically assessed. |
| 5.5 | Do the different components adhere to the quality criteria of each methodological tradition? | Unclear | The qualitative strand has relevant participants and quotations but limited analytic detail; the quantitative strand has broad selected-district coverage but subjective measurement and incompletely specified inferential analysis. | Limitations in both strands constrain confidence in the integrated conclusions. |

### Derese et al. (2025)

Design assessed: Institution-based cross-sectional mixed-methods study covering all 101 public health centers, with five key-informant interviews and two focus groups. Appraisal tool: MMAT 2018 - qualitative, quantitative descriptive, and mixed-methods domains.

Final narrative methodological judgment: Moderate methodological concerns. The city-wide census and source-relevant checklist were strengths. Confidence is reduced by incomplete reporting of the qualitative analytic audit trail, limited measurement-validation evidence, an equally weighted binary preparedness threshold, incomplete confounder specification, and no explicit analysis of divergence between qualitative and quantitative strands.

| **No.** | **Appraisal criterion** | **Final consensus judgment** | **Full-text evidence and location** | **Consensus rationale** |
| --- | --- | --- | --- | --- |
| S1 | Are there clear research questions? | Yes | The study aimed to assess cholera outbreak preparedness, identify associated factors, and explore operational barriers through a mixed-methods approach (Introduction, p. 4). | The quantitative and qualitative objectives are explicit. |
| S2 | Do the collected data allow the research questions to be addressed? | Yes | A census assessment of 101 health centers, logistic regression, five key-informant interviews, and two focus groups addressed preparedness level, associated factors, and barriers (Methods, pp. 4-6). | The complementary data sources align with all stated objectives. |
| 1.1 | Is the qualitative approach appropriate to answer the qualitative research question? | Yes | A phenomenological qualitative component explored financial, workforce-motivation, and multisectoral-coordination barriers (Methods and Results, pp. 4, 8-9). | An exploratory qualitative approach was appropriate for operational experiences and barriers. |
| 1.2 | Are qualitative data-collection methods adequate? | Yes | Five purposively selected key-informant interviews and two focus groups used semi-structured guides; data collection continued to saturation, and interviews were recorded, transcribed, translated, and supported by participant quotations (Methods, pp. 4-6; Results, pp. 8-9). | The sampling and collection procedures were broadly adequate, although duration and guide-development details were limited. |
| 1.3 | Are qualitative findings adequately derived from the data? | Unclear | The report states that thematic analysis was used, but coding procedures, number of analysts, codebook development, and theme-validation steps were not described in detail (Methods, p. 6). | The themes are plausible, but the analytic audit trail is incomplete. |
| 1.4 | Is the interpretation of qualitative results sufficiently substantiated by data? | Yes | Each of the three themes is supported by multiple direct quotations from FGD and KII participants (Results, pp. 8-9). | Participant quotations provide clear source-to-theme traceability. |
| 1.5 | Is there coherence between qualitative data sources, collection, analysis, and interpretation? | Yes | The semi-structured interviews and focus groups addressed the specified barriers, and the thematic findings were interpreted alongside facility preparedness results (pp. 6, 8-10). | The qualitative strand is coherent despite limited analytic detail. |
| 4.1 | Is the sampling strategy relevant to address the quantitative research question? | Yes | A total-population approach included all 101 eligible and functioning public health centers in Addis Ababa (Methods, pp. 4-5). | A city-wide census was highly relevant to estimating facility preparedness. |
| 4.2 | Is the sample representative of the target population? | Yes | All eligible functioning public health centers under the city health bureau were included; only centers under construction, not providing service, or operating as cholera treatment centers during data collection were excluded (Methods, p. 5). | Coverage closely matched the defined target population. |
| 4.3 | Are the measurements appropriate? | Unclear | The 14-item tool was adapted from the Ethiopian national guideline and WHO cholera-readiness checklist and was pretested, but construct validation, reliability, and justification for equal item weighting were not fully reported (Methods, pp. 5-6; Limitations, p. 10). | Content relevance was strong, but measurement-property evidence was incomplete. |
| 4.4 | Is the risk of nonresponse bias low? | Yes | Data were analyzed for all 101 eligible health centers; no facility nonresponse was reported (Results, p. 7). | The census approach minimized facility-level nonresponse bias. |
| 4.5 | Is the statistical analysis appropriate to answer the research question? | Unclear | Descriptive statistics, sensitivity analyses, and binary logistic regression were used; however, the conceptual confounder framework was limited and only a small set of candidate variables entered the multivariable model (Methods and Results, pp. 6-8). | The analysis addressed the stated questions, but residual confounding and model specification remain concerns. |
| 5.1 | Is there an adequate rationale for using a mixed-methods design? | Yes | The design estimated preparedness quantitatively and used qualitative data to explain operational barriers (Introduction and Methods, pp. 4-6). | The two strands addressed complementary parts of the research objective. |
| 5.2 | Are the different components effectively integrated to answer the research question? | Yes | Financial barriers, staff motivation, and multisectoral cooperation were used to interpret the facility preparedness level and the association with response plans (Results and Discussion, pp. 8-10). | Integration was visible at the interpretation stage. |
| 5.3 | Are the outputs of the integration adequately interpreted? | Yes | The discussion links the quantitative preparedness estimate and plan association with qualitative explanations concerning finance, motivation, and coordination (pp. 9-10). | The integrated interpretation is coherent and operationally meaningful. |
| 5.4 | Are divergences and inconsistencies between quantitative and qualitative results adequately addressed? | Unclear | No explicit procedure for identifying or resolving convergence, complementarity, or divergence between the two strands was reported. | The strands were complementary, but formal divergence analysis was absent. |
| 5.5 | Do the different components adhere to the quality criteria of each tradition? | Unclear | The city-wide quantitative census was strong, but measurement validation, confounder specification, and the qualitative analytic audit trail were incomplete. | Method-specific limitations reduce overall mixed-methods confidence. |

**Final narrative methodological judgment:** Moderate methodological concerns. The study has strong facility coverage within four diverse districts and provides useful stakeholder quotations and mixed-methods interpretation. Limitations include uncertain city-wide representativeness, under-described qualitative analysis, subjective implementation ratings, incomplete specification of inferential testing, and no explicit analysis of divergence between strands.

## S7B. MMAT 2018 quantitative descriptive appraisals

### Lestari et al. (2022)

**Design assessed:** Quantitative descriptive multi-site PHC safety assessment using interviews, FGDs, observation, and document review to generate WHO/PAHO HSI scores; no separately analyzed qualitative strand. **Appraisal tool:** MMAT 2018 - quantitative descriptive domain.

| **No.** | **Appraisal criterion** | **Final consensus judgment** | **Full-text evidence and location** | **Consensus rationale** |
| --- | --- | --- | --- | --- |
| S1 | Are there clear research questions? | Yes | The study asks whether PHCs are adequately prepared, what their preparedness levels are, and which variables are weakest (pp. 3–4). | The questions are explicit and aligned with the facility assessment. |
| S2 | Do the collected data allow the research questions to be addressed? | Yes | Eleven PHCs in four provinces were assessed using WHO/PAHO HSI modules through multidisciplinary interviews, FGDs, observations, and document review (pp. 4–7). | The data directly address the stated preparedness questions. |
| 4.1 | Is the sampling strategy relevant to address the quantitative research question? | Yes | Eleven PHCs were purposively selected from four disaster-prone provinces based on vulnerability, study objectives, and agreement with local health authorities (pp. 4–5). | Purposive selection is relevant for an exploratory assessment of vulnerable PHCs, although it is not suitable for national prevalence inference. |
| 4.2 | Is the sample representative of the target population? | No | The sample comprised 11 purposively selected PHCs from four provinces; no national or provincial sampling frame or probability selection was used (pp. 4–5). | The selected facilities cannot represent all Indonesian PHCs. |
| 4.3 | Are the measurements appropriate? | Yes | The WHO/PAHO HSI for medium and small facilities was used, covering 166 items. Four trained multidisciplinary assessors collected data from documents, interviews, FGDs, and direct/virtual observations (pp. 5–7). | The instrument and multi-source assessment process were directly relevant and reasonably standardized. |
| 4.4 | Is the risk of nonresponse bias low? | Unclear | The report identifies 11 participating PHCs but does not state how many facilities were approached, refused, or were otherwise eligible (pp. 4–5). | The absence of a facility recruitment flow prevents assessment of nonresponse or participation bias. |
| 4.5 | Is the statistical analysis appropriate to answer the research question? | Yes | HSI items were scored and weighted according to the guidance, and univariate descriptive analyses and category classifications were reported (pp. 7–8). | The analysis is appropriate for a descriptive facility-safety assessment. |

**Final narrative methodological judgment:** Moderate methodological concerns. The study used an internationally recognized HSI framework, multidisciplinary assessors, and triangulated facility evidence. The principal limitations are purposive selection, lack of representativeness, and insufficient reporting of the facility recruitment denominator.

### Firdausi et al. (2021)

**Design assessed:** Quantitative descriptive two-facility HSI assessment using interviews, observation, and document review; despite the authors' mixed-method label, no separately analyzed qualitative strand was reported. **Appraisal tool:** MMAT 2018 - quantitative descriptive domain.

| **No.** | **Appraisal criterion** | **Final consensus judgment** | **Full-text evidence and location** | **Consensus rationale** |
| --- | --- | --- | --- | --- |
| S1 | Are there clear research questions? | Yes | The stated aim was to identify the disaster preparedness level of public health centers in DKI Jakarta Province (pp. 1–2). | The descriptive objective is clear. |
| S2 | Do the collected data allow the research questions to be addressed? | Yes | Two facilities were assessed using the PAHO HSI through interviews, observations, and document review, producing structural, nonstructural, functional, and overall scores (pp. 3–4). | The data permit case-level assessment of the two selected Puskesmas, although not a province-wide estimate. |
| 4.1 | Is the sampling strategy relevant to address the quantitative research question? | No | Puskesmas X and Y were purposively selected primarily because they were accessible from Depok during large-scale COVID-19 restrictions and were recommended by the provincial health office (p. 3). | Accessibility-based selection of two facilities is not adequate for a province-wide preparedness question. |
| 4.2 | Is the sample representative of the target population? | No | Only two South Jakarta Puskesmas were assessed; no probability sample or evidence of representativeness of DKI Jakarta PHCs was provided (p. 3). | The findings are limited to the two selected cases. |
| 4.3 | Are the measurements appropriate? | Unclear | The PAHO HSI for medium and small facilities was used and triangulated through interviews, observation, and document review. However, the authors report that informants often needed confirmation from other staff, no civil-engineering/architecture informant was available, and one facility lacked an emergency-response team (pp. 3 and 8). | The tool is appropriate, but assessor/informant expertise and completeness for structural and functional modules were limited. |
| 4.4 | Is the risk of nonresponse bias low? | Unclear | The report does not provide the number of facilities approached, refusal information, or a participant/informant response flow (p. 3). | Participation and nonresponse bias cannot be evaluated. |
| 4.5 | Is the statistical analysis appropriate to answer the research question? | Yes | The study used univariate presentation and the prescribed HSI module and overall score calculations with categories A–C (pp. 3–4). | The analysis is appropriate for descriptive assessment of the two facilities, but not for province-wide inference. |

**Final narrative methodological judgment:** Major concerns for representativeness and moderate concerns for measurement completeness. The PAHO HSI and multi-source triangulation support the two facility assessments, but accessibility-based purposive selection, the very small sample, incomplete technical expertise among informants, and absence of a recruitment flow prevent generalization to DKI Jakarta Province.

## S7C. JBI qualitative appraisals

### Zarei et al. (2025)

**Design assessed:** Qualitative content analysis and FGD-based guideline case study using Eisner's educational criticism model. **Appraisal tool:** JBI Checklist for Qualitative Research.

| **No.** | **Appraisal criterion** | **Final consensus judgment** | **Full-text evidence and location** | **Consensus rationale** |
| --- | --- | --- | --- | --- |
| 1 | Is there congruity between the philosophical perspective and methodology? | Unclear | Eisner's model and qualitative content analysis were described, but an explicit philosophical position was not stated (pp. 2-3). | Philosophical congruity cannot be fully assessed. |
| 2 | Is there congruity between methodology and research question/objectives? | Yes | The study aimed to critically evaluate the DART guideline and used FGD-based educational criticism (p. 3). | The qualitative approach matched the evaluative objective. |
| 3 | Is there congruity between methodology and data-collection methods? | Yes | Seven purposively selected experts reviewed the guideline and participated in a recorded FGD (p. 3). | Data collection aligned with expert qualitative evaluation. |
| 4 | Is there congruity between methodology and representation/analysis of data? | Yes | Data were organized into description, interpretation, evaluation, and thematic dimensions (pp. 3-14). | Analysis followed the stated model. |
| 5 | Is there congruity between methodology and interpretation of results? | Yes | Interpretations and themes were explicitly linked to the educational criticism framework (pp. 3-14). | Interpretation was methodologically aligned. |
| 6 | Is the researcher culturally or theoretically located? | Unclear | Expertise of participants was described, but researcher positioning was not reported. | Potential interpretive standpoint is not transparent. |
| 7 | Is researcher influence, and vice versa, addressed? | No | Purposeful selection by the researcher was reported, but reflexivity and influence on the FGD/analysis were not discussed (p. 3). | Reflexive safeguards were absent. |
| 8 | Are participants and their voices adequately represented? | Unclear | Themes and judgments are presented, but individual participant quotations are limited or absent. | The audit trail from participant statements to themes is not strong. |
| 9 | Is the research ethical according to current criteria? | Yes | Ethics committee approval and code IR.MUI.NUREMA.REC.1402.007 were reported (p. 14). | Formal approval was documented. |
| 10 | Do conclusions flow from the analysis or interpretation? | Yes | Conclusions reflect the identified visual, content, cultural, and educational themes (pp. 14-15). | Conclusions are consistent with the reported analysis. |

**Final narrative methodological judgment:** Moderate concerns, mainly due to limited reflexivity and weak presentation of participant voices. The methodology, analytic framework, and conclusions were otherwise coherent.

### Litke et al. (2022)

**Design assessed:** Qualitative observational study using interviews and focus groups. **Appraisal tool:** JBI Checklist for Qualitative Research.

| **No.** | **Appraisal criterion** | **Final consensus judgment** | **Full-text evidence and location** | **Consensus rationale** |
| --- | --- | --- | --- | --- |
| 1 | Is there congruity between philosophical perspective and methodology? | Unclear | No explicit philosophical perspective was stated, although qualitative description and thematic analysis were specified (pp. 2-4). | Philosophical positioning was not reported. |
| 2 | Is methodology congruent with the research questions? | Yes | Questions concerned perceived crises and management strategies; qualitative methods were appropriate (p. 2). | The design matched exploratory aims. |
| 3 | Is methodology congruent with data collection? | Yes | Purposive and snowball sampling, semi-structured interviews, focus groups, recordings, and verbatim transcription were described (pp. 2-4). | Data collection was appropriate and detailed. |
| 4 | Is methodology congruent with representation and analysis? | Yes | Rapid qualitative analysis was followed by inductive thematic analysis with MAXQDA (p. 4). | Analysis was clearly described. |
| 5 | Is methodology congruent with interpretation? | Yes | Themes and strategies are interpreted within primary-care crisis resilience (pp. 5-13). | Interpretation is consistent with the analysis. |
| 6 | Is the researcher culturally or theoretically located? | Yes | Interviewers' backgrounds, experience, age, and professional roles were reported (p. 3). | Researcher positioning was unusually transparent. |
| 7 | Is researcher influence addressed? | Yes | Prior professional relationships were disclosed; team discussions were used to reduce bias (pp. 3-4). | Reflexive and team-based safeguards were described. |
| 8 | Are participants and their voices represented? | Yes | Extensive representative quotations accompany themes (pp. 5-10). | Participant voices are clearly visible. |
| 9 | Is the research ethical? | Yes | Ethics approval S-456/2021 and written informed consent were reported (pp. 2, 13). | Ethical requirements were met. |
| 10 | Do conclusions flow from the analysis? | Yes | Conclusions reflect reported crisis perceptions and strategies (pp. 11-13). | Conclusions are well grounded. |

**Final narrative methodological judgment:** Low methodological concerns. The main limitation is the absence of an explicit philosophical stance; otherwise, sampling, researcher reflexivity, data collection, analysis, and reporting were strong.

## S7D. JBI analytical cross-sectional appraisals

### Prakash et al. (2024)

**Design assessed:** Analytical cross-sectional online KAP survey of primary-care physicians. **Appraisal tool:** JBI Checklist for Analytical Cross-Sectional Studies.

| **No.** | **Appraisal criterion** | **Final consensus judgment** | **Full-text evidence and location** | **Consensus rationale** |
| --- | --- | --- | --- | --- |
| 1 | Were inclusion criteria clearly defined? | Yes | Registered PCPs working in Karnataka were eligible; the survey was distributed across 29 districts (p. 2). | Eligibility was sufficiently stated. |
| 2 | Were subjects and setting described in detail? | Yes | Setting, districts, recruitment, qualifications, age, sex, and experience were reported (pp. 1-3). | Population and context were adequately described. |
| 3 | Was exposure measured validly and reliably? | Yes | Clinical experience and demographic exposures were directly self-reported using standardized survey fields (p. 2). | The main exposure variables were straightforward, although self-reported. |
| 4 | Were objective standard criteria used for measurement of the condition? | N/A | No diagnostic condition was assessed. | This criterion is not applicable to a KAP survey. |
| 5 | Were confounding factors identified? | No | Potential confounders were not prospectively identified; analyses focused on bivariate correlations (pp. 2-3). | Confounding could influence observed associations. |
| 6 | Were strategies to deal with confounding stated? | No | No multivariable analysis, stratification, or adjustment was reported. | Associations remain unadjusted. |
| 7 | Were outcomes measured validly and reliably? | Unclear | The 30-item questionnaire underwent expert face/content review, but reliability and construct validation were not reported (p. 2). | Outcome measurement validity is only partially supported. |
| 8 | Was appropriate statistical analysis used? | Yes | Descriptive statistics, normality testing, and Spearman correlations were appropriate for the reported distributions (p. 2). | The analysis fit the bivariate research questions, although it did not control confounding. |

**Final narrative methodological judgment:** Moderate-to-major concerns because convenience snowball sampling, an unknown response rate, limited outcome-scale validation, and unadjusted analyses restrict generalizability and causal interpretation.

### Ndumbi et al. (2020)

**Design assessed:** Descriptive and analytical cross-sectional survey of 180 primary healthcare centers. **Appraisal tool:** JBI Checklist for Analytical Cross-Sectional Studies.

| **No.** | **Appraisal criterion** | **Final consensus judgment** | **Full-text evidence and location** | **Consensus rationale** |
| --- | --- | --- | --- | --- |
| 1 | Were inclusion criteria clearly defined? | Yes | The study population was official reference HCs in Kinshasa; selection criteria and sampling frame were defined (p. 3). | Eligibility was clear. |
| 2 | Were subjects and setting described in detail? | Yes | Kinshasa health zones, health areas, reference HCs, and outbreak context were described (pp. 2-3). | Setting was well characterized. |
| 3 | Was exposure measured validly and reliably? | Unclear | Cholera-case reporting status was obtained by interview with head nurses and was not independently verified (pp. 3-4). | Self-report may have introduced misclassification. |
| 4 | Were objective standard criteria used for outcome measurement? | Yes | Fourteen preparedness criteria were based on cholera-kit and WASHFIT standards and assessed through interview and inspection (pp. 3-4). | Preparedness criteria had explicit operational definitions. |
| 5 | Were confounding factors identified? | Unclear | Variables were selected statistically from bivariate analyses, but a conceptual confounding framework was not specified (p. 4). | Important contextual confounders may have been omitted. |
| 6 | Were strategies to deal with confounding stated? | Yes | A multivariable logistic regression model was used and model fit was assessed with Hosmer-Lemeshow testing (p. 4). | Adjustment strategy was reported. |
| 7 | Were outcomes measured validly and reliably? | Yes | Trained WHO/MoH teams interviewed head nurses, inspected facilities, and applied a structured questionnaire (pp. 3-4). | Measurement procedures were standardized and triangulated. |
| 8 | Was appropriate statistical analysis used? | Yes | Proportions, confidence intervals, logistic regression, adjusted odds ratios, and model-fit testing were reported (pp. 4-5). | Analysis was appropriate for the questions. |

**Final narrative methodological judgment:** Low-to-moderate concerns. Sampling, measurement, and analysis were strong; residual concerns relate to self-reported case history, incomplete conceptual control of confounding, and restriction to official reference facilities.

### Dg Sirua et al. (2025)

**Design assessed:** Analytical cross-sectional survey of 125 nurses sampled proportionally from 14 Puskesmas in Palu City. **Appraisal tool:** JBI Critical Appraisal Checklist for Analytical Cross-Sectional Studies.

| **No.** | **Appraisal criterion** | **Final consensus judgment** | **Full-text evidence and location** | **Consensus rationale** |
| --- | --- | --- | --- | --- |
| 1 | Were the criteria for inclusion in the sample clearly defined? | Unclear | The population was 158 nurses working in 14 Puskesmas, and 125 were sampled; however, explicit participant inclusion and exclusion criteria such as employment status, minimum tenure, or availability were not reported (pp. 3–4). | The sampling population is identified, but reproducible eligibility criteria are incomplete. |
| 2 | Were the study subjects and setting described in detail? | Yes | The study identifies Palu City, the January–June 2024 period, all 14 Puskesmas, the population and proportional sample per Puskesmas, and participant age and sex distributions (pp. 3–6). | The setting, facility strata, sampling allocation, and basic participant characteristics are adequately described. |
| 3 | Was the exposure measured in a valid and reliable way? | Unclear | Disaster-risk understanding and policy/planning competency were measured using structured ICN–WHO-derived items; internal consistency was reported as α=0.765 for 17 items and α=0.794 for 6 items. Evidence of construct validation, translation/adaptation procedures, or criterion validity was not reported (p. 5). | Reliability is reported, but validity and adaptation procedures are insufficiently documented. |
| 4 | Were objective, standard criteria used for measurement of the condition? | N/A | No clinical or diagnostic condition was being identified; the study measured preparedness competencies and organizational resilience. | This criterion is not applicable to a non-diagnostic preparedness survey. |
| 5 | Were confounding factors identified? | No | The report analyzed two competency predictors. Age, sex, work tenure, professional education, Puskesmas characteristics, previous disaster experience, and training were not identified as potential confounders (pp. 5–7). | No conceptual or empirical confounder framework was presented. |
| 6 | Were strategies to deal with confounding factors stated? | Unclear | A logistic-regression model included the two competency variables, but no strategy for selecting or adjusting for potential demographic, professional, or facility-level confounders was reported (pp. 5–7). | Multivariable analysis was used, but it did not clearly function as confounder control. |
| 7 | Were the outcomes measured in a valid and reliable way? | Unclear | Puskesmas resilience was measured using 18 items derived from a Health Centre Resilience Framework, with α=0.769. The paper does not report construct validation, adaptation, criterion validity, or whether nurse perceptions accurately represent facility-level resilience (p. 5). | Internal consistency is acceptable, but the validity of a staff-reported facility-resilience outcome remains uncertain. |
| 8 | Was appropriate statistical analysis used? | Yes | Frequencies, chi-square tests, and logistic regression with odds ratios and 95% confidence intervals were reported. Variables were dichotomized at their sample means, and the model included only two predictors (pp. 5–7). | The tests are broadly appropriate for the categorized cross-sectional data, although dichotomization and incomplete adjustment limit interpretation. |

**Final narrative methodological judgment:** Moderate methodological concerns. Strengths include proportional stratified random sampling across all 14 Puskesmas, reported internal consistency, and appropriate basic association analyses. Confidence is limited by incomplete eligibility criteria, limited validity evidence for exposure and outcome instruments, mean-based dichotomization, and insufficient identification and control of confounding.

## S7E. JBI prevalence and descriptive facility-assessment appraisals

### Lamberti-Castronuovo et al. (2024)

**Design assessed:** Checklist development followed by a descriptive cross-sectional assessment of 10 purposively selected primary-care facilities. **Appraisal tool:** JBI Checklist for Prevalence Studies.

| **No.** | **Appraisal criterion** | **Final consensus judgment** | **Full-text evidence and location** | **Consensus rationale** |
| --- | --- | --- | --- | --- |
| 1 | Was the sample frame appropriate to address the target population? | No | Ten selected facilities in one province; the target population was broader PHC preparedness (pp. 2-3). | The frame did not enumerate all eligible facilities and cannot support province-wide prevalence inference. |
| 2 | Were study participants/facilities sampled appropriately? | No | Purposive critical-case sampling based on geography, demography, and service type (p. 2). | Purposive selection was reasonable for exploratory testing but not for representative prevalence estimation. |
| 3 | Was the sample size adequate? | No | Ten facilities were assessed; no sample-size calculation was reported (pp. 2-3). | Adequacy for prevalence or stable component estimates was not demonstrated. |
| 4 | Were the study setting and facilities described in detail? | Yes | Italian PHC organization, facility-selection criteria, and service types were described (pp. 2-3). | Context and facility types were sufficiently reported for interpretation. |
| 5 | Was analysis conducted with sufficient coverage of the identified sample? | Unclear | All selected facilities appear to have been analyzed, but the eligible denominator and recruitment flow were not reported (pp. 2-3). | Completeness within the selected sample is likely, but coverage cannot be verified. |
| 6 | Were valid methods used for identification of preparedness characteristics? | Unclear | A newly developed checklist was based on literature and expert consultation; formal validation and weighting were not completed (pp. 1-3, 6). | Content development was systematic, but criterion validity and psychometric performance were not established. |
| 7 | Was preparedness measured in a standard, reliable way for all facilities? | Unclear | The main researcher conducted interviews and visual inspections; no inter-rater reliability or repeatability assessment was reported (p. 2). | Administration was standardized in principle, but measurement reliability remains uncertain. |
| 8 | Was the statistical analysis appropriate? | Yes | Exploratory descriptive analysis in Excel was explicitly used (p. 2). | Descriptive analysis matched the stated exploratory aim. |
| 9 | Was the response rate adequate and managed appropriately? | Unclear | The number approached, refusals, and reasons for non-participation were not reported. | Selection and non-response bias cannot be evaluated. |

**Final narrative methodological judgment:** Major concerns for representativeness and measurement validation. The study is valuable as an exploratory checklist-development and feasibility assessment, but its frequencies should not be interpreted as population prevalence.

### Yari et al. (2021)

**Design assessed:** Province-wide descriptive cross-sectional safety assessment of 805 PHC facilities. **Appraisal tool:** JBI Checklist for Prevalence Studies.

| **No.** | **Appraisal criterion** | **Final consensus judgment** | **Full-text evidence and location** | **Consensus rationale** |
| --- | --- | --- | --- | --- |
| 1 | Was the sample frame appropriate? | Yes | The study assessed PHC facilities across Kurdistan Province and described facility types and provincial structure (pp. 2-3). | The frame corresponded closely to the target facility population. |
| 2 | Were facilities sampled appropriately? | Yes | The report indicates comprehensive assessment of 805 PHC facilities rather than a convenience subset (pp. 1, 3). | Coverage was broad and appropriate. |
| 3 | Was the sample size adequate? | Yes | A very large facility sample of 805 was included (p. 1). | Precision and stability of descriptive estimates were likely adequate. |
| 4 | Were facilities and setting described in detail? | Yes | Iranian PHC structure, facility types, and provincial context were described (pp. 2-3). | Context was sufficiently detailed. |
| 5 | Was analysis conducted with sufficient coverage? | Yes | Structural, non-structural, functional, and total safety were reported across the included facilities (pp. 3-4). | Analysis covered the intended domains and sample. |
| 6 | Were valid methods used to identify preparedness/safety? | Yes | A Ministry of Health PHC safety-evaluation checklist with defined scoring classes was used (pp. 1, 3-4). | The method was standardized and directly relevant. |
| 7 | Was preparedness measured in a standard, reliable way? | Unclear | A standardized checklist was used, but assessor training, calibration, and inter-rater reliability were not fully reported. | Reliability across assessors cannot be confirmed. |
| 8 | Was statistical analysis appropriate? | Yes | Descriptive scoring and safety-classification procedures matched the study aim (pp. 3-4). | Analysis was appropriate. |
| 9 | Was response/coverage adequately managed? | Yes | The large province-wide facility assessment did not report important non-response or missing-facility concerns. | Coverage appears adequate, although a formal flow diagram was not provided. |

**Final narrative methodological judgment:** Low-to-moderate concerns. The major strength is broad facility coverage using a standardized instrument; the main uncertainty is assessor reliability and calibration.

### Lapcevic et al. (2019; published online 2018)

**Design assessed:** Single-facility descriptive HSI safety audit and retrospective disaster case assessment. **Appraisal tool:** JBI Checklist for Prevalence Studies, transparently applied as a facility-audit appraisal.

| **No.** | **Appraisal criterion** | **Final consensus judgment** | **Full-text evidence and location** | **Consensus rationale** |
| --- | --- | --- | --- | --- |
| 1 | Was the sample frame appropriate to address the target population? | No | One PHC in Obrenovac was assessed (pp. 1-2). | The study cannot estimate preparedness prevalence across PHCs. |
| 2 | Was the facility sampled appropriately? | No | The facility was selected as a disaster-affected case; no sampling framework was used (pp. 1-2). | Selection was informative for a case audit but not representative. |
| 3 | Was the sample size adequate? | No | Only one facility was included. | No population-level precision or variability can be estimated. |
| 4 | Were the facility and setting described in detail? | Yes | Catchment area, staffing, location, flood history, and service context were described (pp. 1-2). | The case context was detailed. |
| 5 | Was analysis conducted with sufficient coverage of the facility? | Yes | Structural, non-structural, and emergency-management modules and item relevance were assessed (pp. 2-4). | The audit covered the main HSI domains within the facility. |
| 6 | Were valid methods used to identify preparedness/safety? | Yes | The WHO Hospital Safety Index was used with explicit scoring and a PHC-relevance assessment (pp. 2-3). | The HSI is standardized, although designed for hospitals. |
| 7 | Was preparedness measured in a standard, reliable way? | Unclear | Structured interviews, visual inspection, and HSI scoring were used, but assessor independence and inter-rater reliability were not reported (pp. 2-3). | Measurement reliability is uncertain. |
| 8 | Was statistical analysis appropriate? | Yes | Descriptive HSI indices and category scores were suitable for a single-facility audit (pp. 3-4). | Analysis matched the case-audit purpose. |
| 9 | Was response rate or missing information managed appropriately? | N/A | The unit of analysis was one facility; no conventional response rate applies. | Completeness depended on available interviews and documents rather than participant response. |

**Final narrative methodological judgment:** Major concerns for external validity, but not for the descriptive case objective. The study provides a detailed and operationally useful facility audit; it should not be generalized as evidence of prevalence across PHCs.

## S7F. JBI analytical cross-sectional appraisal of Fathoni et al. (2022)

### Fathoni et al. (2022)

**Design assessed: Quantitative correlative analytical observational study with a cross-sectional approach involving 120 nurses from 16 primary health centers, selected by quota sampling (60 emergency-department and 60 non-emergency-department nurses). The paper also reports intervention/control and pre-post results that cannot be reconciled with the stated design. Appraisal tool: JBI Checklist for Analytical Cross-Sectional Studies, applied to the cross-sectional associations used in this review.**

| **No.** | **Appraisal criterion** | **Final consensus judgment** | **Full-text evidence and location** | **Consensus rationale** |
| --- | --- | --- | --- | --- |
| 1 | Were the criteria for inclusion in the sample clearly defined? | Yes | The paper specifies active nurses working in emergency and non-emergency units of 16 primary health centers and lists inclusion requirements (Methods, pp. 2–3). | Eligibility was reported, although the non-probability quota method limits representativeness. |
| 2 | Were the study subjects and setting described in detail? | Yes | The report identifies 120 nurses from 16 primary health centers in Malang City and separates 60 emergency-department and 60 non-emergency-department nurses (Methods, pp. 2–3). | The setting and sample structure are described sufficiently for the cross-sectional analysis. |
| 3 | Was the exposure measured in a valid and reliable way? | Unclear | Knowledge, attitude, infrastructure, and self-control were measured by a 30-item disaster-preparedness questionnaire; validity and reliability coefficients are reported, but construct-specific validation procedures are only briefly described (p. 2). | Some measurement evidence is provided, but its scope and validation process cannot be fully verified. |
| 4 | Were objective, standard criteria used for measurement of the condition? | Unclear | The report describes questionnaire scoring and associations, but does not provide an external standard or independently verified criterion for preparedness/self-control status (pp. 2–3). | The measurement is questionnaire-based and the operational threshold is not fully documented. |
| 5 | Were confounding factors identified? | No | The analysis reports bivariate gamma correlations without a prespecified conceptual confounder framework (Results, pp. 2–3). | Potential demographic and organizational confounders were not explicitly identified. |
| 6 | Were strategies to deal with confounding factors stated? | No | No multivariable adjustment, stratified analysis, or other confounder-control strategy is reported for the cross-sectional associations (Results, pp. 2–3). | The reported associations are unadjusted. |
| 7 | Were the outcomes measured in a valid and reliable way? | Unclear | The paper reports questionnaire validity and reliability coefficients, but the outcome-specific validation and administration details are limited (p. 2). | Reliability evidence is present, but full outcome-validity evidence cannot be confirmed. |
| 8 | Was appropriate statistical analysis used? | Unclear | Gamma correlation is reported for the cross-sectional associations, but the paper also presents intervention/control and pre-post analyses that are inconsistent with the stated design and sampling (pp. 2–3). | The bivariate association test may fit ordinal cross-sectional data, but the full analytic report is internally inconsistent and lacks adjusted analysis. |

**Final narrative methodological judgment: Major methodological concerns. The stated cross-sectional design, quota sampling, limited construct-specific validation detail, absence of an explicit confounder framework or adjusted analysis, and contradictory intervention/control and pre-post reporting restrict confidence. Only the source-reported cross-sectional factors and preparedness indicators were used in the synthesis; no intervention effect was inferred.**

Final two-reviewer status: All 15 primary empirical PHC appraisal records were independently completed from the full text and confirmed by SNI and TR. All differences were resolved through source rechecking and consensus. No pending appraisal labels or provisional accuracy boundaries remain.

# Supplementary Tables S8–S9. Complete 74-publication study-by-component Health EDRM matrix

Every cell is populated. Y - [indicator phrase] means at least one source-derived determinant or indicator was identified. NR means not identified in the retained article text and is not evidence that the component was absent in the study setting.

## Supplementary Table S8. Policies, planning, human resources, financing, and information

| **No.** | **Study/source** | **Evidence role** | **PSL** | **PC** | **HR** | **FR** | **IKM** |
| --- | --- | --- | --- | --- | --- | --- | --- |
| 1 | Lamberti-Castronuovo et al. (2022) | Contextual evidence synthesis | Y — integrated PHC–H-EDRM strategy | Y — coordination and continuity planning | Y — multidisciplinary team | NR | Y — health information systems and telemedicine |
| 2 | Ward et al. (2024) | Contextual evidence synthesis | NR | Y — pre-disaster network coordination | Y — clinical leadership, teamwork, and staff support | Y — preparedness resource availability | NR |
| 3 | Simsek et al. (2024) | Contextual evidence synthesis | NR | Y — evacuation planning and provider roles | NR | NR | Y — geographic information and preparedness data |
| 4 | van der Heijden et al. (2022) | Contextual evidence synthesis | Y — water-emergency policy and standards | Y — stakeholder planning with water authorities and suppliers | NR | NR | NR |
| 5 | Almukhlifi et al. (2022) | Contextual evidence synthesis | NR | NR | Y — education and training, prior disaster experience, and employment status | NR | NR |
| 6 | Su et al. (2023) | Contextual evidence synthesis | NR | NR | Y — declarative and procedural knowledge from structured education | NR | NR |
| 7 | Alrowili et al. (2025) | Primary PHC synthesis | NR | NR | Y — technical knowledge, simulation, teamwork, emotional intelligence, and stress management | NR | NR |
| 8 | Dg Sirua et al. (2025) | Primary PHC synthesis | Y - nurses' policy-development and planning competency (pp. 1-4) | Y - planning, SOPs and multisector collaboration for PHC resilience (pp. 3-8) | Y - nurse disaster competencies, knowledge and training (pp. 1-8) | NR - no financial preparedness determinant was assessed | Y - understanding disaster risk and preparedness knowledge (pp. 3-6) |
| 9 | Derese et al. (2025) | Primary PHC synthesis | Y - implementation barriers and non-uniform application of cholera protocols and guidelines (pp. 8-9) | Y - written preparedness plans, trained rapid response teams, and coordination with sub-city health offices and other sectors; the plan was independently associated with preparedness (pp. 5-8) | Y - PHEM officers, training, rapid response teams, and staff-motivation barriers (pp. 5-9) | Y - inadequate dedicated emergency budget and financial support (pp. 8-10) | Y - posted case definitions, surveillance-data analysis for early detection, and functional timely disease reporting (p. 5) |
| 10 | Zarei et al. (2025) | Primary PHC synthesis | NR | Y — DART guideline usability and planning | Y — educational needs and organizational leadership | NR | Y — continuous needs assessment and health information |
| 11 | Lamberti-Castronuovo et al. (2024) | Primary PHC synthesis | NR | Y — incident plans, referral agreements, and transport arrangements | Y — multidisciplinary teams, surge staffing, and training | NR | Y — health information systems |
| 12 | Prakash et al. (2024) | Primary PHC synthesis | NR | Y — disaster mental-health preparedness planning | Y — disaster mental-health knowledge, attitudes, practices, psychological first-aid concepts, and clinical experience | NR | NR |
| 13 | Fathoni et al. (2022) | Primary PHC synthesis | NR | Y — health protocols | Y — protocol knowledge and staff self-control | NR | Y — disaster-preparedness questionnaire information |
| 14 | Lestari et al. (2022) | Primary PHC synthesis | Y — facility-safety governance and standards | Y — disaster committee and response plan | Y — training and committee capacity | Y — preparedness budgeting | NR |
| 15 | Litke et al. (2022) | Primary PHC synthesis | Y — primary-care resilience governance | NR | Y — employee resilience and organizational adaptation | NR | Y — communication and organizational information |
| 16 | Firdausi et al. (2021) | Primary PHC synthesis | Y - PAHO HSI standards and facility disaster-governance structures (pp. 1-4) | Y - disaster committee, response plan, activation/deactivation, referral and coordination procedures (pp. 3-8) | Y - staff responsibilities and periodic disaster training (pp. 3, 7-8) | NR - no explicit preparedness budget or financing mechanism was assessed | Y - hazard information, inspection/monitoring records and patient reporting (pp. 3-8) |
| 17 | Van Hoang et al. (2021) | Primary PHC synthesis | Y - regulations, leadership/governance, and ambiguous health-governance mechanisms (pp. 2, 6-8) | Y - emergency-operation activation; interagency coordination; police/military and private-sector collaboration (pp. 2-8) | Y - staff quantity/quality, allocation, training, volunteers, and workforce constraints (pp. 2, 4-8) | Y - funding sources, insurance, allowances, and inappropriate financing mechanisms (pp. 2, 7-8) | Y - surveillance, laboratory reporting, multiple reporting channels, and reporting burden (pp. 2-7) |
| 18 | Yari et al. (2021) | Primary PHC synthesis | Y — PHC safety standards | Y — response team and disaster plan | Y — continuous training | Y — preparedness financing | NR |
| 19 | Ndumbi et al. (2020) | Primary PHC synthesis | NR | Y — cholera protocols, patient transfer, and isolation | NR | NR | Y — outbreak information and reporting |
| 20 | Lapcevic et al. (2019) | Primary PHC synthesis | NR | Y — disaster plans | Y — staffing | Y — preparedness finance | Y — telecommunications |
| 21 | Hashikawa and Gold (2018) | Primary PHC synthesis | NR | Y — personal and clinic disaster plans | Y — leadership attitudes and attendance barriers | NR | Y — threat appraisal |
| 22 | Singh et al. (2026) | Contextual evidence synthesis | Y — preparedness standards and safety policy | NR | NR | NR | Y — preparedness assessment instruments and data |
| 23 | Ndembi et al. (2024) | Contextual evidence synthesis | Y — governance and harmonization of regional and global PPPR financing mechanisms | NR | NR | Y — sustainable and equitable financing for resilient health systems and access to medical countermeasures | NR |
| 24 | Awoyale et al. (2026) | Contextual evidence synthesis | NR | NR | Y — risk literacy and response capacity | NR | NR |
| 25 | Alfaifi and Hamaideh (2026) | Contextual evidence synthesis | NR | NR | Y — nurses’ disaster knowledge, training, and experience | NR | NR |
| 26 | Yadav et al. (2026) | Contextual evidence synthesis | Y — legal and accreditation frameworks | Y — institutional preparedness procedures | Y — workforce education and preparedness culture | Y — preparedness resources and financing | NR |
| 27 | Natali et al. (2025) | Contextual evidence synthesis | Y — multilevel governance and policy design | Y — policy coordination and implementation | NR | Y — recovery and resilience financing | NR |
| 28 | Rao et al. (2025) | Contextual evidence synthesis | NR | Y — operational planning and coordination | Y — ethical decision-making, triage, and training | NR | NR |
| 29 | Alrabie et al. (2025) | Contextual evidence synthesis | NR | Y — standard disaster protocols | Y — education, workshops, and disaster-field experience | NR | NR |
| 30 | Hauta et al. (2025) | Contextual evidence synthesis | NR | Y — institutional disaster plans and collaboration | Y — knowledge, attitudes, and emergency-management experience | NR | NR |
| 31 | Abebe et al. (2025) | Contextual evidence synthesis | NR | Y — flood evacuation and preparedness plans | NR | Y — resource allocation for flood resilience | NR |
| 32 | Hong et al. (2025) | Contextual evidence synthesis | NR | NR | NR | NR | NR |
| 33 | Tran et al. (2025) | Contextual evidence synthesis | NR | NR | Y — health-worker digital and data literacy | NR | Y — interoperable human–animal health data and early warning |
| 34 | Majambo et al. (2025) | Contextual evidence synthesis | NR | NR | NR | NR | NR |
| 35 | Cvetkovic et al. (2024) | Contextual evidence synthesis | Y — standards and policy compliance | Y — EMS organization and procedures | Y — staffing and continuing education | NR | NR |
| 36 | Lee and Lee (2024) | Contextual evidence synthesis | Y — public-policy effectiveness | NR | NR | NR | NR |
| 37 | Aggarwal et al. (2024) | Contextual evidence synthesis | NR | NR | NR | NR | NR |
| 38 | Tang (2024) | Contextual evidence synthesis | NR | NR | NR | NR | Y — machine-learning-supported modelling of airborne infection risk in healthcare facilities |
| 39 | Guha et al. (2025) | Contextual evidence synthesis | Y — national policy and governance | NR | Y — workforce adaptation | NR | NR |
| 40 | Elaidy et al. (2023) | Contextual evidence synthesis | NR | NR | Y — staff mental health, coping, and employment resilience | NR | NR |
| 41 | Noboa-Ramos et al. (2023) | Contextual evidence synthesis | NR | NR | NR | NR | NR |
| 42 | Mukherji et al. (2022) | Contextual evidence synthesis | NR | Y — governance, coordination, and risk-sharing in partnership-supported primary care | NR | NR | NR |
| 43 | Maghfiroh and Hanaoka (2022) | Contextual evidence synthesis | NR | NR | NR | NR | NR |
| 44 | Pawar et al. (2024) | Contextual evidence synthesis | NR | Y — domain-specific preparedness and coordination | Y — human resources and personnel protection | Y — financial preparedness | NR |
| 45 | Baker (2021) | Contextual evidence synthesis | NR | Y — Incident Command System | Y — disaster terminology, triage ethics, and decontamination | NR | Y — surveillance |
| 46 | Xiang et al. (2021) | Contextual evidence synthesis | Y — language-access policy | NR | Y — limited-English-proficiency and language capacity | NR | NR |
| 47 | George and Anilkumar (2021) | Contextual evidence synthesis | NR | Y — operational standard operating procedures | NR | NR | NR |
| 48 | Shiroya et al. (2021) | Contextual evidence synthesis | NR | NR | NR | NR | Y — medical records and HMIS |
| 49 | Palinkas et al. (2021) | Contextual evidence synthesis | NR | Y — search-and-rescue and blended pandemic–storm coordination | NR | NR | NR |
| 50 | Pourmohammadi et al. (2021) | Contextual evidence synthesis | NR | Y — functional preparedness plan | Y — rapid-response team | Y — facility insurance and financial protection | Y — hazard mapping |
| 51 | Li et al. (2021) | Contextual evidence synthesis | NR | Y — governance and emergency-team organization | Y — emergency-team formation | NR | Y — scientometric knowledge mapping |
| 52 | Wuthisuthimethawee et al. (2021) | Contextual evidence synthesis | Y — national standards embedded in the assessment tool | Y — mass-casualty and disaster plans | Y — health workforce and leadership | Y — emergency financing | Y — information systems |
| 53 | Ibrahim et al. (2021) | Contextual evidence synthesis | NR | NR | Y — health-system workforce resilience and familiarity | NR | NR |
| 54 | Gilmore et al. (2020) | Contextual evidence synthesis | NR | NR | NR | NR | NR |
| 55 | Oppong et al. (2020) | Contextual evidence synthesis | NR | NR | Y — infection-prevention workforce preparedness | NR | NR |
| 56 | Hierink et al. (2020) | Contextual evidence synthesis | NR | NR | NR | NR | Y — GIS, travel-time, and accessibility modelling |
| 57 | Brewer et al. (2020) | Contextual evidence synthesis | NR | NR | Y — trauma, triage, leadership, and psychological preparedness | NR | NR |
| 58 | Sharma and Sharma (2020) | Contextual evidence synthesis | NR | Y — critical-stabilization and isolation procedures | Y — staff retraining | NR | NR |
| 59 | Sultan et al. (2020) | Contextual evidence synthesis | NR | NR | Y — preparedness knowledge, confidence, willingness to work, and own/family safety considerations | NR | Y — self-rated knowledge and perceptions of risk and danger |
| 60 | Wagner et al. (2021) | Contextual evidence synthesis | NR | NR | Y — simulation-based pandemic and disaster readiness | NR | NR |
| 61 | Sheikhbardsiri et al. (2019) | Contextual evidence synthesis | NR | Y — operational exercise design and coordination | NR | NR | Y — scenario information and operational uncertainty |
| 62 | Ballard et al. (2022) | Contextual evidence synthesis | NR | NR | Y — trained, supervised, remunerated, and integrated community health worker workforce | NR | NR |
| 63 | Qari et al. (2019) | Contextual evidence synthesis | NR | Y — public-health preparedness-system coordination | Y — workforce capability | NR | Y — evaluation information |
| 64 | Thwala et al. (2018) | Contextual evidence synthesis | NR | Y — emergency obstetric and newborn-care coordination | Y — staffing | NR | NR |
| 65 | Sabegh et al. (2017) | Contextual evidence synthesis | NR | NR | Y — operational capacity in the healthcare supply chain | NR | NR |
| 66 | Lucchini et al. (2017) | Contextual evidence synthesis | NR | NR | NR | NR | Y — exposure assessment |
| 67 | Powell et al. (2016) | Contextual evidence synthesis | NR | NR | NR | NR | Y — dynamic threat and risk information |
| 68 | Bala et al. (2016) | Contextual evidence synthesis | NR | Y — integrated IT architecture for disaster-response coordination | NR | NR | Y — health-data integration and patient/information tracking |
| 69 | Sharifian et al. (2017) | Contextual evidence synthesis | Y - national policies, legislation, standards, organizational mission and vision (pp. 1-3) | Y - organizational framework, planning, coordination, command and surge-capacity strategies (pp. 2-3) | Y - training, education and resource-development strategies (pp. 2-3) | Y - disaster-relief funding and resource allocation (p. 3) | Y - information systems, GIS, databases and online connectivity (pp. 2-3) |
| 70 | Rahmati-Najarkolaei et al. (2017) | Contextual evidence synthesis | Y - HICS protocol, job descriptions and task hierarchy (pp. 1-3) | Y - incident command, disaster plan, coordination meetings and surge maneuver (pp. 1-3) | Y - staff knowledge, roles, training, call-back and competency gaps (pp. 2-5) | NR - no preparedness-financing determinant was reported | Y - information/communication unit, patient tracking and reporting functions (pp. 2-4) |
| 71 | Theron and Smith (2026) | Contextual evidence synthesis | Y - disaster-risk-reduction legislation, policy and adaptive governance (pp. 1, 4) | Y - multisectoral/district planning, coordinated roles and contingency planning (pp. 1, 3-5) | Y - primary-care-provider roles, staff access to plans and workforce capacity (pp. 3-5) | NR - no specific preparedness budget or financing mechanism was identified | Y - hazard/vulnerability assessment, mapping and sharing of local knowledge (pp. 2-5) |
| 72 | Adhikari et al. (2025) | Contextual evidence synthesis | Y - government-designated hub-satellite model and preparedness standards (pp. 1-3) | Y - disaster plans, committees, surge activation, evacuation and communication contacts (pp. 2-5) | Y - staffing numbers/ratios and early-warning/reporting-system training (pp. 3-5) | NR - no facility preparedness budget or financing mechanism was reported | Y - EWARS, infectious-disease reporting and communication lists (pp. 4-5) |
| 73 | Petrazzuoli et al. (2025) | Contextual evidence synthesis | Y - government support, guidance and health-system responsibilities (pp. 3-17) | Y - triage, adaptation to changing requirements, interprofessional coordination and resilience groups (pp. 3-17) | Y - PCP staffing, teamwork, workload and resilience-training needs (pp. 3-17) | NR - no specific financing mechanism or preparedness budget was evaluated | Y - information timeliness, changing guidance and remote-care information flows (pp. 3-17) |
| 74 | Paganini et al. (2016) | Contextual evidence synthesis | Y - hospital disaster plans and action/task cards (pp. 2-5) | Y - plan activation, command authority, roles and operational procedures (pp. 2-5) | Y - staff knowledge, prior training, role clarity and recall arrangements (pp. 2-7) | NR - no financial preparedness determinant was reported | Y - plan availability, action cards and bed-capacity information (pp. 2-5) |

## Supplementary Table S9. Risk communication, infrastructure/logistics, services, community capacities, and monitoring

| **No.** | **Study/source** | **Evidence role** | **RC** | **HIL** | **HRS** | **CC** | **ME** |
| --- | --- | --- | --- | --- | --- | --- | --- |
| 1 | Lamberti-Castronuovo et al. (2022) | Contextual evidence synthesis | NR | NR | Y — continuity of care | NR | NR |
| 2 | Ward et al. (2024) | Contextual evidence synthesis | NR | Y — medical products and technology | NR | NR | NR |
| 3 | Simsek et al. (2024) | Contextual evidence synthesis | NR | Y — access and evacuation infrastructure | NR | Y — vulnerable-population and family education | Y — preparedness assessment |
| 4 | van der Heijden et al. (2022) | Contextual evidence synthesis | NR | Y — basic WASH and emergency water infrastructure | NR | Y — water-authority and supplier engagement | NR |
| 5 | Almukhlifi et al. (2022) | Contextual evidence synthesis | NR | NR | NR | NR | NR |
| 6 | Su et al. (2023) | Contextual evidence synthesis | NR | NR | Y — systematic educational and mental-health support | NR | NR |
| 7 | Alrowili et al. (2025) | Primary PHC synthesis | NR | NR | NR | NR | NR |
| 8 | Dg Sirua et al. (2025) | Primary PHC synthesis | NR - no explicit risk-communication indicator was assessed | Y - structural resilience and facility functionality in the resilience framework (pp. 2-4) | Y - continued accessibility of essential PHC services during disasters (pp. 1, 4) | Y - collaboration with local government, health institutions, communities and NGOs (pp. 1, 7-8) | NR - no explicit monitoring/evaluation system was reported |
| 9 | Derese et al. (2025) | Primary PHC synthesis | NR - the study explicitly states that community risk-communication strategies were not evaluated | Y - ORS, intravenous fluids, antibiotics, safe water, latrines, waste disposal, handwashing facilities, and PPE (pp. 5-7) | Y - cholera case-management readiness and treatment supplies (pp. 5-7) | NR - community-based preparedness and engagement were not evaluated | Y - facility preparedness was scored using a standardized 14-item WHO/national checklist, with sensitivity analyses at 70%, 75%, and 80% thresholds (pp. 5-7) |
| 10 | Zarei et al. (2025) | Primary PHC synthesis | Y — early warning and risk communication | NR | NR | Y — cultural adaptation and community relevance | NR |
| 11 | Lamberti-Castronuovo et al. (2024) | Primary PHC synthesis | Y — staff and community communication | Y — PPE, stocks, and utilities | Y — service integration and continuity | Y — community engagement | Y — operational preparedness checklist and repeated assessment |
| 12 | Prakash et al. (2024) | Primary PHC synthesis | NR | NR | Y — preparedness knowledge concerning psychosocial triage and disaster mental-health response | NR | NR |
| 13 | Fathoni et al. (2022) | Primary PHC synthesis | NR | NR | NR | NR | Y — DPQ-based preparedness assessment |
| 14 | Lestari et al. (2022) | Primary PHC synthesis | NR | Y — structural and non-structural safety, water, fuel, medicines, and equipment | NR | NR | Y — Hospital Safety Index assessment |
| 15 | Litke et al. (2022) | Primary PHC synthesis | NR | Y — backup storage, masks, medicines, and disinfectants | NR | NR | NR |
| 16 | Firdausi et al. (2021) | Primary PHC synthesis | Y - warning systems, internal communication and community education (pp. 6-8) | Y - structural/nonstructural safety, water, power, equipment, medicines and PPE stocks (pp. 3-8) | Y - essential PHC, maternal, immunization, mental-health and telemedicine services (pp. 7-8) | Y - coordination with neighborhood/community leaders and community education (pp. 7-8) | Y - HSI assessment, routine testing, preventive maintenance and monitoring records (pp. 3-8) |
| 17 | Van Hoang et al. (2021) | Primary PHC synthesis | Y - public information, alerts, warnings, notifications, and communication channels (pp. 1, 4-5) | Y - infrastructure, equipment, consumables, testing and diagnostic capacity (pp. 2, 6) | Y - isolation, diagnosis, prevention-treatment collaboration, laboratory and case-management services (pp. 1, 4-6) | Y - community partnerships, local authorities, mass organizations and volunteer involvement (pp. 2, 4-6) | Y - evaluation of emergency operations, surveillance meetings and volunteer-activity evaluation (pp. 1, 4) |
| 18 | Yari et al. (2021) | Primary PHC synthesis | NR | Y — structural, non-structural, and functional safety; equipment, water, and food | Y — service functionality | Y — community-related safety capacity | Y — Hospital Safety Index assessment |
| 19 | Ndumbi et al. (2020) | Primary PHC synthesis | NR | Y — WASH, chlorine, sterilization, waste, ORS, and emergency fluids | Y — cholera response services | NR | Y — facility preparedness assessment |
| 20 | Lapcevic et al. (2019) | Primary PHC synthesis | NR | Y — structural integrity, utilities, and laboratory equipment | NR | NR | Y — Hospital Safety Index and 72-hour operational assessment |
| 21 | Hashikawa and Gold (2018) | Primary PHC synthesis | NR | NR | NR | NR | Y — exercises and preparedness assessment |
| 22 | Singh et al. (2026) | Contextual evidence synthesis | NR | Y — structural, non-structural, and functional safety | NR | NR | Y — comparative preparedness assessment |
| 23 | Ndembi et al. (2024) | Contextual evidence synthesis | NR | NR | NR | NR | NR |
| 24 | Awoyale et al. (2026) | Contextual evidence synthesis | Y — risk communication | Y — WASH, transport, and hazard-exposed infrastructure | NR | Y — interagency and community coordination | NR |
| 25 | Alfaifi and Hamaideh (2026) | Contextual evidence synthesis | NR | NR | NR | NR | NR |
| 26 | Yadav et al. (2026) | Contextual evidence synthesis | NR | Y — facility infrastructure | Y — emergency service readiness | NR | Y — accreditation and compliance assessment |
| 27 | Natali et al. (2025) | Contextual evidence synthesis | NR | NR | NR | NR | NR |
| 28 | Rao et al. (2025) | Contextual evidence synthesis | NR | Y — resources for austere response | NR | Y — coordination with government and humanitarian actors | NR |
| 29 | Alrabie et al. (2025) | Contextual evidence synthesis | NR | Y — medical resources and logistics | NR | NR | NR |
| 30 | Hauta et al. (2025) | Contextual evidence synthesis | NR | NR | NR | NR | Y — preparedness assessment |
| 31 | Abebe et al. (2025) | Contextual evidence synthesis | NR | Y — backup generators and flood-resilient infrastructure | NR | NR | NR |
| 32 | Hong et al. (2025) | Contextual evidence synthesis | Y — disaster risk perception and communication | Y — material preparedness resources and supplies | NR | NR | NR |
| 33 | Tran et al. (2025) | Contextual evidence synthesis | NR | NR | NR | NR | NR |
| 34 | Majambo et al. (2025) | Contextual evidence synthesis | NR | Y — emergency medicines and equipment in dental practices | Y — dental-practice readiness for medical emergencies | NR | NR |
| 35 | Cvetkovic et al. (2024) | Contextual evidence synthesis | NR | Y — equipment and vehicles | Y — emergency response services | NR | Y — response-time and performance assessment |
| 36 | Lee and Lee (2024) | Contextual evidence synthesis | NR | Y — spatial distribution and robustness of health infrastructure | NR | NR | NR |
| 37 | Aggarwal et al. (2024) | Contextual evidence synthesis | NR | NR | Y — stepped-care and service-delivery response | Y — rural access and community needs | NR |
| 38 | Tang (2024) | Contextual evidence synthesis | NR | NR | NR | NR | NR |
| 39 | Guha et al. (2025) | Contextual evidence synthesis | NR | NR | Y — continuity of essential health services | Y — community- and patient-level continuity measures | NR |
| 40 | Elaidy et al. (2023) | Contextual evidence synthesis | NR | Y — availability of PPE | NR | NR | NR |
| 41 | Noboa-Ramos et al. (2023) | Contextual evidence synthesis | NR | Y — hurricane-related disruptions to power, water, communications, and critical supplies | Y — effects on healthcare delivery and organizational recovery | Y — coordination between healthcare and social organizations and attention to patient social needs | NR |
| 42 | Mukherji et al. (2022) | Contextual evidence synthesis | NR | NR | NR | Y — community-oriented primary-care delivery through partnership arrangements | NR |
| 43 | Maghfiroh and Hanaoka (2022) | Contextual evidence synthesis | NR | Y — mobile-clinic logistics and strategic location | Y — mobile medical-service delivery | NR | NR |
| 44 | Pawar et al. (2024) | Contextual evidence synthesis | Y — communication | Y — logistics and evacuation resources | NR | NR | NR |
| 45 | Baker (2021) | Contextual evidence synthesis | NR | Y — access to critical resources | Y — nursing disaster-response readiness | NR | NR |
| 46 | Xiang et al. (2021) | Contextual evidence synthesis | NR | NR | NR | Y — community language access | NR |
| 47 | George and Anilkumar (2021) | Contextual evidence synthesis | Y — coordination and communication indicators | NR | NR | NR | NR |
| 48 | Shiroya et al. (2021) | Contextual evidence synthesis | NR | Y — oxygen and facility readiness | Y — continuity and integration of NCD services | NR | NR |
| 49 | Palinkas et al. (2021) | Contextual evidence synthesis | NR | NR | Y — rapid preparedness-needs and resource response | Y — community resource assessment | NR |
| 50 | Pourmohammadi et al. (2021) | Contextual evidence synthesis | NR | NR | NR | NR | Y — facility preparedness assessment |
| 51 | Li et al. (2021) | Contextual evidence synthesis | NR | Y — adequacy of physical resources | NR | NR | Y — resilience assessment |
| 52 | Wuthisuthimethawee et al. (2021) | Contextual evidence synthesis | NR | Y — medical products and technology | NR | Y — community and pediatric considerations | NR |
| 53 | Ibrahim et al. (2021) | Contextual evidence synthesis | NR | NR | NR | NR | NR |
| 54 | Gilmore et al. (2020) | Contextual evidence synthesis | Y — community communication and social mobilization for infectious-disease prevention and control | NR | NR | Y — community actors, participatory engagement, local ownership, and health-system linkages | NR |
| 55 | Oppong et al. (2020) | Contextual evidence synthesis | NR | Y — PPE and sterile equipment | Y — infection-prevention and control service readiness | NR | NR |
| 56 | Hierink et al. (2020) | Contextual evidence synthesis | NR | NR | NR | NR | NR |
| 57 | Brewer et al. (2020) | Contextual evidence synthesis | Y — regional and rural emergency communication | NR | Y — emergency-department response readiness | NR | NR |
| 58 | Sharma and Sharma (2020) | Contextual evidence synthesis | NR | Y — isolation rooms and medication stock | Y — critical-stabilization services | Y — community health-centre linkage | NR |
| 59 | Sultan et al. (2020) | Contextual evidence synthesis | NR | NR | NR | NR | NR |
| 60 | Wagner et al. (2021) | Contextual evidence synthesis | NR | NR | NR | NR | NR |
| 61 | Sheikhbardsiri et al. (2019) | Contextual evidence synthesis | Y — communication during the exercise | Y — disaster-response resources | NR | NR | Y — exercise-based preparedness evaluation |
| 62 | Ballard et al. (2022) | Contextual evidence synthesis | Y — household risk communication and trusted community education | Y — PPE, essential commodities, and supply-chain support | Y — community-based essential services, surveillance, testing referral, and home-based care | Y — community engagement and community–health-system linkages | Y — community health worker data systems, performance monitoring, and evaluation |
| 63 | Qari et al. (2019) | Contextual evidence synthesis | NR | NR | NR | Y — system and community partners | Y — performance metrics |
| 64 | Thwala et al. (2018) | Contextual evidence synthesis | NR | Y — facility equipment and supplies | Y — emergency obstetric services | NR | NR |
| 65 | Sabegh et al. (2017) | Contextual evidence synthesis | NR | Y — green disaster-response supply chain | NR | NR | Y — multi-objective performance optimization |
| 66 | Lucchini et al. (2017) | Contextual evidence synthesis | NR | NR | Y — lifetime health care | Y — affected populations | Y — systematic preparedness and long-term monitoring |
| 67 | Powell et al. (2016) | Contextual evidence synthesis | NR | NR | NR | NR | Y — auditable risk identification and assessment |
| 68 | Bala et al. (2016) | Contextual evidence synthesis | Y — IT-enabled emergency communication | NR | NR | NR | NR |
| 69 | Sharifian et al. (2017) | Contextual evidence synthesis | Y - communication planning and early-warning systems (pp. 2-3) | Y - infrastructure strengthening and material-resource development (pp. 2-3) | NR - no specific continuity or delivery indicator for health services was identified | Y - community education, empowerment, civil defense and community-based preparedness (p. 3) | Y - vulnerability/risk assessment, evaluation and exercises (pp. 2-3) |
| 70 | Rahmati-Najarkolaei et al. (2017) | Contextual evidence synthesis | NR - public/community risk communication was not reported; internal communication was coded under IKM/PC | Y - logistics, transport, ambulance, medicines, equipment and security functions (pp. 2-4) | Y - triage, patient prioritization, rapid discharge and patient tracking (pp. 3-4) | NR - no community-capacity indicator was reported | Y - maneuver checklist, preparedness assessment and post-maneuver review/reporting (pp. 2-4) |
| 71 | Theron and Smith (2026) | Contextual evidence synthesis | Y - early warnings and community-tailored risk communication (pp. 4-5) | Y - essential supplies, infrastructure vulnerability and utility continuity (pp. 2-4) | Y - continuity of PHC and health-service delivery during climate hazards (pp. 1-5) | Y - community-based risk assessment, participation and resilience building (pp. 1-5) | Y - formal monitoring/evaluation of DRR plans and lessons learned (pp. 3-4) |
| 72 | Adhikari et al. (2025) | Contextual evidence synthesis | Y - early-warning/reporting arrangements and external communication contacts (pp. 4-5) | Y - structural assessment, power, oxygen, fuel, medicines, laboratory supplies and transport (pp. 4-5) | Y - triage, emergency care, blood bank, reproductive services and outbreak testing (pp. 4-5) | NR - no community-participation or community-capacity process was reported | Y - structural/nonstructural assessment, minimal-service standards and quality-improvement monitoring (pp. 4-5) |
| 73 | Petrazzuoli et al. (2025) | Contextual evidence synthesis | Y - communication problems between levels and communication of clinical/public guidance (pp. 3-17) | Y - PPE, medicines, workplace infrastructure, transport and telehealth (pp. 3-17) | Y - triage, remote care, palliative care and access to medicines/services (pp. 3-17) | Y - community volunteers and community resilience groups (pp. 4-16) | NR - no explicit preparedness monitoring/evaluation system was reported |
| 74 | Paganini et al. (2016) | Contextual evidence synthesis | NR - public/community risk communication was not reported; internal recall was coded under PC/IKM | Y - surge-bed information and arrangements to summon additional personnel (pp. 3-5) | Y - ED disaster response, patient flow and surge procedures (pp. 2-5) | NR - no community-capacity indicator was reported | Y - periodic training, testing and real-time assessment of plan knowledge (pp. 4-7) |

# Supplementary Table S10. Study-level coding-basis traceability

| **No.** | **Study/source** | **Manuscript ref.** | **Evidence role** | **Source-derived coding basis** | **Mapped component codes** |
| --- | --- | --- | --- | --- | --- |
| 1 | Lamberti-Castronuovo et al. (2022) | 3 | Contextual evidence synthesis | PSL: integrated PHC–H-EDRM strategy; PC: coordination and continuity planning; HR: multidisciplinary team; IKM: health information systems and telemedicine; HRS: continuity of care | PSL; PC; HR; IKM; HRS |
| 2 | Ward et al. (2024) | 4 | Contextual evidence synthesis | PC: pre-disaster network coordination; HR: clinical leadership, teamwork, and staff support; FR: preparedness resource availability; HIL: medical products and technology | PC; HR; FR; HIL |
| 3 | Simsek et al. (2024) | 5 | Contextual evidence synthesis | PC: evacuation planning and provider roles; IKM: geographic information and preparedness data; HIL: access and evacuation infrastructure; CC: vulnerable-population and family education; ME: preparedness assessment | PC; IKM; HIL; CC; ME |
| 4 | van der Heijden et al. (2022) | 6 | Contextual evidence synthesis | PSL: water-emergency policy and standards; PC: stakeholder planning with water authorities and suppliers; HIL: basic WASH and emergency water infrastructure; CC: water-authority and supplier engagement | PSL; PC; HIL; CC |
| 5 | Almukhlifi et al. (2022) | 7 | Contextual evidence synthesis | HR: education and training, prior disaster experience, and employment status | HR |
| 6 | Su et al. (2023) | 8 | Contextual evidence synthesis | HR: declarative and procedural knowledge from structured education; HRS: systematic educational and mental-health support | HR; HRS |
| 7 | Alrowili et al. (2025) | 13 | Primary PHC synthesis | HR: technical knowledge, simulation, teamwork, emotional intelligence, and stress management | HR |
| 8 | Dg Sirua et al. (2025) | 14 | Primary PHC synthesis | Policy/planning competence, disaster knowledge and training, structural resilience, continuity of PHC services and collaboration with communities and other organizations. | PSL; PC; HR; IKM; HIL; HRS; CC |
| 9 | Derese et al. (2025) | 15 | Primary PHC synthesis | Implementation of protocols/guidelines; emergency plans and rapid response teams; trained PHEM staff and motivation; emergency financing; surveillance and reporting; WASH, PPE, medicines and case-management readiness; multisectoral coordination; standardized facility preparedness scoring. | PSL; PC; HR; FR; IKM; HIL; HRS; ME |
| 10 | Zarei et al. (2025) | 16 | Primary PHC synthesis | PC: DART guideline usability and planning; HR: educational needs and organizational leadership; IKM: continuous needs assessment and health information; RC: early warning and risk communication; CC: cultural adaptation and community relevance | PC; HR; IKM; RC; CC |
| 11 | Lamberti-Castronuovo et al. (2024) | 17 | Primary PHC synthesis | PC: incident plans, referral agreements, and transport arrangements; HR: multidisciplinary teams, surge staffing, and training; IKM: health information systems; RC: staff and community communication; HIL: PPE, stocks, and utilities; HRS: service integration and continuity; CC: community engagement; ME: operational preparedness checklist and repeated assessment | PC; HR; IKM; RC; HIL; HRS; CC; ME |
| 12 | Prakash et al. (2024) | 18 | Primary PHC synthesis | PC: disaster mental-health preparedness planning; HR: disaster mental-health knowledge, attitudes, practices, psychological first-aid concepts, and clinical experience; HRS: preparedness knowledge concerning psychosocial triage and disaster mental-health response. | PC; HR; HRS |
| 13 | Fathoni et al. (2022) | 19 | Primary PHC synthesis | PC: health protocols; HR: protocol knowledge and staff self-control; IKM: disaster-preparedness questionnaire information; ME: DPQ-based preparedness assessment | PC; HR; IKM; ME |
| 14 | Lestari et al. (2022) | 20 | Primary PHC synthesis | PSL: facility-safety governance and standards; PC: disaster committee and response plan; HR: training and committee capacity; FR: preparedness budgeting; HIL: structural and non-structural safety, water, fuel, medicines, and equipment; ME: Hospital Safety Index assessment | PSL; PC; HR; FR; HIL; ME |
| 15 | Litke et al. (2022) | 21 | Primary PHC synthesis | PSL: primary-care resilience governance; HR: employee resilience and organizational adaptation; IKM: communication and organizational information; HIL: backup storage, masks, medicines, and disinfectants | PSL; HR; IKM; HIL |
| 16 | Firdausi et al. (2021) | 22 | Primary PHC synthesis | Governance, plans, staffing, information, warning and communication, utilities, stocks, essential services, community coordination and monitoring. | PSL; PC; HR; IKM; RC; HIL; HRS; CC; ME |
| 17 | Van Hoang et al. (2021) | 23 | Primary PHC synthesis | Facility preparedness checklist, interviews and focus groups identified preparedness planning, staffing, financing, surveillance, communication, infrastructure, service delivery, community partnerships and evaluation. | PSL; PC; HR; FR; IKM; RC; HIL; HRS; CC; ME |
| 18 | Yari et al. (2021) | 24 | Primary PHC synthesis | PSL: PHC safety standards; PC: response team and disaster plan; HR: continuous training; FR: preparedness financing; HIL: structural, non-structural, and functional safety; equipment, water, and food; HRS: service functionality; CC: community-related safety capacity; ME: Hospital Safety Index assessment | PSL; PC; HR; FR; HIL; HRS; CC; ME |
| 19 | Ndumbi et al. (2020) | 25 | Primary PHC synthesis | PC: cholera protocols, patient transfer, and isolation; IKM: outbreak information and reporting; HIL: WASH, chlorine, sterilization, waste, ORS, and emergency fluids; HRS: cholera response services; ME: facility preparedness assessment | PC; IKM; HIL; HRS; ME |
| 20 | Lapcevic et al. (2019) | 26 | Primary PHC synthesis | PC: disaster plans; HR: staffing; FR: preparedness finance; IKM: telecommunications; HIL: structural integrity, utilities, and laboratory equipment; ME: Hospital Safety Index and 72-hour operational assessment | PC; HR; FR; IKM; HIL; ME |
| 21 | Hashikawa and Gold (2018) | 27 | Primary PHC synthesis | PC: personal and clinic disaster plans; HR: leadership attitudes and attendance barriers; IKM: threat appraisal; ME: exercises and preparedness assessment | PC; HR; IKM; ME |
| 22 | Singh et al. (2026) | 29 | Contextual evidence synthesis | PSL: preparedness standards and safety policy; IKM: preparedness assessment instruments and data; HIL: structural, non-structural, and functional safety; ME: comparative preparedness assessment | PSL; IKM; HIL; ME |
| 23 | Ndembi et al. (2024) | 30 | Contextual evidence synthesis | PSL: governance and harmonization of regional and global pandemic prevention, preparedness and response (PPPR) financing mechanisms; FR: sustainable and equitable financing for resilient health systems and access to medical countermeasures. | PSL; FR |
| 24 | Awoyale et al. (2026) | 31 | Contextual evidence synthesis | HR: risk literacy and response capacity; RC: risk communication; HIL: WASH, transport, and hazard-exposed infrastructure; CC: interagency and community coordination | HR; RC; HIL; CC |
| 25 | Alfaifi and Hamaideh (2026) | 32 | Contextual evidence synthesis | HR: nurses’ disaster knowledge, training, and experience | HR |
| 26 | Yadav et al. (2026) | 33 | Contextual evidence synthesis | PSL: legal and accreditation frameworks; PC: institutional preparedness procedures; HR: workforce education and preparedness culture; FR: preparedness resources and financing; HIL: facility infrastructure; HRS: emergency service readiness; ME: accreditation and compliance assessment | PSL; PC; HR; FR; HIL; HRS; ME |
| 27 | Natali et al. (2025) | 34 | Contextual evidence synthesis | PSL: multilevel governance and policy design; PC: policy coordination and implementation; FR: recovery and resilience financing | PSL; PC; FR |
| 28 | Rao et al. (2025) | 35 | Contextual evidence synthesis | PC: operational planning and coordination; HR: ethical decision-making, triage, and training; HIL: resources for austere response; CC: coordination with government and humanitarian actors | PC; HR; HIL; CC |
| 29 | Alrabie et al. (2025) | 36 | Contextual evidence synthesis | PC: standard disaster protocols; HR: education, workshops, and disaster-field experience; HIL: medical resources and logistics | PC; HR; HIL |
| 30 | Hauta et al. (2025) | 37 | Contextual evidence synthesis | PC: institutional disaster plans and collaboration; HR: knowledge, attitudes, and emergency-management experience; ME: preparedness assessment | PC; HR; ME |
| 31 | Abebe et al. (2025) | 38 | Contextual evidence synthesis | PC: flood evacuation and preparedness plans; FR: resource allocation for flood resilience; HIL: backup generators and flood-resilient infrastructure | PC; FR; HIL |
| 32 | Hong et al. (2025) | 39 | Contextual evidence synthesis | RC: disaster risk perception and communication; HIL: material preparedness resources and supplies | RC; HIL |
| 33 | Tran et al. (2025) | 40 | Contextual evidence synthesis | HR: health-worker digital and data literacy; IKM: interoperable human–animal health data and early warning | HR; IKM |
| 34 | Majambo et al. (2025) | 41 | Contextual evidence synthesis | HIL: emergency medicines and equipment in dental practices; HRS: dental-practice readiness for medical emergencies. | HIL; HRS |
| 35 | Cvetkovic et al. (2024) | 42 | Contextual evidence synthesis | PSL: standards and policy compliance; PC: EMS organization and procedures; HR: staffing and continuing education; HIL: equipment and vehicles; HRS: emergency response services; ME: response-time and performance assessment | PSL; PC; HR; HIL; HRS; ME |
| 36 | Lee and Lee (2024) | 43 | Contextual evidence synthesis | PSL: public-policy effectiveness; HIL: spatial distribution and robustness of health infrastructure | PSL; HIL |
| 37 | Aggarwal et al. (2024) | 44 | Contextual evidence synthesis | HRS: stepped-care and service-delivery response; CC: rural access and community needs | HRS; CC |
| 38 | Tang (2024) | 45 | Contextual evidence synthesis | IKM: machine-learning-supported modelling of airborne infection risk in healthcare facilities. | IKM |
| 39 | Guha et al. (2025) | 46 | Contextual evidence synthesis | PSL: national policy and governance; HR: workforce adaptation; HRS: continuity of essential health services; CC: community- and patient-level continuity measures | PSL; HR; HRS; CC |
| 40 | Elaidy et al. (2023) | 47 | Contextual evidence synthesis | HR: staff mental health, coping, and employment resilience; HIL: availability of PPE | HR; HIL |
| 41 | Noboa-Ramos et al. (2023) | 48 | Contextual evidence synthesis | HIL: hurricane-related disruptions to power, water, communications, and critical supplies; HRS: effects on healthcare delivery and organizational recovery; CC: coordination between healthcare and social organizations and attention to patient social needs. | HIL; HRS; CC |
| 42 | Mukherji et al. (2022) | 49 | Contextual evidence synthesis | PC: governance, coordination, and risk-sharing arrangements in public-private partnership-supported primary care; CC: community-oriented primary-care service delivery through partnership arrangements. | PC; CC |
| 43 | Maghfiroh and Hanaoka (2022) | 50 | Contextual evidence synthesis | HIL: mobile-clinic logistics and strategic location; HRS: mobile medical-service delivery | HIL; HRS |
| 44 | Pawar et al. (2024) | 51 | Contextual evidence synthesis | PC: domain-specific preparedness and coordination; HR: human resources and personnel protection; FR: financial preparedness; RC: communication; HIL: logistics and evacuation resources | PC; HR; FR; RC; HIL |
| 45 | Baker (2021) | 52 | Contextual evidence synthesis | PC: Incident Command System; HR: disaster terminology, triage ethics, and decontamination; IKM: surveillance; HIL: access to critical resources; HRS: nursing disaster-response readiness | PC; HR; IKM; HIL; HRS |
| 46 | Xiang et al. (2021) | 53 | Contextual evidence synthesis | PSL: language-access policy; HR: limited-English-proficiency and language capacity; CC: community language access | PSL; HR; CC |
| 47 | George and Anilkumar (2021) | 54 | Contextual evidence synthesis | PC: operational standard operating procedures; RC: coordination and communication indicators | PC; RC |
| 48 | Shiroya et al. (2021) | 55 | Contextual evidence synthesis | IKM: medical records and HMIS; HIL: oxygen and facility readiness; HRS: continuity and integration of NCD services | IKM; HIL; HRS |
| 49 | Palinkas et al. (2021) | 56 | Contextual evidence synthesis | PC: search-and-rescue and blended pandemic–storm coordination; HRS: rapid preparedness-needs and resource response; CC: community resource assessment | PC; HRS; CC |
| 50 | Pourmohammadi et al. (2021) | 57 | Contextual evidence synthesis | PC: functional preparedness plan; HR: rapid-response team; FR: facility insurance and financial protection; IKM: hazard mapping; ME: facility preparedness assessment | PC; HR; FR; IKM; ME |
| 51 | Li et al. (2021) | 58 | Contextual evidence synthesis | PC: governance and emergency-team organization; HR: emergency-team formation; IKM: scientometric knowledge mapping; HIL: adequacy of physical resources; ME: resilience assessment | PC; HR; IKM; HIL; ME |
| 52 | Wuthisuthimethawee et al. (2021) | 59 | Contextual evidence synthesis | PSL: national standards embedded in the assessment tool; PC: mass-casualty and disaster plans; HR: health workforce and leadership; FR: emergency financing; IKM: information systems; HIL: medical products and technology; CC: community and pediatric considerations | PSL; PC; HR; FR; IKM; HIL; CC |
| 53 | Ibrahim et al. (2021) | 60 | Contextual evidence synthesis | HR: health-system workforce resilience and familiarity | HR |
| 54 | Gilmore et al. (2020) | 61 | Contextual evidence synthesis | RC: community communication and social-mobilization approaches for infectious-disease prevention and control; CC: community actors, participatory engagement, local ownership, and linkages with health-system stakeholders. | RC; CC |
| 55 | Oppong et al. (2020) | 62 | Contextual evidence synthesis | HR: infection-prevention workforce preparedness; HIL: PPE and sterile equipment; HRS: infection-prevention and control service readiness | HR; HIL; HRS |
| 56 | Hierink et al. (2020) | 63 | Contextual evidence synthesis | IKM: GIS, travel-time, and accessibility modelling | IKM |
| 57 | Brewer et al. (2020) | 64 | Contextual evidence synthesis | HR: trauma, triage, leadership, and psychological preparedness; RC: regional and rural emergency communication; HRS: emergency-department response readiness | HR; RC; HRS |
| 58 | Sharma and Sharma (2020) | 65 | Contextual evidence synthesis | PC: critical-stabilization and isolation procedures; HR: staff retraining; HIL: isolation rooms and medication stock; HRS: critical-stabilization services; CC: community health-centre linkage | PC; HR; HIL; HRS; CC |
| 59 | Sultan et al. (2020) | 66 | Contextual evidence synthesis | HR: preparedness knowledge, confidence, willingness to work, and own/family safety considerations; IKM: self-rated knowledge and perceptions of risk and danger. | HR; IKM |
| 60 | Wagner et al. (2021) | 67 | Contextual evidence synthesis | HR: simulation-based pandemic and disaster readiness | HR |
| 61 | Sheikhbardsiri et al. (2019) | 68 | Contextual evidence synthesis | PC: operational exercise design and coordination; IKM: scenario information and operational uncertainty; RC: communication during the exercise; HIL: disaster-response resources; ME: exercise-based preparedness evaluation. | PC; IKM; RC; HIL; ME |
| 62 | Ballard et al. (2022) | 69 | Contextual evidence synthesis | HR: trained, supervised, remunerated, and integrated community health worker workforce; RC: household risk communication and trusted community education; HIL: personal protective equipment, essential commodities, and supply-chain support; HRS: maintenance of community-based essential health services, surveillance, testing referral, and home-based care; CC: community engagement and links between communities and health systems; ME: community health worker data systems, performance monitoring, and evaluation. | HR; RC; HIL; HRS; CC; ME |
| 63 | Qari et al. (2019) | 70 | Contextual evidence synthesis | PC: public-health preparedness-system coordination; HR: workforce capability; IKM: evaluation information; CC: system and community partners; ME: performance metrics | PC; HR; IKM; CC; ME |
| 64 | Thwala et al. (2018) | 71 | Contextual evidence synthesis | PC: emergency obstetric and newborn-care coordination; HR: staffing; HIL: facility equipment and supplies; HRS: emergency obstetric services | PC; HR; HIL; HRS |
| 65 | Sabegh et al. (2017) | 72 | Contextual evidence synthesis | HR: operational capacity in the healthcare supply chain; HIL: green disaster-response supply chain; ME: multi-objective performance optimization | HR; HIL; ME |
| 66 | Lucchini et al. (2017) | 73 | Contextual evidence synthesis | IKM: exposure assessment; HRS: lifetime health care; CC: affected populations; ME: systematic preparedness and long-term monitoring | IKM; HRS; CC; ME |
| 67 | Powell et al. (2016) | 74 | Contextual evidence synthesis | IKM: dynamic threat and risk information; ME: auditable risk identification and assessment | IKM; ME |
| 68 | Bala et al. (2016) | 75 | Contextual evidence synthesis | PC: integrated information-technology architecture for disaster-response coordination; IKM: health-data integration and patient/information tracking; RC: information-technology-enabled emergency communication. | PC; IKM; RC |
| 69 | Sharifian et al. (2017) | 76 | Contextual evidence synthesis | Policy, planning, workforce development, financing, information, early warning, infrastructure, community education and evaluation. | PSL; PC; HR; FR; IKM; RC; HIL; CC; ME |
| 70 | Rahmati-Najarkolaei et al. (2017) | 77 | Contextual evidence synthesis | Incident command, roles, staff training, internal communication, logistics, triage, patient tracking and exercise evaluation. | PSL; PC; HR; IKM; HIL; HRS; ME |
| 71 | Theron and Smith (2026) | 78 | Contextual evidence synthesis | Risk governance, multisectoral planning, workforce capacity, hazard assessment, early warning, utilities, service continuity, community participation and evaluation. | PSL; PC; HR; IKM; RC; HIL; HRS; CC; ME |
| 72 | Adhikari et al. (2025) | 79 | Contextual evidence synthesis | Plans, committees, staffing, reporting systems, communication contacts, infrastructure, utilities, supplies, triage, essential services and monitoring. | PSL; PC; HR; IKM; RC; HIL; HRS; ME |
| 73 | Petrazzuoli et al. (2025) | 80 | Contextual evidence synthesis | Guidance, coordination, workforce resilience, information, communication, PPE and telehealth, service adaptation and community resilience groups. | PSL; PC; HR; IKM; RC; HIL; HRS; CC |
| 74 | Paganini et al. (2016) | 81 | Contextual evidence synthesis | Disaster plans, activation, command authority, staff roles and recall, information, surge arrangements, service response and periodic testing. | PSL; PC; HR; IKM; HIL; HRS; ME |

# Supplementary Tables S11–S12. Final component frequencies and documentation limitations after the retrieval and Q1–Q4 filter audits

## Supplementary Table S11. Comparative component frequencies after the retrieval and Q1–Q4 filter audits

| **Component** | **Primary PHC n/15 (%)** | **Contextual n/59 (%)** | **All n/74 (%)** | **Difference (pp)** | **Interpretation** |
| --- | --- | --- | --- | --- | --- |
| Human resources | 14/15 (93.3%) | 36/59 (61.0%) | 50/74 (67.6%) | +32.3 | Reported more frequently in PHC evidence |
| Planning and coordination | 13/15 (86.7%) | 30/59 (50.8%) | 43/74 (58.1%) | +35.9 | Reported more frequently in PHC evidence |
| Information and knowledge management | 11/15 (73.3%) | 23/59 (39.0%) | 34/74 (45.9%) | +34.3 | Reported more frequently in PHC evidence |
| Health infrastructure and logistics | 10/15 (66.7%) | 33/59 (55.9%) | 43/74 (58.1%) | +10.8 | Comparable reporting frequency across evidence sets |
| Monitoring and evaluation | 10/15 (66.7%) | 18/59 (30.5%) | 28/74 (37.8%) | +36.2 | Reported more frequently in PHC evidence |
| Health and related services | 8/15 (53.3%) | 23/59 (39.0%) | 31/74 (41.9%) | +14.3 | Reported with moderate frequency difference in PHC evidence |
| Policies, strategies and legislation | 7/15 (46.7%) | 17/59 (28.8%) | 24/74 (32.4%) | +17.9 | Reported with moderate frequency difference in PHC evidence |
| Community capacities for Health EDRM | 6/15 (40.0%) | 19/59 (32.2%) | 25/74 (33.8%) | +7.8 | Comparable reporting frequency across evidence sets |
| Financial resources | 5/15 (33.3%) | 9/59 (15.3%) | 14/74 (18.9%) | +18.0 | Reported with moderate frequency difference in PHC evidence |
| Risk communications | 4/15 (26.7%) | 13/59 (22.0%) | 17/74 (23.0%) | +4.7 | Comparable reporting frequency across evidence sets |

## Supplementary Table S12. Documentation and finalization items

| **Item** | **Available evidence** | **Boundary** | **Treatment** |
| --- | --- | --- | --- |
| Twenty-one report records classified as reports not retrieved | Watase identity/DOI ledger; publisher and bibliographic records; legitimate-access retrieval documentation | Full-text copies were not secured for local verification through the access routes available at the update stage. One identity was already represented through a separately obtained copy; eligibility could not be assessed for the other 20. | Report transparently as a historical Scopus retrieval-pathway limitation; do not describe the publications as nonexistent or undiscoverable. No JBI/MMAT appraisal or determinant coding was performed for the 20 unassessed identities. |
| Database search date | PubMed search: 27 June 2026; Scopus, ProQuest, and OpenAlex searches: 30 June 2026 | Database-specific execution dates were documented and reported in the manuscript and Supplementary Table S1 | Reported explicitly in the manuscript and Supplementary Table S1; this item is no longer considered a documentation limitation. |
| Retrieval-update reviewer status | Full-text decisions and item-level appraisal were independently confirmed by two reviewers | Second-reviewer confirmation completed and recorded | Consensus completed; preserve the signed or dated consensus record in the OSF amendment log. |
| Retrospective registration | Retrospective registration completed and described in the manuscript; persistent DOI: 10.17605/OSF.IO/H8JU7; associated project: https://osf.io/7atku/ | The record is explicitly retrospective rather than a preregistration; the DOI and associated project are available for reporting. | Use https://doi.org/10.17605/OSF.IO/H8JU7 consistently in the manuscript, PRISMA checklist, cover letter, and submission form. Associated project: https://osf.io/7atku/ |
| Primary-study appraisal status | All 15 primary empirical PHC studies were independently appraised from full text by SNI and TR using design-specific JBI tools or MMAT 2018. | All item-level judgments were resolved by full-text rechecking and consensus; no pending records remain. | Final item-level consensus tables are reported in Supplementary Table S7 and preserved in the OSF appraisal record. |
| Q1–Q4 tier-filter audit | All 24 record identities were reconstructed and reconciled to the original Scopus export | One primary empirical PHC study met eligibility criteria; 23 records were excluded with record-specific reasons | Final eligibility is based on prespecified criteria rather than journal quartile; preserve the completed audit ledger in the OSF amendment record. |
| Interpretation of MoUs and formal interorganizational agreements | Primary evidence reports referral agreements, transport arrangements, and multisectoral cooperation; the term MoU is not used consistently across studies. | MoU is an operational form of a formal agreement and should not be treated as a separate Health EDRM component or as a new source-level determinant unless explicitly reported. | Classify primarily under planning and coordination (PC). Also map to policies, strategies and legislation (PSL) only when the agreement establishes formal authority or institutional obligations. This interpretive clarification does not change component-frequency counts. |

# References reproduced for newly retrieved reports, the Q1–Q4 filter-audit inclusion, and appraisal tools

14. Dg Sirua MF, Balqis, Arifin MA, Indar, Manyullei S. The impact of nurses' disaster preparedness competencies on public health center resilience in disaster-prone areas: a cross-sectional study. Asia Pac J Health Manag. (2025) 20(1):i3973. doi: 10.24083/apjhm.v20i1.3973.

15. Derese TN, Koyra ZF, Mario LD, Mideksa HS, Dessie SA, Berihun AA. Exploring barriers and associated factors in the preparedness for cholera outbreak response in Public Health Centres of Addis Ababa, Ethiopia. PLOS Glob Public Health. (2025) 5(9):e0005087. doi: 10.1371/journal.pgph.0005087.

22. Firdausi N, Lestari F, Ismiyati A. Disaster preparedness analysis of public health centers in DKI Jakarta Province in 2020. Int J Saf Secur Eng. (2021) 11(1):91-99. doi: 10.18280/ijsse.110110.

23. Van Hoang M, Tran AT, Vu TT, Duong TK. COVID-19 preparedness and response capability: a case study of the Hanoi primary healthcare system. Health Serv Insights. (2021) 14:11786329211019224. doi: 10.1177/11786329211019224.

76. Sharifian S, Ghomian Z, Khodadadizadeh A, Jahangiri K. Assessment of disaster mitigation and preparedness. Trauma Mon. (2017) 22(2):e41082. doi: 10.5812/traumamon.41082.

77. Rahmati-Najarkolaei F, Moeeni A, Ebadi A, Heidaranlu E. Assessment of a military hospital's disaster preparedness using a Health Incident Command System. Trauma Mon. (2017) 22(2):e31448. doi: 10.5812/traumamon.31448.

78. Theron E, Smith WP. Emergency preparedness and disaster management for primary health care in Africa from the perspective of climate hazards and extreme weather events. Afr J Prim Health Care Fam Med. (2026) 18(1):a5449. doi: 10.4102/phcfm.v18i1.5449.

79. Adhikari SK, Shrestha A, Phuyal N, Bajracharya S, Shah PK, Katwal S, et al. Assessment of disaster preparedness planning in 25 hub hospitals of Nepal. J Nepal Health Res Counc. (2025) 23(67):369-376. doi: 10.33314/jnhrc.v23i02.4703.

80. Petrazzuoli F, Gokdemir O, Antonopoulou M, Blahova B, Mrduljas-Dujic N, Dumitra GG, et al. Resilience of primary healthcare facilities: experiences from 16 European countries during the COVID-19 pandemic. A mixed-methods study conducted by EURIPA. Adv Clin Exp Med. (2025) 34(4):487-505. doi: 10.17219/acem/194212.

81. Paganini M, Borrelli F, Cattani J, Ragazzoni L, Djalali A, Carenzo L, et al. Assessment of disaster preparedness among emergency departments in Italian hospitals: a cautious warning for disaster risk reduction and management capacity. Scand J Trauma Resusc Emerg Med. (2016) 24:101. doi: 10.1186/s13049-016-0292-6.

Hong QN, Fàbregues S, Bartlett G, Boardman F, Cargo M, Dagenais P, et al. The Mixed Methods Appraisal Tool (MMAT) version 2018 for information professionals and researchers. Educ Inf. 2018;34(4):285–291. doi: 10.3233/EFI-180221.
Hong QN, Pluye P, Fàbregues S, Bartlett G, Boardman F, Cargo M, Dagenais P, Gagnon MP, Griffiths F, Nicolau B, O’Cathain A, Rousseau MC, Vedel I. Mixed Methods Appraisal Tool (MMAT), version 2018. Registration of Copyright No. 1148552, Canadian Intellectual Property Office, Industry Canada.

JBI. Critical appraisal tools. Adelaide: JBI. Design-specific checklists applied according to the reported study design.

# Supplementary Table S13. Main-text citation traceability based on the source-derived coding in Supplementary Table S5

**Supplementary Table S13 reproduces the source-derived coding basis and complete Health EDRM mapping for the contextual publications included in Supplementary Table S5 and records where each publication is cited in the main manuscript. The table is a traceability aid and does not create new evidence. The "Reference no. in main manuscript" column retains the numbering used in the main manuscript reference list. Consequently, the numbering is not consecutive because references 1–2 are framework documents, references 9–12 are methodological and reporting sources, references 13–27 are primary empirical PHC studies, and reference 28 is the Hospital Safety Index guide. These sources are not repeated in this contextual-evidence traceability table.**

| **Reference no. in main manuscript** | **Study/source** | **DOI/identifier** | **Evidence type** | **Exact source-derived coding basis** | **Health EDRM mapping** | **Main-text citation location** |
| --- | --- | --- | --- | --- | --- | --- |
| 3 | Lamberti-Castronuovo et al. (2022) | 10.1016/j.ijdrr.2022.103278 | Systematic literature review | PSL: integrated PHC–H-EDRM strategy; PC: coordination and continuity planning; HR: multidisciplinary team; IKM: health information systems and telemedicine; HRS: continuity of care | PSL; PC; HR; IKM; HRS | §1; §3.3; §3.4; §4.2; §4.4 |
| 4 | Ward et al. (2024) | 10.1016/j.outlook.2024.102235 | Final citation: empirical nursing study | PC: pre-disaster network coordination; HR: clinical leadership, teamwork, and staff support; FR: preparedness resource availability; HIL: medical products and technology | PC; HR; FR; HIL | §1; §3.3; §3.4; §4.4 |
| 5 | Simsek et al. (2024) | 10.1016/j.pdisas.2024.100339 | Scoping review | PC: evacuation planning and provider roles; IKM: geographic information and preparedness data; HIL: access and evacuation infrastructure; CC: vulnerable-population and family education; ME: preparedness assessment | PC; IKM; HIL; CC; ME | §1; §3.3; §3.4; §4.4 |
| 6 | van der Heijden et al. (2022) | 10.3389/fpubh.2022.1035212 | Systematic review | PSL: water-emergency policy and standards; PC: stakeholder planning with water authorities and suppliers; HIL: basic WASH and emergency water infrastructure; CC: water-authority and supplier engagement | PSL; PC; HIL; CC | §1; §3.3; §3.4; §4.4 |
| 7 | Almukhlifi et al. (2022) | 10.1111/jocn.15965 | Integrative review | HR: education and training, prior disaster experience, and employment status | HR | §1; §3.3; §3.4; §4.4 |
| 8 | Su et al. (2023) | 10.1016/j.nepr.2023.103583 | Systematic review | HR: declarative and procedural knowledge from structured education; HRS: systematic educational and mental-health support | HR; HRS | §1; §3.3; §4.4 |
| 29 | Singh et al. (2026) | 10.1186/s12873-026-01474-2 | Scoping review | PSL: preparedness standards and safety policy; IKM: preparedness assessment instruments and data; HIL: structural, non-structural, and functional safety; ME: comparative preparedness assessment | PSL; IKM; HIL; ME | §3.3; §4.2 |
| 30 | Ndembi et al. (2024) | 10.1186/s12992-024-01066-4 | Comment; rapid literature and desk review with expert consultation | PSL: governance and harmonization of regional and global pandemic prevention, preparedness and response (PPPR) financing mechanisms; FR: sustainable and equitable financing for resilient health systems and access to medical countermeasures. | PSL; FR | §3.3; §4.4 |
| 31 | Awoyale et al. (2026) | 10.1038/s41598-025-34702-z | Cross-sectional | HR: risk literacy and response capacity; RC: risk communication; HIL: WASH, transport, and hazard-exposed infrastructure; CC: interagency and community coordination | HR; RC; HIL; CC | §3.3; §3.4 |
| 32 | Alfaifi and Hamaideh (2026) | 10.1016/j.pdisas.2026.100630 | Analytical cross-sectional | HR: nurses’ disaster knowledge, training, and experience | HR | §3.3 |
| 33 | Yadav et al. (2026) | 10.1016/j.pdisas.2026.100613 | Cross-sectional | PSL: legal and accreditation frameworks; PC: institutional preparedness procedures; HR: workforce education and preparedness culture; FR: preparedness resources and financing; HIL: facility infrastructure; HRS: emergency service readiness; ME: accreditation and compliance assessment | PSL; PC; HR; FR; HIL; HRS; ME | §3.3; §4.2 |
| 34 | Natali et al. (2025) | 10.1002/rhc3.70038 | Qualitative policy study | PSL: multilevel governance and policy design; PC: policy coordination and implementation; FR: recovery and resilience financing | PSL; PC; FR | §3.3 |
| 35 | Rao et al. (2025) | 10.1136/tsaco-2024-001552 | Expert opinion | PC: operational planning and coordination; HR: ethical decision-making, triage, and training; HIL: resources for austere response; CC: coordination with government and humanitarian actors | PC; HR; HIL; CC | §3.3 |
| 36 | Alrabie et al. (2025) | 10.3390/healthcare13131571 | Systematic review | PC: standard disaster protocols; HR: education, workshops, and disaster-field experience; HIL: medical resources and logistics | PC; HR; HIL | §3.3 |
| 37 | Hauta et al. (2025) | 10.3390/healthcare13182257 | Cross-sectional survey | PC: institutional disaster plans and collaboration; HR: knowledge, attitudes, and emergency-management experience; ME: preparedness assessment | PC; HR; ME | §3.3 |
| 38 | Abebe et al. (2025) | 10.1016/j.ijdrr.2025.105340 | Systematic review | PC: flood evacuation and preparedness plans; FR: resource allocation for flood resilience; HIL: backup generators and flood-resilient infrastructure | PC; FR; HIL | §3.3; §3.4 |
| 39 | Hong et al. (2025) | 10.3390/ijerph22020155 | Secondary analysis | RC: disaster risk perception and communication; HIL: material preparedness resources and supplies | RC; HIL | §3.3 |
| 40 | Tran et al. (2025) | 10.7189/jogh.15.03018 | Conceptual framework | HR: health-worker digital and data literacy; IKM: interoperable human–animal health data and early warning | HR; IKM | §3.3; §3.4; §4.4 |
| 41 | Majambo et al. (2025) | 10.4314/rmj.v82i1.8 | Pilot cross-sectional | HIL: emergency medicines and equipment in dental practices; HRS: dental-practice readiness for medical emergencies. | HIL; HRS | §3.3 |
| 42 | Cvetkovic et al. (2024) | 10.3390/healthcare12191962 | Analytical cross-sectional | PSL: standards and policy compliance; PC: EMS organization and procedures; HR: staffing and continuing education; HIL: equipment and vehicles; HRS: emergency response services; ME: response-time and performance assessment | PSL; PC; HR; HIL; HRS; ME | §3.3; §4.2 |
| 43 | Lee and Lee (2024) | 10.3390/healthcare12151484 | Spatial analysis | PSL: public-policy effectiveness; HIL: spatial distribution and robustness of health infrastructure | PSL; HIL | §3.3 |
| 44 | Aggarwal et al. (2024) | 10.2196/57384 | Qualitative study | HRS: stepped-care and service-delivery response; CC: rural access and community needs | HRS; CC | §3.3; §3.4 |
| 45 | Tang (2024) | 10.1080/19401493.2024.2395269 | Agent-based modelling | IKM: machine-learning-supported modelling of airborne infection risk in healthcare facilities. | IKM | §3.3; §3.4; §4.4 |
| 46 | Guha et al. (2025) | 10.1136/bmjgh-2023-013392 | Mixed methods | PSL: national policy and governance; HR: workforce adaptation; HRS: continuity of essential health services; CC: community- and patient-level continuity measures | PSL; HR; HRS; CC | §3.3 |
| 47 | Elaidy et al. (2023) | 10.1186/s43045-023-00285-6 | Analytical cross-sectional | HR: staff mental health, coping, and employment resilience; HIL: availability of PPE | HR; HIL | §3.3 |
| 48 | Noboa-Ramos et al. (2023) | 10.1017/dmp.2022.272 | Qualitative study | HIL: hurricane-related disruptions to power, water, communications, and critical supplies; HRS: effects on healthcare delivery and organizational recovery; CC: coordination between healthcare and social organizations and attention to patient social needs. | HIL; HRS; CC | §3.3; §3.4 |
| 49 | Mukherji et al. (2022) | 10.5005/jp-journals-10005-2353 | Descriptive cross-sectional | PC: governance, coordination, and risk-sharing arrangements in public-private partnership-supported primary care; CC: community-oriented primary-care service delivery through partnership arrangements. | PC; CC | §3.3; §3.4; §4.2; §4.4; §4.6 |
| 50 | Maghfiroh and Hanaoka (2022) | 10.3926/jiem.3941 | Stochastic modelling | HIL: mobile-clinic logistics and strategic location; HRS: mobile medical-service delivery | HIL; HRS | §3.3; §4.4 |
| 51 | Pawar et al. (2024) | 10.1016/j.ijdrr.2024.104437 | Systematic review | PC: domain-specific preparedness and coordination; HR: human resources and personnel protection; FR: financial preparedness; RC: communication; HIL: logistics and evacuation resources | PC; HR; FR; RC; HIL | §3.3; §4.2 |
| 52 | Baker (2021) | 10.1016/j.ienj.2021.100993 | Cross-sectional | PC: Incident Command System; HR: disaster terminology, triage ethics, and decontamination; IKM: surveillance; HIL: access to critical resources; HRS: nursing disaster-response readiness | PC; HR; IKM; HIL; HRS | §3.3 |
| 53 | Xiang et al. (2021) | 10.1016/j.ijdrr.2021.102072 | Document analysis | PSL: language-access policy; HR: limited-English-proficiency and language capacity; CC: community language access | PSL; HR; CC | §3.3 |
| 54 | George and Anilkumar (2021) | 10.1016/j.ijdrr.2021.102077 | Index development | PC: operational standard operating procedures; RC: coordination and communication indicators | PC; RC | §3.3 |
| 55 | Shiroya et al. (2021) | 10.3390/ijerph18095044 | Mixed methods | IKM: medical records and HMIS; HIL: oxygen and facility readiness; HRS: continuity and integration of NCD services | IKM; HIL; HRS | §3.3; §3.4; §4.2 |
| 56 | Palinkas et al. (2021) | 10.3390/ijerph18020425 | Qualitative rapid assessment | PC: search-and-rescue and blended pandemic–storm coordination; HRS: rapid preparedness-needs and resource response; CC: community resource assessment | PC; HRS; CC | §3.3; §3.4 |
| 57 | Pourmohammadi et al. (2021) | 10.1017/dmp.2021.104 | Cross-sectional | PC: functional preparedness plan; HR: rapid-response team; FR: facility insurance and financial protection; IKM: hazard mapping; ME: facility preparedness assessment | PC; HR; FR; IKM; ME | §3.3 |
| 58 | Li et al. (2021) | 10.3389/fpubh.2021.764069 | Scientometric review | PC: governance and emergency-team organization; HR: emergency-team formation; IKM: scientometric knowledge mapping; HIL: adequacy of physical resources; ME: resilience assessment | PC; HR; IKM; HIL; ME | §3.3 |
| 59 | Wuthisuthimethawee et al. (2021) | 10.2147/RMHP.S314391 | Tool development | PSL: national standards embedded in the assessment tool; PC: mass-casualty and disaster plans; HR: health workforce and leadership; FR: emergency financing; IKM: information systems; HIL: medical products and technology; CC: community and pediatric considerations | PSL; PC; HR; FR; IKM; HIL; CC | §3.3; §4.2 |
| 60 | Ibrahim et al. (2021) | 10.1186/s13031-021-00412-2 | Qualitative study | HR: health-system workforce resilience and familiarity | HR | §3.3 |
| 61 | Gilmore et al. (2020) | 10.1136/bmjgh-2020-003188 | Rapid evidence review | RC: community communication and social-mobilization approaches for infectious-disease prevention and control; CC: community actors, participatory engagement, local ownership, and linkages with health-system stakeholders. | RC; CC | §3.3; §3.4; §4.5 |
| 62 | Oppong et al. (2020) | 10.2147/IDR.S273851 | Cross-sectional | HR: infection-prevention workforce preparedness; HIL: PPE and sterile equipment; HRS: infection-prevention and control service readiness | HR; HIL; HRS | §3.3; §3.4 |
| 63 | Hierink et al. (2020) | 10.1136/bmjopen-2020-039138 | Geospatial modelling | IKM: GIS, travel-time, and accessibility modelling | IKM | §3.3; §3.4; §4.4 |
| 64 | Brewer et al. (2020) | 10.1016/j.auec.2019.12.005 | Cross-sectional survey | HR: trauma, triage, leadership, and psychological preparedness; RC: regional and rural emergency communication; HRS: emergency-department response readiness | HR; RC; HRS | §3.3 |
| 65 | Sharma and Sharma (2020) | 10.1177/0972063420935539 | Descriptive cross-sectional | PC: critical-stabilization and isolation procedures; HR: staff retraining; HIL: isolation rooms and medication stock; HRS: critical-stabilization services; CC: community health-centre linkage | PC; HR; HIL; HRS; CC | §3.3 |
| 66 | Sultan et al. (2020) | 10.3390/healthcare8040442 | Analytical cross-sectional; identity reconciled to Sultan et al. | HR: preparedness knowledge, confidence, willingness to work, and own/family safety considerations; IKM: self-rated knowledge and perceptions of risk and danger. | HR; IKM | §3.3; §3.4 |
| 67 | Wagner et al. (2021) | 10.1097/PCC.0000000000002649 | Multicentre cross-sectional | HR: simulation-based pandemic and disaster readiness | HR | §3.3; §3.4; §4.4 |
| 68 | Sheikhbardsiri et al. (2019) | 10.1097/PHH.0000000000000815 | Descriptive cross-sectional | PC: operational exercise design and coordination; IKM: scenario information and operational uncertainty; RC: communication during the exercise; HIL: disaster-response resources; ME: exercise-based preparedness evaluation. | PC; IKM; RC; HIL; ME | §3.3; §3.4; §4.4 |
| 69 | Ballard et al. (2022) | 10.9745/GHSP-D-21-00648 | Commentary | HR: trained, supervised, remunerated, and integrated community health worker workforce; RC: household risk communication and trusted community education; HIL: personal protective equipment, essential commodities, and supply-chain support; HRS: maintenance of community-based essential health services, surveillance, testing referral, and home-based care; CC: community engagement and links between communities and health systems; ME: community health worker data systems, performance monitoring, and evaluation. | HR; RC; HIL; HRS; CC; ME | §3.3; §3.4; §4.5 |
| 70 | Qari et al. (2019) | 10.1017/dmp.2018.110 | Systematic review/evaluation framework | PC: public-health preparedness-system coordination; HR: workforce capability; IKM: evaluation information; CC: system and community partners; ME: performance metrics | PC; HR; IKM; CC; ME | §3.3; §3.4; §4.4 |
| 71 | Thwala et al. (2018) | 10.1371/journal.pone.0194576 | Facility assessment | PC: emergency obstetric and newborn-care coordination; HR: staffing; HIL: facility equipment and supplies; HRS: emergency obstetric services | PC; HR; HIL; HRS | §3.3 |
| 72 | Sabegh et al. (2017) | 10.1007/s13198-017-0645-1 | Neural-network modelling | HR: operational capacity in the healthcare supply chain; HIL: green disaster-response supply chain; ME: multi-objective performance optimization | HR; HIL; ME | §3.3; §4.4 |
| 73 | Lucchini et al. (2017) | 10.1186/s12889-016-3939-3 | Comparative review | IKM: exposure assessment; HRS: lifetime health care; CC: affected populations; ME: systematic preparedness and long-term monitoring | IKM; HRS; CC; ME | §3.3 |
| 74 | Powell et al. (2016) | 10.1016/j.ejor.2016.04.037 | Methodological paper | IKM: dynamic threat and risk information; ME: auditable risk identification and assessment | IKM; ME | §3.3; §3.4 |
| 75 | Bala et al. (2016) | 10.1109/JBHI.2015.2477371 | Qualitative case study | PC: integrated information-technology architecture for disaster-response coordination; IKM: health-data integration and patient/information tracking; RC: information-technology-enabled emergency communication. | PC; IKM; RC | §3.3; §3.4; §4.4 |
| 76 | Sharifian et al. (2017) | 10.5812/traumamon.41082 | Qualitative content analysis of national documents | Policy, planning, workforce development, financing, information, early warning, infrastructure, community education and evaluation. | PSL; PC; HR; FR; IKM; RC; HIL; CC; ME | §3.3 |
| 77 | Rahmati-Najarkolaei et al. (2017) | 10.5812/traumamon.31448 | Analytical cross-sectional facility/staff assessment with maneuver observation | Incident command, roles, staff training, internal communication, logistics, triage, patient tracking and exercise evaluation. | PSL; PC; HR; IKM; HIL; HRS; ME | §3.3; §4.2 |
| 78 | Theron and Smith (2026) | 10.4102/phcfm.v18i1.5449 | Textual expert article with case example | Risk governance, multisectoral planning, workforce capacity, hazard assessment, early warning, utilities, service continuity, community participation and evaluation. | PSL; PC; HR; IKM; RC; HIL; HRS; CC; ME | §3.3; §3.4; §4.2; §4.4 |
| 79 | Adhikari et al. (2025) | 10.33314/jnhrc.v23i02.4703 | Observational descriptive secondary analysis | Plans, committees, staffing, reporting systems, communication contacts, infrastructure, utilities, supplies, triage, essential services and monitoring. | PSL; PC; HR; IKM; RC; HIL; HRS; ME | §3.3; §4.2 |
| 80 | Petrazzuoli et al. (2025) | 10.17219/acem/194212 | Cross-sectional mixed-methods survey | Guidance, coordination, workforce resilience, information, communication, PPE and telehealth, service adaptation and community resilience groups. | PSL; PC; HR; IKM; RC; HIL; HRS; CC | §3.3; §3.4; §4.2; §4.4 |
| 81 | Paganini et al. (2016) | 10.1186/s13049-016-0292-6 | Analytical cross-sectional telephone survey | Disaster plans, activation, command authority, staff roles and recall, information, surge arrangements, service response and periodic testing. | PSL; PC; HR; IKM; HIL; HRS; ME | §3.3; §4.2 |

# Supplementary Table S14. Evidence-to-placement matrix for the proposed layered model of PHC preparedness.

| **Functional domain or element** | **Health EDRM component(s)** | **Representative primary PHC evidence** | **Representative contextual evidence** | **Placement rationale** |
| --- | --- | --- | --- | --- |
| System Support | Policies, strategies and legislation; Financial resources | Institutional coordination, preparedness plans, enabling arrangements, and resource constraints reported in PHC settings (15, 17, 22, 23). | Preparedness assessment and safety policy (29); PPPR financing and governance (30); legal and accreditation frameworks (33); multilevel policy coordination and resilience financing (34); preparedness indicators and governance (59). | Provides mandate, authority, financing, and procurement flexibility that enable—but do not unidirectionally determine—facility and district preparedness. |
| Core Preparedness Capacity | Planning and coordination; Human resources; Health infrastructure and logistics | Plans, staffing, training, teamwork, utilities, WASH, stocks, transport, and facility safety (13, 15-17, 20, 21, 24-27). | Emergency-water infrastructure and stakeholder planning (6); workforce education and prior experience (7); WASH, transport, and hazard-exposed infrastructure (31); flood-resilient infrastructure (38); hurricane-related utility and supply disruption (48); hospital preparedness needs assessment (51); simulation-based readiness (67); operational exercise evaluation (68); coordination and performance metrics (70); CHW workforce and supply support during pandemics (69). | Represents the minimum organizational, workforce, and physical capability required to sustain PHC operations. |
| Service and Community Response | Health and related services; Community capacities for Health EDRM | Referral, essential services, psychosocial care, outreach, service functionality, and community engagement (14, 17, 18, 22-25). | Vulnerable-population support (5); rural access and community needs (44); partnership-supported community-oriented primary care (49); continuity of noncommunicable-disease services (55); rapid community needs and resource assessment (56); community engagement for infectious-disease control (61); CHW-supported essential services and community engagement during pandemics (69); climate-related PHC continuity (78); telehealth and community resilience in European PHC (80). | Translates preparedness into continued access, care, referral, outreach, and partnership with communities. |
| Cross-Cutting Functions | Information and knowledge management; Risk communications; Monitoring and evaluation | Early warning, health information, repeated assessment, surveillance, exercises, and facility-safety monitoring (16, 17, 19, 20, 22-24, 26, 27). | Interoperable early-warning data (40); airborne-infection risk modelling (45); geographic information and accessibility modelling (63); preparedness performance metrics (70); auditable threat assessment (74); information-technology-enabled coordination and communication (75); community-engagement communication approaches (61); CHW risk communication and performance monitoring (69). | Operates across all domains to support detection, communication, adaptation, accountability, and learning. |
| Community participation across the cycle | Community capacities; Planning and coordination; Risk communications; Monitoring and evaluation | Collaboration, engagement, community coordination, and community-related safety capacity (14, 17, 22-24). | Provider roles for vulnerable populations (5); partnership-supported community delivery (49); rapid community-resource assessment (56); community engagement and local ownership in infectious-disease control (61); evaluation frameworks (70); CHW links between communities and health systems (69); climate-related community participation (78); community resilience groups (80). | Clarifies that communities are partners in risk assessment, planning, communication, implementation, monitoring, and evaluation—not only recipients of response services. |
| Continuity of Operations and Essential Services | Intended performance domain rather than an additional Health EDRM component | Operational capacity, service functionality, referral, utilities, staffing, and essential-service evidence (17, 20, 22-26). | Cross-country service continuity, noncommunicable-disease continuity, conflict-system resilience, climate continuity, and PHC adaptation (46, 55, 60, 78, 80). | Provides a measurable endpoint using operational status, service downtime, workforce and medicine availability, referral completion, restoration time, and access for priority populations. |

Note: Representative references are illustrative rather than exhaustive. Full publication-level source-derived indicators and coding traceability are provided in Supplementary Tables S8-S10. Domain placement reflects interpretive two-reviewer consensus within the author team and does not constitute formal external or empirical validation.
